# Drimane-Type Sesquiterpenoids Derived from the Tropical Basidiomycetes *Perenniporia centrali-africana* and *Cerrena* sp. nov

**DOI:** 10.3390/molecules27185968

**Published:** 2022-09-14

**Authors:** Paomephan Pathompong, Sebastian Pfütze, Frank Surup, Thitiya Boonpratuang, Rattaket Choeyklin, Josphat C. Matasyoh, Cony Decock, Marc Stadler, Chuenchit Boonchird

**Affiliations:** 1 Department of Biotechnology, Faculty of Science, Mahidol University, Bangkok 10400, Thailand; 2 Department of Microbial Drugs, Helmholtz Centre for Infection Research (HZI), German Centre for Infection Research (DZIF), Partner Site Hannover/Braunschweig, Inhoffenstrasse 7, 38124 Braunschweig, Germany; 3 Institute of Microbiology, Technische Universität Braunschweig, Spielmannstraße 7, 38106 Braunschweig, Germany; 4 National Biobank of Thailand (NBT), The National Science and Technology for Development Agency (NSTDA), Thailand Science Park, Pathum Thani 12120, Thailand; 5 Biodiversity-Based Economy Development Office (Public Organization) (BEDO), The Government Complex, Building Ratthaprasasanabhakti, Bangkok 10210, Thailand; 6 Department of Chemistry, Egerton University, P.O. Box 536, Njoro 20115, Kenya; 7 Earth and Life Institute, Mycothéque de l’ Universite Catholique de Louvain (BCCM/MUCL), Place Croix du Sud 3, B-1348 Louvain-la-Neuve, Belgium

**Keywords:** *Perenniporia centrali-africana*, *Cerrena* sp. nov., drimane-type sesquiterpenoids

## Abstract

Five new drimane-type sesquiterpenoids were isolated from cultures of the tropical basidiomycetes, *Perenniporia centrali-africana* (originating from Kenya) and *Cerrena* sp. nov. (originating from Thailand). A new pereniporin A derivative (**1**), a new drimane-type sesquiterpene lactam (**2**), and the new 6,7-Dehydro-isodrimenediol (**3**) were isolated from *P. centrali-africana.* In parallel, the two new drimane-type sesquiterpene lactams **5** and **6** were isolated together with known isodrimenediol (**4**) from *Cerrena* sp. This is the first report of drimane-type sesquiterpene lactams from basidiomycetes. The structures were elucidated based on 1D and 2D nuclear magnetic resonance (NMR) spectroscopic data, in combination with high-resolution electrospray mass spectrometric (HR-ESIMS) data. The compounds were devoid of significant antimicrobial and cytotoxic activities.

## 1. Introduction

Mushroom-forming fungi (mostly belonging to the division Basidiomycota) have been investigated regarding their abilities as prolific producers of structurally diverse bioactive compounds, leading to the discovery of numerous unique highly functionalized secondary metabolites [1,2]. Although the species of temperate climate zones have already been studied rather intensively, the endemic Basidiomycota of tropical countries such as Thailand have recently been found to be a good source for unprecedented metabolites [3,4]. New bioactive molecules have also been reported from Kenyan Basidiomycota that represent undescribed species [5,6].

The current study deals with the production, isolation, and characterization of further new sesquiterpenoid derivatives from two fungal species originating from Kenya and Thailand, respectively, which were examined in our ongoing projects aiming at the discovery of novel antibiotics and other beneficial molecules from Basidiomycota. These strains were selected for intensified evaluation because preliminary studies had revealed weak to moderate antimicrobial effects in the crude extracts, along with the presence of potentially undescribed molecules, as deduced from HPLC–DAD/MS (high performance liquid chromatography coupled with diode array detection and mass spectrometry) data. Since the molecules turned out to be structurally similar, we decided to report their isolation and structure elucidation in one paper and include data on the taxonomy of the producer strain.

## 2. Results and Discussion

### 2.1. Fungal Identification and Crude Extracts

Perenniporia strain MUCL 56,028 was identified by comparison of morphological characteristics and sequencing of the 5.8S/ITS nrDNA, as described in the Experimental Section (the sequence as shown in Table S1 in the Supporting Information). *P. centrali-africana* is a common white rot, wood decay basidiomycete belonging to the family, Polyporaceae. This fungus has a dimitic or trimitic hyphal structure with clamp connections on generative hyphae. It produces ellipsoid to distinctly truncate soft and thick-walled basidiospores [7]. A BLAST search in GenBank confirmed the generic affinities of the strain to the genus *Perenniporia* in the family Polyporaceae by a closest hit (GenBank acc. no KX584430.1) (*Perenniporia centrali-africana*) with 99.85% identity (cf. Figure S1 in the Supporting Information). The crude extracts of the strain MUCL 56,028 exhibited antimicrobial activity against Bacillus subtilis, with a minimum inhibitory concentration (MIC) of 75 μg/mL (compared to the positive control ciprofloxacin at an MIC of 3.1 μg/mL).

Strain BCC 84,628 was identified using a comparison of morphological characteristics and sequencing of the 5.8S/ITS nrDNA region (the sequence is shown in Table S1 in the Supporting Information). The genus *Cerrena* is characterized by having a dimitic or trimitic hyphal system, nonamyloid, hyaline basidiospores, and its species are classified as white rot fungi [8]. This genus is known as an enzyme production machinery due to its decomposing capacity in biological niches [9,10,11,12]. A BLAST search in GenBank confirmed that the strain belongs to the genus *Cerrena* in the Cerrenaceae. Based on the basidiocarps and culture morphology, two of the authors (T.B. and R.C.) classified this fungus as *Cerrena* cf. *caperata*. The ITS nRDNA sequence was deposited as GenBank acc. no MW512503. Further taxonomic studies including comparisons with type specimens of related species are ongoing to characterize this strain to the species level, but it constitutes an undescribed taxon according to the data presently available. The extracts of the strain BCC 84,628 exhibited activity against *Bacillus subtilis* with an MIC of 37.5 μg/mL (compared to the positive control ciprofloxacin at an MIC of 3.1 μg/mL).

### 2.2. Isolation and Structure Elucidation of Metabolites from Perenniporia centrali-africana

**Figure 1:**
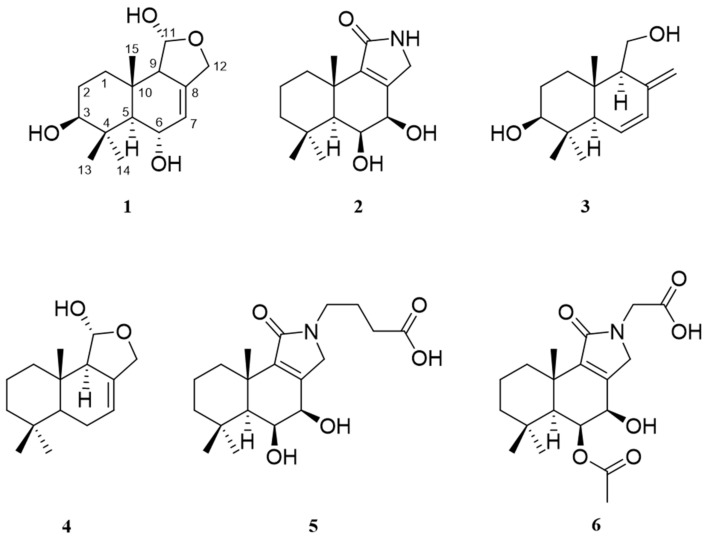
Chemical structures of compounds **1**–**3** from *Perenniporia centrali-africana* and compound **4**–**6** from *Cerrena* sp.

Extensive chromatography of the extracts obtained from *Perenniporia centrali-africana* (MUCL 56028) led to the isolation of three previously undescribed compounds, similar to the pereniporin core structure as previously described [13]. Alongside the new 3β-Hydroxy-9-dehydroxy-pereniporin A (**1**), 6,7-Dihydroxy-12-deoxy-dysidealactam (**2**), and 6,7-Dehydro-isodrimenediol (**3**), the known pereniporin A and other derivatives including 6-O-acetylpereniporin A, 3β-hydroxy-6-O-acetylpereniporin A, 11α-Hydroxycinnamosmolide, 6α,9α,11α-trihydroxycinnamolide, and sulphureuine H were obtained from *P. centrali-africana* and were identified by comparing their NMR data (Table 1) with those reported in the literature [13,14,15,16,17]. The structures of **1**–**3** are shown in Figure 1. Furthermore, the extracts of rice standing cultures led to the isolation of the three previously described cryptoporic acids B, H, and I [18,19]. The structures of the known compounds are shown in the Supporting Information (Figure S2).

3β-Hydroxy-9-dehydroxy-pereniporin A (**1**) was isolated as white solid from the supernatant extract, after liquid cultivation of *Perenniporia* sp. in BAF medium. Its molecular formula was deduced from HRESIMS data, according to the molecular ion cluster at *m*/*z* 291.1565 [M + Na]^+^ (calcd. for C_15_H_24_NaO_4_ 291.1567), indicating four degrees of unsaturation. ^1^H NMR and HSQC data (Table 1, Figure S7) led to the identification of three methyls at δ_H_ 0.85 (s, H_3_-15), 0.99 (s, H_3_-14), and 1.23 (s, H_3_-13), three methylenes at δ_H_ 1.44 (m, H-1α), 1.70 (m, H-1β), 1.67 (m, H_2_-2), 4.18 (br dd, *J* = 12.05, 1.08 Hz, H-12β), and 4.46 (m, H-12α), two methines at δ_H_ 1.31 (d, *J* = 8.90 Hz, H-5) and 2.25 (m, H-9), three oxymethines at δ_H_ 3.22 (dd, *J* = 11.4, 4.5 Hz, H-3), 4.34 (br d, *J* = 8.90 Hz, H-12), and 5.17 (d, *J* = 4.5 Hz, H-11), and one olefinic methine at δ_H_ 5.53 (m, H-7). The ^13^C and HMBC NMR data (Table 1, Figure S8) revealed the presence of 15 carbon resonances, including two sp^2^-hybridized carbons, comprising one nonprotonated carbon (δ_C_ 139.1, C-8) and one methine (δ_C_ 120.3, C-7); three oxymethines (δ_C_ 98.7, C-11; 78.2, C-3; 67.4, C-6); two methines (δ_C_ 60.6, C-9; 57.0, C-5); three methylene carbons (δ_C_ 37.2, C-1; 26.5, C-2; 67.7, C-12); and three methyl carbons (δ_C_ 29.5, C-14; 15.1, C-13; 14.3, C-15). One degree of unsaturation is given by the two sp^2^-hybridized carbons, suggesting three rings in the scaffold of 3β-Hydroxy-9-dehydroxy-pereniporin A (**1**). Analysis of the ^1^H-^1^H COSY data gave the first spin system due to correlations between H_2_-1, H_2_-2, and H-3. Correlations between H-5, H-6, and H-7, as well as between H-9 and H-11, led to the identification of the second and third spin system. HMBC correlations from H_3_-13/H_3_-14 to C-3/C-4/C-5, H_3_-15 to C-1/C-5/C-9/C-10, and H-5 to C-3/C-4/C-5/C-6/C-7/C-8/C-9/C-10/C-15 revealed the presence of two six-membered rings that were fused across C-5 and C-10, carrying a hydroxyl function at C-3 and C-6, as well as one methyl group at C-10 and two methyl groups at C-4. Further HMBC correlations from H-11 to C-8/C-9/C-12 and from H_2_-12 to C-7/C-8/C-9/C-11 led to the identification of a tetrahydrofuran-11-ol substructure that was fused to the molecule across C-8 and C-9. Relative configuration could be obtained by evaluation of ROE data. Due to key correlations among H-1α/H-3α/H-5α/H-9α/H-12α/H_3_-14, these protons were arbitrarily assigned to the α face of the molecule. Correlations between H-1β/H-6β/H-11β/H-12β/H_3_-13/H_3_-15 indicated a β orientation of these protons. Finally, the absolute configuration of **1** was determined as 3*S*,5*R*,6*S*,9*R*,10*S*,11*R*, after comparison of its ECD data to the ones of related drimane derivatives [8]. Furthermore, these compounds were isolated from the same genus (*Perenniporia*) and showed similar ROESY correlations.

6,7-Dihydroxy-12-deoxy-dysidealactam (**2**), which is closely related to **1**, was obtained as white solid. Its molecular formula was assigned as C_15_H_23_NO_3,_ according to the molecular ion cluster at *m*/*z* 266.1750 [M + H]^+^ in the HRESIMS spectrum, indicating the presence of a nitrogen atom. One- and two-dimensional NMR spectra revealed the absence of the hydroxyl function at C-3, which was replaced by a methylene (δ_H_ 1.25, H-3β; 1.44, H-3α). ^1^H NMR, ^13^C, and HSQC data indicated the presence of another oxymethine (δ_C_ 70.9; δ_H_ 4.10) at C-7, which was confirmed by HMBC correlations from H-7α to C-5/C-6/C-8/C-9/C-12. Furthermore, a lactam functionality was placed at C-11 (δ_C_ 173.4), which was supported by the absence of H-11, the chemical shift of the methylene CH_2_–12 (δ_C_ 46.7, δ_H_ 3.73, 4.00), and the HMBC correlations of H_2_-12 to C-11. Metabolite **2** is the nitrogen derivative of deacetylugandensolide. Since **2** and deacetylugandensolide have very similar coupling constants and chemical shifts for H-5, H-6 and H-7, we propose a common relative configuration [20].

6,7-Dehydro-isodrimenediol (**3**) was isolated as white solid with a molecular formula of C_15_H_24_O_2_ and showed similarities to **1**, according to its 1D and 2D NMR data. ^1^H NMR and HSQC data indicated the presence of another protonated sp^2^-hybridized carbon (δ_H_ 6.20, H-6; δ_C_ 133.3, C-6) replacing oxymethine H-6 and the presence of oxymethylene and exometylene moieties at C–11 and C–12 as key differences, respectively.

Previous reports showed that *Perenniporia* spp. generally produce drimane-type sesquiterpenoids called pereniporins, which is somewhat unfortunate because of the typo in the trivial name. Pereniporins A and B were firstly isolated in 1986 from *P. medulla-panis* [17]. Several derivatives of pereniporin A have been isolated from *P. maackiae* [13]. In this study, two further derivatives of pereniporin A were isolated from a submerged cultivation of *P. centrali-africana*. Solid cultivation of *P. centrali-africana* led to the isolation of the known cryptoporic acids B, I, and H. Cryptoporic acid B was firstly isolated from *Cryptoporus volvatus* along with cryptoporic acid A [18], while cryptoporic acid H was previously isolated from mycelial cultures of *Ganoderma neo-japonicum* and *C. volvatus.* Later, Cabrera et al. isolated cryptoporic acid H from the culture broth of *Polyporus ciliatus* [21] (current name *Ceriporus ciliatus*).

### 2.3. Isolation and Structure Elucidation of Compounds ***4**–**6*** from Cerrena sp.

Extensive chromatography of the extracts obtained from *Cerrena* sp. (BBH 40848) led to the isolation of two previously undescribed and two known compounds called cryptoporic acid H and isodrimeniol. Isodrimeniol (**4**) was isolated as a colorless oil. The molecular formula of **4** was determined as C_15_H_24_O_2_ by HRESIMS data. The planar structure was identified as isodrimeniol by 1D and 2D NMR data (see Table 2, Figures S27–S31 for details). Since the coupling constants of **4** are identical to those published by Rodriguez et al. [22], the stereochemistry of **4** was assigned to be identical to that of isodrimeniol, which was isolated from the bark of *Drimys winteri* Forst. The analysis of the HRESIMS spectrum of compound **5** yielded the molecular formula C_19_H_29_NO_5_. The ^1^H and ^13^C NMR data of **5** were very like those of **2**. The key differences were the presences of additional signals for three methylenes and one carboxylic moiety. Since the 2′–H_2_ and 3*′*–H_2_ showed HMBC correlations (Figure S25) to C–1*′* and 4*′*–H_2_ to C–11 and C–12, the structure of **5** was assigned as the butyric acid derivative of **2**. The molecular formula of **6** was determined by HRESIMS data as C_19_H_27_NO_6_. NMR data were highly similar to those of **2** and **5**. However, the key differences compared to **5** were an acetyl group attached to C–6 and a glycinyl moiety instead of the 4-aminobutyryl residue of **5**. Consequently, **6** is the glycinyl derivative of the known ugandensolide.

Previously, the cytotoxic sesquiterpenoids named cerrenins A–E were isolated from *Cerrena* sp. [23,24], which are so far the only secondary metabolites known from this genus. Interestingly, the new sesquiterpene lactams were isolated from two different families of Basidiomycota (i.e., Polyporaceae and Cerrenaceae). Sesquiterpene lactams have rarely been discovered as natural products, even though there are some reports from marine animals [25,26,27]. Another one was reported from the leaves of Cinnamosma fragrans, which revealed the first report of tyramine involved in a lactam formation from a sesquiterpene [28]. *Cerrena* produced two new sesquiterpene lactams (**5**, **6**), which show an addition of amino acids to the lactam ring. *P. centrali-africana* also produced one sesquiterpene lactam (**2**), but this metabolite lacked an amino acid at the lactam ring. According to molecular phylogenetic studies, these basidiomycetes are currently classified in the residual clade (Cerrenaceae) and core polyporoid clade of the Polyporaceae [29]. The strains may possess two different biosynthesis gene clusters that contain enzymes mediating aminations that add amino acids into the lactam ring of sesquiterpenes. As recently reported, dehydrogenation is an important step of closing the ring in drimane sesquiterpenoid formation by oxidoreductases [30]. *Cerrena* should accordingly have an aminase at this step to incorporate the amine group of amino acids, to form the lactam ring instead of dehydrogenation. However, this hypothesis remains to be validated by genome sequencing and subsequent experimental work that goes far beyond to the scope of our current study.

### 2.4. Biological Assays

Compounds **1**, **4**, **5**, and **6** were evaluated for antimicrobial and cytotoxic activities according to established procedures [31]. Compound **4** weakly exhibited antimicrobial activities against *Mucor hiemalis* and *Rhodoturula glutinis* (MIC 67µg/mL), and exhibited cytotoxicity of L929 (IC_50_ 33 µg/mL) and A549 (IC_50_ 16 µg/mL). A detailed report on their activity is given in the Supporting Information in Tables S4 and S5. According to previous reports, the functional group at C-6 is an important factor for enabling its cytotoxicity. For the cytotoxic pereniporins, the absolute configuration at C-6 was reported to be *S* [13]. None of the sesquiterpene lactams, including compound **5** and **6**, showed antimicrobial activities and cytotoxicity.

## 3. Materials and Methods

### 3.1. General Information

HPLC–DAD/MS measurements were performed using an amaZon speed ETD (electron transfer dissociation) ion trap mass spectrometer (Bruker Daltonics, Bremen, Germany) and measured in positive and negative ion modes simultaneously, with the HPLC system (column C18 Acquity UPLC BEH Waters, Eschborn, Germany); solvent A: water (H_2_O), solvent B: acetonitrile (can) supplemented with 0.1% formic acid (FA), gradient conditions: 5% B for 0.5 min, increasing to 100% B for 20 min, and maintaining isocratic conditions at 100% B for 10 min, with a flow rate of 0.6 mL/min, using UV/Vis detection (200–600 nm).

HR-ESIMS (high-resolution electrospray ionization mass spectrometry) data were recorded on a MaXis ESI-TOF (electrospray ionization-time of flight) mass spectrometer (Bruker Daltonics), coupled with an Agilent 1260 series HPLC-UV system and equipped with a C18 Acquity UPLC BEH (ultraperformance liquid chromatography) (ethylene bridged hybrid) (Waters) column; DAD-UV detection at 200–600 nm; solvent A (H_2_O) and solvent B (ACN) were supplemented with 0.1% FA as a modifier; a flow rate of 0.6 mL/min, at 40 °C, using a gradient elution system with the initial condition of 5% B for 0.5 min, increasing to 100% B for 19.5 min, and holding at 100% B for 5 min. Bruker Compass DataAnalysis 4.4 SR1 was used to analyze the data, including determining the molecular formula using the Smart Formula algorithm (Bruker Daltonics).

One- and two-dimensional NMR spectra were measured on a Bruker 700 MHz Avance III spectrometer, equipped with a 5 mm TCI cryoprobe (^1^H: 700 MHz, ^13^C: 175 MHz), and a Bruker Avance III 500 (^1^H 500 MHz, ^13^C 125 MHz) spectrometer. NMR data were referenced to selected chemical shifts of acetone-*d*_6_ (^1^H: 2.05 ppm, ^13^C: 29.32 ppm) and CH_3_OH-*d*_4_ (^1^H: 3.31 ppm, ^13^C: 49.15 ppm), respectively. Optical rotations were measured using an Anton Paar MCP-150 Polarimeter (Graz, Austria), with a 100 mm path length and a sodium D line at 589 nm. The UV spectra were measured on a Shimadzu (Kyoto, Japan) UV/Vis 2450 spectrophotometer using methanol (Uvasol, Merck, Darmstadt, Germany) as a solvent. ECD (electronic circular dichroism) spectra were measured with a J-815 spectropolarimeter (Jasco, Pfungstadt, Germany) using methanol as a solvent. The spectral data are combined in the Supplementary Information (Figures S3–S72).

### 3.2. Fungal Material

*Perenniporia centrali-africana* was collected by C. Decock and J. C. Matasyoh from wood from Mount Elgon National Reserve, located in the western part of Kenya (1°7′6″ N, 34°31′30″ E) in April 2016. A dried specimen and the corresponding mycelial culture, which was obtained from the context of the basidome, were deposited at MUCL, Louvain-la-Neuve, Belgium, under the accession number MUCL 56028.

*Cerrena* sp. nov. was collected from an unnamed rotting tree trunk, isolated, and identified by T. Boonpratang, R. Choeyklin, and the team from Dong Yai Community Forest in the Plant Genetics Conservation Project, under the Royal Initiative of Her Royal Highness Maha Chakri Sirindhorn (RSPG), in Amnat Charoen Province, located in the northeastern part of Thailand, in March 2017. The voucher specimen collection was deposited in the BIOTEC Bangkok Herbarium & Fungarium, Pathum Thani, Thailand, with the designation BBH 41077, and the culture collection was deposited in the BIOTEC Culture Collection with the designation BCC 84628.

DNA was extracted from the cultures of MUCL 56028 and BBH 40848 using the EZ-10 spin column genomic DNA miniprep kit (Bio Basic Canada Inc., Markham, ON, Canada) as described previously [32]. A Precellys 24 homogenizer (Bertin Technologies, France) was used for cell disruption at a speed of 6000 rpm for 2 × 40 s. Standard primers ITS 1f and ITS 4r were used for the DNA region amplification by following a previously published protocol [33].

### 3.3. Seed Culture and Scale-Up of Fermentation

For the seed culture of *P. centrali-africana,* three 20 mm^2^-sized mycelium plugs of a well-grown culture on YM6.3 agar medium were transferred into a 500 mL Erlenmeyer flask, containing 200 mL of liquid YM6.3 medium. The incubation was performed on a rotary shaker at 23 °C and 140 rpm. After six days, the mycelium was homogenized with a T25 easy clean digital (S 25 N, IKA, Staufen im Breisgau, Germany) and afterwards used to inoculate twenty-five 500 mL Erlenmeyer flasks, each containing 200 mL of BAF medium. Therefore, 3 mL of the homogenized seed culture was transferred into each flask. The incubation was performed on a rotary shaker at 23 °C and 140 rpm. Every five days, the consumption of glucose was monitored regularly (using Medi-Test, Macherey Nagel, Düren, Germany).

For *Cerrena,* the fermentation was started by inoculating a plug of well-grown culture on YM agar, which was incubated at 23 ± 2 °C for up to 2 weeks until the colony covered the plate. A cork borer (diameter 6 mm) was used to plug the well-grown colony in five pieces, which were inoculated into 250 mL of liquid YM6.3 medium for 20 flasks in total, which were incubated on a rotary shaker at 27 ± 2 °C and 140 rpm. The glucose consumption was monitored every five days as mentioned above.

### 3.4. Harvest, Extraction, and Analytical HPLC

After 20 days of incubation, the liquid BAF cultures of *P. centrali-africana* were harvested. The mycelium and supernatant were separated by centrifugation (Sorvall RC-58 Refrigerated Superspeed Centrifuge, DuPont Instruments) at 9000 rpm for 30 min. The mycelium was extracted twice with acetone in an ultrasonic bath. The acetone and mycelium were separated by filtration and subsequently the organic solvent was removed by evaporation (40 °C). Afterwards, the remaining aqueous phase was extracted with ethyl acetate 1:1, twice, and following this, the extract was evaporated to dryness. The supernatant was extracted with ethyl acetate 1:1, twice. After phase separation, the organic phase was collected in a round-bottom flask and the ethyl acetate was then evaporated (40 °C). Crude extracts from the mycelium and supernatant were dissolved in methanol to yield a concentration of 10 mg/mL. Solvation was aided by ultrasonication at 40 °C. Additionally, 100 µL of this solution was filtered with syringeless filters (Mini-UniPrep^TM^, Whatman, Dassel, Germany) and 60 µL of the supernatant was analyzed by analytical HPLC–MS.

The rice standing culture of *P. centrali-africana* was harvested after 28 days of incubation. Therefore, the medium and mycelium were loosened with a spatula, covered with acetone, and finally extracted by using ultrasonification for 30 min. Afterwards, the liquid phase was separated from the solid phase by filtration to enable a second extraction of the solid phase with acetone. Following this, the organic solvent was evaporated using a rotary evaporator (40 °C) and the remaining aqueous phase was extracted with ethyl acetate (1:1) in a separatory funnel, twice. The crude extract was evaporated to dryness (40 °C) and subsequently dissolved in 5 mL of methanol. Into this solution, 50 mL of a mixture of heptane and MeOH/H_2_O (1:1) was added, while the following extraction was carried out in a separatory funnel, twice, and the heptane and aqueous phase were collected separately. Finally, both fractions were evaporated to dryness (40 °C) and again dissolved in methanol to yield a concentration of 10 mg/mL. Solvation was aided by ultrasonication at 40 °C. Additionally, 100 µL of this solution was filtered with syringeless filters (Mini-UniPrep^TM^, Whatman) and 60 µL of the supernatant was analyzed by analytical HPLC–MS.

After 35 days of cultivation, the mycelia and supernatant of the *Cerrena* cultures were separated by centrifugation. The mycelial biomass was extracted with 4 × 500 mL of acetone in an ultrasonic bath (Sonorex Digital 10 P, Bandelin Electronic GmH&Co.KG, Berlin, Germany) for 30 min. The extracts were combined, and the solvent was evaporated by means of a rotary evaporator. The remaining water phase was suspended in 200 mL of distilled water, extracted with 3 × 500 mL of ethyl acetate, and stirred together with anhydrous sodium sulfate for 15 min. The filtered ethyl acetate extract was evaporated to dryness, leaving a brown oily solid (662 mg). The supernatant was extracted by adding 5% Amberlite XAD-16N absorbent (Rohm & Haas Deutschland GmbH, Frankfurt am Main, Germany) and stirred overnight. The Amberlite resin was then filtered and eluted with 4 × 500 mL of acetone. The resulting acetone extract was evaporated, and the remaining water phase was extracted with ethyl acetate 1:1. The organic phase was dried over anhydrous sodium sulfate and evaporated to dryness, and a brown oily extract (3.2 g) was obtained.

### 3.5. Isolation of the Compounds

All separations were carried out as RP-LC at a PLC 2250 system (Gilson, Middleton, WI, USA), using water as solvent A and ACN as solvent B, both containing 0.1% of formic acid.

At first, the crude extracts of the liquid BAF cultures were dissolved in methanol and filtered through a Strata X-33 µm Polymere Reversed Phase Tube (Phenomenex, Aschaffenburg, Germany) to remove nonpolar compounds and debris. To obtain a further separation, the main fractions were separated with a Gemini C18 column (250 × 50 mm, 10 µm, Phenomenex) as stationary phase with a flow rate of 60 mL/min. UV detection was carried out at 210, 220, and 250 nm.

The mycelial extract (600 mg) was separated with an elution gradient starting with isocratic conditions for 5 min at 20% of solvent B, followed by an increase to 60% B over 40 min, another increase to 100% B over 10 min, and ultimately, isocratic conditions for 10 min at 100% B. According to the observed peaks, 28 fractions were collected. In the course of the following work, fraction 1 (5.03 mg, t_R_ = 5.5 min) was identified as 3β-Hydroxy-9-dehydroxy-pereniporin A (**1**), fraction 11 (20.28 mg, t_R_ = 20.5 min) as 3β-Hydroxy-6-O-acetylpereniporin A, fraction 13 (20.15 mg, t_R_ = 23.5 min) as sulphureine H, fraction 16 (60.56 mg, t_R_ = 27.5 min) as pereniporin A, fraction 17 and 18 (2.96 and 1.87 mg, t_R_ = 31.5–32.0 min) as 6α,9α,11α -trihydroxycinnamolide, fraction 27 (11.48 mg, t_R_ = 40.5–41.0 min) as 6-O-acetylpereniporin A, and fraction 28 (2.47 mg, t_R_ = 43.0–44.0 min) as 11α-Hydroxycinnamosmolide.

The extract obtained from the supernatant (720 mg, two runs) was separated by using the same gradient as described for the mycelial extract. After separation, 11 fractions were collected in the first run, whereof fraction 1 (15.51 mg, t_R_ = 20.5 min) was identified as 3β-hydroxy-6-O-acetylpereniporin A, fraction 3 (12.84 mg, t_R_ = 23.5 min) as sulphureine H, fraction 5 (46.26 mg, t_R_ = 27.5 min) as pereniporin A, fraction 6 (3.32 mg, t_R_ = 32.0 min) as 6α,9α,11α -trihydroxycinnamolide, and fraction 10 (2.61 mg, t_R_ = 44.0–44.5 min) as 11α-Hydroxycinnamosmolide. The second run led to the isolation of 19 fractions, leading to the identification of fraction 2 (5.33 mg, t_R_ = 10.3 min) as 3β-Hydroxy-9-dehydroxy-pereniporin A (**1**), fraction 10 (17.58 mg, t_R_ = 23.5 min) as sulphureine, and fraction 19 (1.27 mg, t_R_ = 43.0–43.3 min) as 11α-Hydroxycinnamosmolide.

A second cultivation in BAF medium under the same conditions led to another supernatant crude extract (252 mg) that was purified with a Gemini C18 column (250 × 50 mm, 10 µm, Phenomenex) as stationary phase, a flow rate of 60 mL/min, and UV detection at 210, 220, and 250 nm. An elution gradient starting with isocratic conditions for 2 min at 25% of solvent B, followed by an increase to 55% B over 40 min, another increase to 100% B over 15 min, and ultimately, isocratic conditions for 10 min at 100% B led to the isolation of 22 fractions. Fractions 4-6 (32.96 mg, t_R_ = 12.0–14.5 min) were found to include 6,7-Dehydro-isodrimenediol (**3**), while fraction 7 (33.27 mg, t_R_ = 16.0–17.5 min) was found to contain 6,7-Hydroxy-12-deoxy-dysidealactam (**2**).

Both were submitted to RP-LC for further purification. Fraction 7 containing 6,7-Hydroxy-12-deoxy-dysidealactam (**2**) was purified with a Luna C18 column (250 × 21.2 mm, 5 µm, Phenomenex) as stationary phase and flow rate of 15 mL/min. An elution gradient starting with isocratic conditions for 2 min at 20% of solvent B, followed by an increase to 35% B in 30 min and another increase to 100% B in 8 min led to the isolation of nine fractions, whereof fraction 7 (1.05 mg, t_R_ = 25.5 min) was identified as the only pure sample of compound **2**. Fractions containing 6,7-Dehydro-isodrimenediol (**3**) were combined and purified with a Luna C18 column (250 × 21.2 mm, 5 µm, Phenomenex) as stationary phase and a flow rate of 20 mL/min. An elution gradient starting with isocratic conditions for 5 min at 15% of solvent B, followed by an increase to 45% B over 40 min, another increase to 55% of solvent B over 10 min, and ultimately, an increase to 100% B over 10 min led to the isolation of seven fractions, whereof fraction 5 (0.94 mg, t_R_ = 40.0–41.5 min) was identified as the only pure sample of compound **3**.

Furthermore, the crude extract (140 mg) of the aqueous phase obtained from rice cultures was dissolved in methanol and separation was carried out using a Nucleodur C18 column (150 × 40 mm, 10 µm, Macherey-Nagel) as stationary phase, with UV detection at 210, 220, and 420 nm and a flow rate of 60 mL/min. For purification, an elution gradient starting with isocratic conditions for 5 min at 30% of solvent B, followed by an increase to 70% B over 40 min, another increase to 100% B over 20 min, and ultimately, isocratic conditions for 10 min at 100% B was used. A total of eleven fractions was collected, according to the observed peaks. Analysis via HPLC–MS and NMR led to the identification of fraction 1 (2.15 mg, t_R_ = 15.0–15.5 min) as pereniporin A, fraction 2 (2.15 mg, t_R_ = 19.0–19.5 min) as cryptoporic acid I, fraction 5 (6.48 mg, t_R_ = 34.5–35.0 min) as cryptoporic acid B, and fraction 6 (24.44 mg, t_R_ = 37.0–38.0 min) as cryptoporic acid H.

The isolation of compound **4-6** from *Cerrena* started with the filtration of the crude extracts using an SPME Strata-X 33 u Polymeric RP cartridge (Phenomenex, Inc., Aschaffenburg, Germany). The fractions from the mycelial crude extracts were separated using preparative reversed-phase liquid chromatography (PLC 2020, Gilson, Middleton, WI, USA). A VP Nucleodur 100−10 C18 ec column (250 mm × 40 mm, 7 μm, Macherey-Nagel) was used as stationary phase. Deionized water (Milli-Q, Millipore, Schwalbach, Germany) with 0.05% trifluoroacetic acid (TFA) (solvent A) and acetonitrile with 0.05% TFA (solvent B) were used as the mobile phase at a flow rate of 40 mL/min. The elution gradient started with 5% of solvent B isocratic for 3 min, followed by an increase to 50% of solvent B over 45 min, another increase to 100% of solvent B over 10 min and thereafter isocratic conditions at 100% of solvent B for 10 min. UV monitoring was carried out at 210, 254, and 350 nm. Fourteen fractions were collected according to the observed peaks. At this step, isodrimeniol (**4**) (2.0 mg, t_R_ = 57 min) was obtained from fraction 12. Reversed-phase HPLC using a VP Nucleodur 100−5C 18-ec column (150 mm × 21 mm, 7 μm, Macherey-Nagel) as stationary phase, H_2_O + 0.05% TFA as solvent A and ACN + 0.05% TFA as solvent B, at a flow rate of 20 mL/min was used to further purify the obtained fractions. A gradient starting with isocratic conditions at 20% B for 10 min, increasing to 40% B over 30 min, followed by a gradient shift from 40% to 100% B over 10 min, and maintaining isocratic conditions at 100% B for 5 min led to the isolation of **5** (3.5 mg, t_R_ = 16.3 min) from fraction F1. Fraction F2 was purified with a gradient starting with isocratic conditions at 20% B for 10 min, increasing to 25% B over 10 min, followed by an increase to 35% solvent B over 40 min, an increase to 100% B over 10 min, and maintaining isocratic conditions at 100% B for 5 min. This led to the isolation of the 4-Aminobutyl derivative of ugandensolide (**6**) (1.5 mg, t_R_ = 45.4 min). Fraction F6 was purified with a gradient starting with isocratic conditions at 20% B for 10 min, increasing to 45% B over 10 min, an increase to 55% of solvent B over 40 min, followed by a gradient shift from 55% to 100% B over 10 min, and finally maintaining isocratic conditions at 100% B for 5 min to obtain cryptoporic acid H (2.4 mg, t_R_ = 31.9 min). All the obtained fractions were evaporated to dryness (40 °C) and dissolved in methanol to reach a concentration of 1 mg/mL. Afterwards, 60 µL of these solutions was transferred into 300 µL HPLC–MS vials and submitted for analytical HPLC–MS to evaluate the purity and determine the molecular weight.

*3β-Hydroxy-9-dehydroxy-pereniporin A* (**1**) white solid; ${\left[\alpha \right]}_{D}^{20}\;$= +3 (c = 0.1, MeOH); UV/Vis (MeOH) λ_max_ (logε) 202 (3.52) nm (c = 0.005, MeOH); ECD (c = 0.1, MeOH): λ_max_ (Δε) 208 (+1.1), 193 (−0.8) nm; NMR data (^1^H: 700 MHz, ^13^C NMR 175 MHz, MeOH-*d*_4_) see Table 1; ESIMS *m*/*z* 291.13 [M + Na]^+^, 267.09 [M − H]^−^; HRESIMS *m*/*z* 291.1565 [M + Na]^+^ (calcd. for C_15_H_24_NaO_4_, 291.1567); t_R_ = 2.3 min.

*6*,*7-Hydroxy-12-deoxy-dysidealactam* (**2**) white solid; ${\left[\alpha \right]}_{D}^{20}$ = +21 (c = 0.1, MeOH); UV/Vis (MeOH) λ_max_ (logε) 206 (3.72) nm (c = 0.005, MeOH); ECD (c = 0.02, MeOH): λ_max_ (Δε) 235 (−0.8), 211 (+2.4) nm; NMR data (^1^H: 700 MHz, ^13^C NMR 175 MHz, Acetone-*d*_6_) see Table 1; ESIMS *m*/*z* 266.10 [M + H]^+^, 265.01 [M − H]^−^; HRESIMS *m*/*z* 266.1750 [M + H]^+^ (calcd. for C_15_H_24_NaO_3_, 266.1751); t_R_ = 4.6 min.

*6*,*7-Dehydro-isodrimenediol* (**3**) white solid; ${\left[\alpha \right]}_{D}^{20}$ = +10 (c = 0.04, MeOH); UV/Vis (MeOH) λ_max_ (logε) 235 (3.70), 201 (3.50) nm (c = 0.0025, MeOH); ECD (c = 0.02, MeOH): λ_max_ (Δε) 234 (+1.3) nm; NMR data (^1^H: 700 MHz, ^13^C NMR 175 MHz, MeOH-*d_4_*) see Table 1; ESIMS *m*/*z* 219.09 [M − H_2_O + H]^+^; HRESIMS *m*/*z* 219.1738 [M − H_2_O + H]^+^ (calcd. for C_15_H_23_O, 219.1743); t_R_ = 6.1 min.

*Isodrimeniol* (**4**) yellow oil; ${\left[\alpha \right]}_{D}^{20}$ = −5 (c = 0.1, MeOH); UV/Vis (MeOH) λ_max_ (log ε) 201 (0.46) nm (c = 0.005, MeOH); ECD (C = 0.1, MeOH): λ_max_ (Δε) 190 (+0.9) nm; NMR data (^1^H: 500 MHz, ^13^C NMR 125 MHz, Acetone-*d*_6_) see Table 2; HRESIMS [M + H]^+^ *m*/*z* 219.1740, calcd. 219.1743 for C_15_H_23_O, [2M + H]^+^ *m*/*z* 437.3412, calcd. 437.3414 for C_30_H_45_O_2_, [2M + Na]^+^ *m*/*z* 581.2830, calcd. 581.2833 for C_30_H_42_N_2_NaO_8_, t_R_ = 7.09 min, data are in good agreement with the literature [22].

*Glycinyl derivative of deacetylugandensolide* (**5**) light yellow oil; ${\left[\alpha \right]}_{D}^{20}$ = +36 (c = 0.1, MeOH); UV/Vis (MeOH) λ_max_ (log ε) 212.5 (0.545) nm (c. 0.005, MeOH); ECD (c = 0.1, MeOH): λ_max_ (Δε) 210 (+5.2) nm; NMR data (^1^H: 500 MHz, ^13^C NMR 125 MHz, MeOH-*d_4_*) see Table 2; HRESIMS [M + H]^+^ *m*/*z* 352.2124, calcd. 352.2118 for C_19_H_30_NO_5_, [M + Na]^+^ *m*/*z* 374.1933, calcd. 374.1938 for C_19_H_29_NNaO_5_, [2M + H]^+^ *m*/*z* 725.3976, calcd. 725.4008 for C_40_H_57_N_2_O_10_, [2M + Na]^+^ *m*/*z* 747.3802, calcd. 747.3827 for C_40_H_56_N_2_NaO_10_, t_R_ = 6.04 min.

*4-Aminobutyl derivative of ugandensolide* (**6**) light yellow oil; ${\left[\alpha \right]}_{D}^{20}$ = +31 (c = 0.1, MeOH); UV/Vis (MeOH) λ_max_ (log ε) 211.5 (0.681) nm (c = 0.005, MeOH); ECD (c = 0.1, MeOH): λ_max_ (Δε) 205 (+4.9) nm; NMR data (^1^H: 500 MHz, ^13^C NMR 125 MHz, Acetone-*d*_6_) see Table 2; HRESIMS [M + H]^+^ *m*/*z* 366.1915, calcd. 366.1911 for C_19_H_28_NO_6_, [M + Na]^+^ *m*/*z* 388.1727, calcd. 388.1731 for C_19_H_27_NNaO_6_, [2M + H]^+^ *m*/*z* 731.3747, calcd. 731.3750 for C_38_H_55_N_2_O_12_, [2M + Na]^+^ *m*/*z* 755.3627, calcd. 755.3608 for C_52_H_48_N_2_NaO_2_, t_R_ = 7.64 min.

### 3.6. Cytotoxicity Assay

In vitro cytotoxicity (IC_50_) was determined as previously described [34]. In brief, the assay was performed against a mouse fibroblast cell line (L929) and HeLa (KB-3.1) to screen the cytotoxicity of all compounds. Compound **5** was tested against further cell lines including epidermoid carcinoma cells (A431), adenocarcinomic human alveolar basal epithelial cells (A549), breast cancer cells (MCF-7), prostate cancer cells (PC-3), and human ovary adenocarcinoma (SK-OV-3). All cell lines were purchased from the DSMZ collection (Braunschweig, Germany). Cell lines L929, A549, and KB-3.1 were cultured in Dulbecco’s modified Eagle’s medium (DMEM; Gibco; Thermo Fisher Scientific, Dreieich, Germany); MCF-7 and A431 cells were cultured in RPMI-1640 medium (Gibco) and PC-3 cells in F12K (Gibco) medium, all supplemented with 10% fetal bovine serum (FCS; Gibco) and incubated under 5% CO_2_ at 37 °C. The tested compound was dissolved in methanol to yield a concentration of 1 mg/mL. A 60 μL amount of 1:1 serial dilutions of the test compounds was added to 120 μL aliquots of a cell suspension (50/mL) in 96-well microplates. After 5 days of incubation, an MTT (3-(4,5-dimethylthiazol-2-yl)-2,5-diphenyltetrazolium bromide) assay was performed. IC_50_ was reported at the concentration of the compounds that inhibited the growth of cells at 50% of the control. Methanol was used as a negative control.

### 3.7. Antimicrobial Assay

The minimum inhibitory concentrations (MICs) were determined according to previous reports [31]. Briefly, the MIC values were obtained using a serial dilution assay in 96-well microtiter plates. A stock solution of each compound was prepared (1 mg/mL in methanol) and 60 µL was pipetted into the first row (A) of the plate, containing 130 µL of a mixture of the test organisms (Table S2) and the culture medium. Next, 150 µL of the mixture mentioned above was pipetted to the first row and mixed gently, then 150 µL was transferred to the second row that already contained 150 µL of a mixture, and mixed by repeated pipetting before transferring to the next row until the last row (H) was reached. A 1:1 serial dilution was performed and the last 150 µL was discarded. Incubation was performed in a microplate vibrating shaker (Heidolph, Schwabach, Germany; model Ti-tramax 1000) at 200 rpm, with an incubated temperature of 30 °C for yeast and fungi and 37 °C for bacteria, for 24−48 h. The lowest concentration of the compounds preventing visible growth of the tested microorganisms was reported as the MIC. Tested concentrations ranged from 66.7 to 0.1 µg/mL.

## 4. Conclusions

Five new drimane sesquiterpenoids from cultures of two tropical basidiomycetes (i.e., *Perenniporia centrali-africana*, and *Cerrena* sp. nov.), denoted as one new derivative of pereniporin A, namely 3β-hydroxy-9-dehydroxy-pereniporin A, as well as three new drimane sesquiterpene lactams including 6,7-dihydroxy-12-deoxy-dysidealactam, glycinyl of deacetylugandensolide, and 4-aminobutyl derivative of ugandensolide, and 6,7-dehydro-isodrimenediol were isolated in this study. This is the first report of the isolation of sesquiterpene lactams from tropical Basidiomycota from two different countries (i.e., Kenya and Thailand). Although both basidiomycetes belong to different families, they produce similar types of compounds, some of which were never isolated previously. None of the sesquiterpene lactams showed biological activities in this study. Studies of the metabolites on other biological activities are presently underway.

## Figures and Tables

**Table 1:** NMR spectroscopic data (^13^C (δ_C_), 175 MHz and ^1^H (δ_H_), 700 MHz, methanol-*d*_4_) for 3β-hydroxy-9-dehydroxy-pereniporin A (**1**), 6,7-dihydroxy-12-deoxy-dysidealactam (**2**), and 6,7-dehydro-isodrimenediol (**3**).

Pos	1		2		3	
	δ_C, Type_	δ_H_ (*J* in HZ)	δ_C, Type_	δ_H_ (*J* in HZ)	δ_C, Type_	δ_H_ (*J* in HZ)
1	37.2, CH_2_	α: 1.44, m	37.7, CH_2_	α: 1.06, m	37.1, CH_2_	α: 1.40, m
		β: 1.70, m		β: 2.67, m		β: 1.85, dt (13.0, 3.4)
2	26.5, CH_2_	1.67, m	19.6, CH_2_	α: 1.46, m	28.3, CH_2_	1.70, m
				β: 1.80, m		
3	78.2, CH	α: 3.22, dd (11.4, 4.5)	44.3, CH_2_	α: 1.44, m	79.4, CH	α: 3.26, m
				β: 1.25, m		
4	39.0, C	-	34.2, C	-	39.8, C	-
5	57.0, CH	α: 1.31, d (8.9)	50.8, CH	α: 1.48, d (1.3)	56.2, CH	α: 1.98, br s
6	67.4, CH	β: 4.34, br d (8.9)	72.7, CH	α: 4.35, br s	133.3, CH	6.20, dd (10.1, 3.1)
7	120.3, CH	5.53, m	70.9, CH	α: 4.10, br s	129.3, CH	5.74, br d (10.1)
8	139.1, C	-	149.1, C	-	146.6, C	-
9	60.6, CH	α: 2.25, m	143.0, C	-	56.9, CH	α: 2.09, br d (4.7)
10	38.1, C	-	36.7, C	-	38.8, C	-
11	98.7, CH	β: 5.17, d (4.5)	173.4, C	-	60.6, CH_2_	α: 3.72, dd (10.8, 7.3)
						β: 3.924, dd (10.8, 7.3)
12	67.7, CH_2_	α: 4.46, m	46.7, CH_2_	α: 3.73, dd (18.7, 2.6)	112.6, CH_2_	4.96, s
		β: 4.18, m		β: 4.00, m		5.15, s
13	15.1, CH_3_	0.99, s	24.1, CH_3_	1.26, s	16.6, CH_3_	0.81, s
14	29.5, CH_3_	1.23, s	34.1, CH_3_	1.00, s	28.5, CH_3_	1.06, s
15	14.3, CH_3_	0.85, s	21.3, CH_3_	1.52, s	15.2, CH_3_	0.74, s

**Table 2:** NMR spectroscopic data (^13^C (δ_C_), 125 MHz and ^1^H (δ_H_), 500 MHz) for isodrimeniol (**4**, acetone-*d_6_*), the glycinyl derivative of deacetylugandensolide (**5**, methanol-*d_4_*), and the 4-aminobutyl derivative of ugandensolide (**6**, acetone-*d*_6_).

Pos	4		5		6	
	δ_C, Type_	δ_H_ (*J* in HZ)	δ_C, Type_	δ_H_ (*J* in HZ)	δ_C, Type_	δ_H_ (*J* in HZ)
1	40.0, CH_2_	α: 1.24, m	38.2, CH_2_	α: 2.56, br d (12.7)	37.7, CH_2_	α: 2.68, br d (13.2)
		β: 1.80, dq (13.7, 2.7)		β: 1.13, m		β: 1.12, m
2	18.7, CH_2_	α: 1.62, qt (13.7, 3.5)	19.9, CH_2_	α: 1.85, m	19.4, CH_2_	α: 1.81, m
		β: 1.45, m		β: 1.52, m		β: 1.54, m
3	42.7, CH_2_	α: 1.25, m	44.6, CH_2_	α: 1.28, m	44.2, CH_2_	α: 1.29, m
		β: 1.45, m		β: 1.46, m		β: 1.49, m
4	33.0, C	-	34.6, C	-	34.1, C	-
5	50.4, CH	1.31, dd (11.6, 5.4)	51.2, CH	1.45, m	49.7, CH	1.73, d (1.0)
6	23.9, CH_2_	α: 2.14, m	72.5, CH	4.28, br s	73.8, CH	5.46, m
		β: 1.92, m				
7	116.2, CH	5.47, br s	70.7, CH	4.05, d (1.5)	67.1, CH	4.12, br d (1.7)
8	138.2, C	-	147.6, C	-	147.0, C	-
9	62.0, CH	2.15, m	144.2, C	-	142.5, C	-
10	33.7, C	-	37.3, C	-	36.8, C	-
11	99.2, CH	5.19, dd (4.5, 4.0) 11-OH: 5.11, br d (4.5)	172.5, C	-	170.4, C	-
12	68.1, CH_2_	α: 4.33, br d (11.3)	52.3, CH_2_	α: 4.09, d (19.4)	51.6, CH_2_	α: 4.18, d (18.6)
		β: 4.04, m		β: 3.84, d (19.4)		β: 3.92, d (18.6)
13	21.3, CH_3_	0.93, s	24.1, CH_3_	1.24, s	23.5, CH_3_	1.04, s
14	33.0, CH_3_	0.88, s	34.2, CH_3_	1.03, s	33.7, CH_3_	1.01, s
15	13.8, CH_3_	0.81, s	21.3, CH_3_	1.49, s	21.3, CH_3_	1.50, s
1′	-	-	176.8, C	-	171.2, C	-
2′	-	-	32.3, CH_2_	2.31, t (7.4)	43.4, CH_2_	4.21, m
3′	-	-	25.2, CH_2_	1.88, quin (7.4)	-	-
4′	-	-	42.6, CH_2_	3.47, t (7.0)	-	-
1″	-	-	-	-	170.6, C	-
2″	-	-	-	-	21.5, CH_3_	2.01, s

## Data Availability

The data are available in the Supporting Information of the article.

## Supplementary Materials

The following supporting information can be downloaded at: https://www.mdpi.com/article/10.3390/molecules27185968/s1, Figure S1: BLAST comparison, Figure S2: The structure of all known compounds in the study, Figures S3–S50: ESIMS, HR-ESIMS, 1D and 2D NMR spectra. Tables S1–S5: ITS sequence and results of biological assays. Reference [31] is cited in the Supplementary Materials.

## Sample Availability

Samples of the compounds are not available from the authors as the material has been used up for bioassays.
