# Supplementary material for: Drimane-Type Sesquiterpenoids Derived from the Tropical Basidiomycetes Perenniporia centrali-africana and Cerrena sp. nov
Source: Molecules. 2022 Sep 14;27(18):5968. doi: 10.3390/molecules27185968 (PMC9500820; doi:10.3390/molecules27185968)
Supplement: Supplementary file 1 [file molecules-27-05968-s001.zip › molecules-1924171-supplementary.pdf]

Supplementary Information for

Drimane-type sesquiterpenoids derived from the Tropical Basidiomycetes *Perenniporia centrali-africana* and *Cerrena* sp. nov.

Paomephan Pathompong[1,2,†], Sebastian Pfütze[2,3,†], Frank Surup[2,3], Thitiya Boonpratang[4], Rattaket Choeyklin[4,5], Josphat C. Matasyoh[6], Cony Decock[6], Marc Stadler[2,3,*] and Chuenchit Boonchird[1*]

Contents

Table S1: ITS sequence of *Perenniporia centrali-africana* and *Cerrena* sp nov 4

Figure S1: Blast search of ITS sequence of *Perenniporia centrali-africana* 5

Figure S2: The structure of known compounds isolated in this study 6

Figure S3: HR-ESIMS data of 3β-Hydroxy-9-dehydroxy-pereniporin A (**1**)................ 7

Figure S4: ^{1}H NMR spectrum (700 MHz, methanol-d4) of 3β-Hydroxy-9-dehydroxy-pereniporin A (**1**)……………………………………………………………………....…8

Figure S5: ^{13}C NMR spectrum (700 MHz, methanol-d_4) of 3β-Hydroxy-9-dehydroxy-pereniporin A (**1**). 9

Figure S6: COSY NMR spectrum (500 MHz, methanol-d_4) of 3β-Hydroxy-9-dehydroxy-pereniporin A (**1**).. 10

Figure S7: HSQC NMR spectrum (700 MHz, methanol-d_4) of 3β-Hydroxy-9-dehydroxy-pereniporin A (**1**).. 11

Figure S8: HMBC NMR spectrum (700 MHz, methanol-d_4) of 3β-Hydroxy-9-dehydroxy-pereniporin A (**1**).. 12

Figure S9: ROESY NMR spectrum (700 MHz, methanol-*d*4) of 3β-Hydroxy-9-dehydroxy-pereniporin A (**1**). 13

Figure S16: HR-ESIMS data of 6,7-Dihydroxy-12-deoxy-dysidealactam (**2**). 14

Figure S17: ^{1}H NMR spectrum (700 MHz, acetone-*d*6) of 6,7-Dihydroxy-12-deoxy-dysidealactam (**2**). 15

Figure S18: ^{13}C NMR spectrum (700 MHz, acetone-*d*6) of 6,7-Dihydroxy-12-deoxy-dysidealactam (**2**). 16

Figure S19: COSY NMR spectrum (700 MHz, acetone-*d*6) of 6,7-Dihydroxy-12-deoxy-dysidealactam (**2**). 17

Figure S20: HSQC NMR spectrum (700 MHz, acetone-*d*6) of 6,7-Dihydroxy-12-deoxy-dysidealactam (**2**). 18

Figure S21: HMBC NMR spectrum (700 MHz, acetone-*d*6) of 6,7-Dihydroxy-12-deoxy-dysidealactam (**2**). 19

Figure S22: ROESY NMR spectrum (700 MHz, acetone-*d*6) of 6,7-Dihydroxy-12-deoxy-dysidealactam (**2**).................. 27

Figure S23: HR-ESIMS data of 6,7-Dehydro-isodrimenediol (**3**). 28

Figure S24: ^{1}H NMR spectrum (500 MHz, methanol-*d*4) of 6,7-Dehydro-isodrimenediol (**3**). 29

Figure S25: ^{13}C NMR spectrum (700 MHz, methanol-*d*4) of 6,7-Dehydro-isodrimenediol (**3**)... 23

Figure S26: COSY NMR spectrum (500 MHz, methanol-*d*4) of 6,7-Dehydro-isodrimenediol (**3**). 24

Figure S27: HSQC NMR spectrum (500 MHz, methanol-*d*4) of 6,7-Dehydro-isodrimenediol (**3**)..................................... 25

Figure S28: HMBC NMR spectrum (500 MHz, methanol-*d*4) of 6,7-Dehydro-isodrimenediol (**3**).................................... 26

Figure S29: ROESY NMR spectrum (500 MHz, methanol-*d*4) of 6,7-Dehydro-isodrimenediol (**3**). 27

Figure S30: ECD spectra of **1**-**3** compared to Pereniporin A. 28

Figure S31: COSY (blue arrows), HMBC (green arrows) and ROESY (violet arrows) correlations indicating the structures of **4**– **6.** 29

Figure S32: HR-ESIMS data of Isodrimeniol (**4**). 30

Figure S33: ^{1}H NMR spectrum (500 MHz) of Isodrimeniol (4) in acetone-d6……………………………………………...31

Figure S34: ^{13}C NMR spectrum (125 MHz) of Isodrimeniol (**4**) in acetone-d6 32

Figure S35: COSY NMR spectrum (500 MHz) of Isodrimeniol (**4**) in acetone-d6……………………………………….33

Figure S36: HSQC NMR spectrum (500 MHz) of Isodrimeniol (**4**) in acetone-d6……………………………………….34

Figure S37: HMBC NMR spectrum (500 MHz) of Isodrimeniol (**4**) in acetone-d6……………………………………...35

Figure S38: HR-ESIMS data of Glycinyl derivative of deacetylugandensolide (**5**)……………………………………..36

Figure S39: ^{1}H NMR spectrum (500 MHz) of Glycinyl derivative of deacetylugandensolide (**5**) in methanol-d4…….37

Figure S40: ^{13}C NMR spectrum (125 MHz) of Glycinyl derivative of deacetylugandensolide (5) in methanol-d4….....38

Figure S41: COSY NMR spectrum (500 MHz) of Glycinyl derivative of deacetylugandensolide (**5**) in methanol-d4…39

Figure S42: HSQC NMR spectrum (500 MHz) of Glycinyl derivative of deacetylugandensolide (**5**) in methanol-d4...40

Figure S43: HMBC NMR spectrum (500 MHz) of Glycinyl derivative of deacetylugandensolide (**5**) in methanol-d4..41

Figure S44: ROESY NMR spectrum (500 MHz) of Glycinyl derivative of deacetylugandensolide (**5**) in methanol-d4………...42

Figure S45: HR-ESIMS data of 4-Aminobutyl derivative of ugandensolide (**6**)…………………………………………43

Figure S46: ^{1}H NMR spectrum (500 MHz) of 4-Aminobutyl derivative of ugandensolide (**6**) in acetone-d6…………..44

Figure S47: ^{13}C NMR spectrum (125 MHz) of 4-Aminobutyl derivative of ugandensolide (**6**) in acetone-d6…………45

Figure S48: COSY NMR spectrum (500 MHz) of 4-Aminobutyl derivative of ugandensolide (**6**) in acetone-d6……...46

Figure S49: HSQC NMR spectrum (500 MHz) of 4-Aminobutyl derivative of ugandensolide (**6**) in acetone-d6……..47

Figure S50: HMBC NMR spectrum (500 MHz) of 4-Aminobutyl derivative of ugandensolide (**6**) in acetone-d6…….48

Table S2: Serial dilution assay experiment parameters…………………………..……………………………………49

Table S3: Cytotoxicity assay parameters ………………………………………………………….…………………51

Table S4: Antimicrobial activities of compounds 1-7………………………………………………………...…………52

Table S5: Cytotoxicity of compounds 1-7……………………………………………………………………………......53

Table S1: ITS sequence of *Perenniporia centrali-africana* and *Cerrena* sp nov

Strain	ITS sequence	Gene Bank number
Perenniporia centrali-africana	TAGAGGAAGTAAAAGTCGTAACAAGGTTTCCGTAGGTGAACCTGCGG<br>AAGGATCATTATCGAGTTTTGACTGGGTTAGAGCTGGCCTTCCGAGG<br>CATGTGCACGCCCTGCTCATCCACTCTACACCTGTGCACTTACTGTG<br>GGTTTCAGACGGTGTTAGCGGGCCTTTACCGGCTCGTGAAATCGTCT<br>GTGCCTGCGTTTATTACAAACACTTAAAAGTATCAGAATGTGTATTGCG<br>ATGTAACGCATCTATATACAACTTTCAGCAACGGATCTCTTGGCTCTC<br>GCATCGATGAAGAACGCAGCGAAATGCGATAAGTAATGTGAATTGCA<br>GAATTCAGTGAATCATCGAATCTTTGAACGCACCTTGCGCTCCTTGGT<br>ATTCCGAGGAGCATGCCTGTTTGAGTGTCATGAAATCTTCAACCTATAA<br>GCCTTTGCGGGTTTATTAGGCTTGGACTTGGAGGCTTGCCGGCMTAG<br>TCGTCGGCTCCTCTCAAATGCATTAGCTTGATTCCTTGCGGATCGGCT<br>CTCGGTGTGATAATTGTCTACGCCGCGACCGTGAAGCGTTTGGCGAG<br>CTTCTAACCGTCTCTTATGAGACAATCTATTGACCTCTGACCTCAAATC<br>AGGTAGGACTACCCGCTGAACTTAAGCATATCATA	KX584430
Cerrena sp. nov.	TAGAGGAAGT AAAAGTCGTA ACAAGGTTTC CGTAGGTGAA<br>CCTGCGGAAG GATCATTAAC GAGTTTTTGA CTTGGTTGTC<br>GCTGGCCTTA CGGGGCATGT GCACGCCTTG TTAATCCACT<br>CTACACCTGT GCACTTACTG TGGGTTTTCG AATTGTGAAT<br>CGAACCTTTG CGGGTTTGTG AAGCGTTCGG GCCTGCGTTT<br>ATTACAAACT ATAAAGTATT AGAATGTGTA TTGTGATGTA<br>ACGCATCTAT ATACAACTTT CAGCAACGGA TCTCTTGGCT<br>CTCGCATCGA TGAAGAACGC AGCGAAATGC GATAAGTAAT<br>GTGAATTGCA GAATTCAGTG AATCATCGAA TCTTTGAACG<br>CACCTTGCGC TCCTTGGTAT TCCGAGGAGC ATGCCTGTTT<br>GAGTGTCATG AAATCTTCAA CCTACAAACC TTTGCGGGTT<br>TATAGGATTG GACTTGGAGG CTTGTCGGTC TATTAGATCG<br>GCTTCTCTTA AATGCATTAG CTTGGTTCCT CGCGGATCGG<br>CTTACGGTGT GATAATGTCT ACGCCGCGAC CGTGAAGCGT<br>TTGGCGAGCT TCTAACAGTC TCGCTAGAGA CAACTTATTA<br>TGACCTCTGA CCTCAAATCA GGTAGGACTA CCCGCTGAAC<br>TTAAGCATAT CATA	MW512503

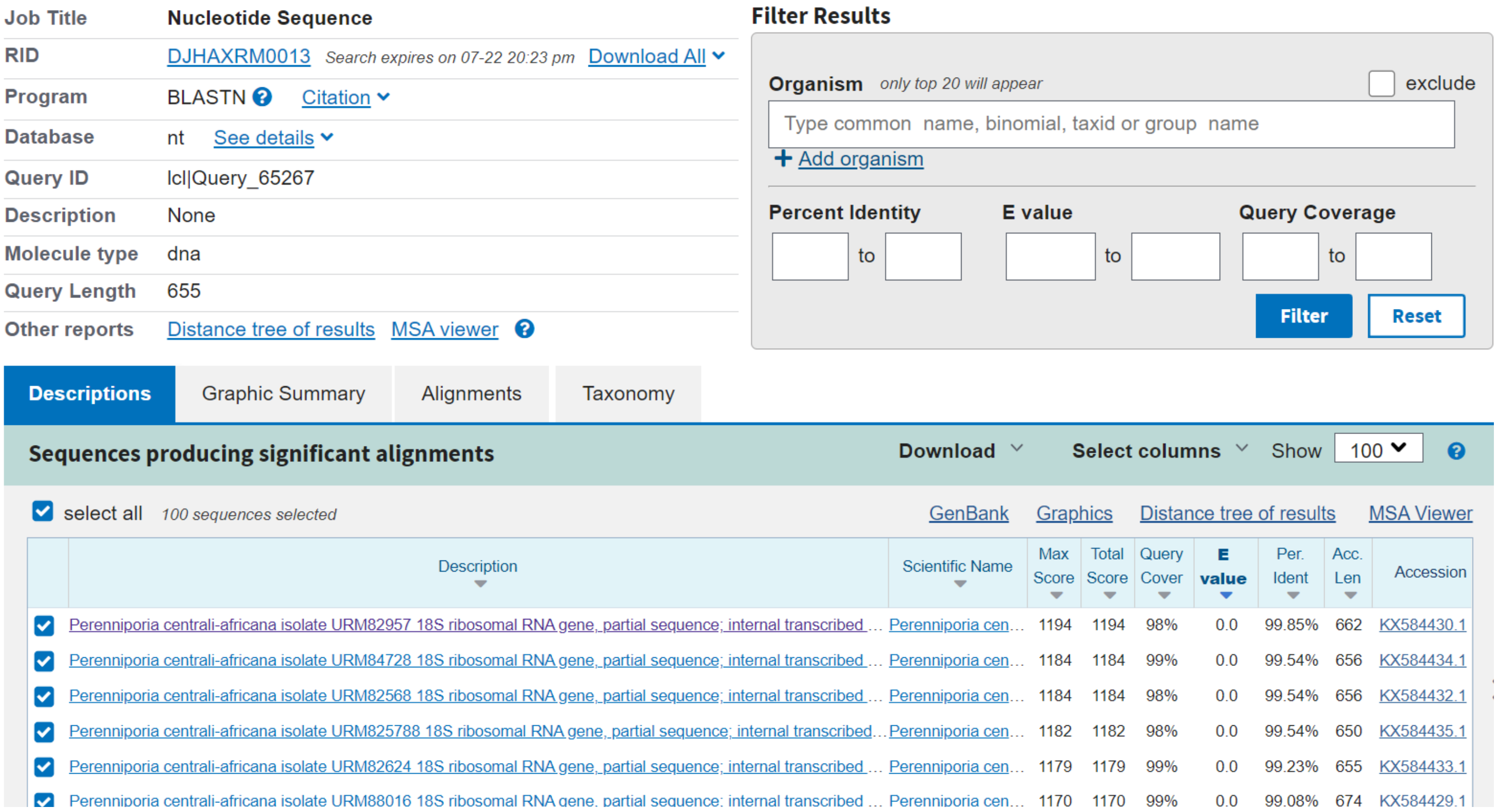

Figure S1: Blast search of ITS sequence of *Perenniporia centrali-africana*

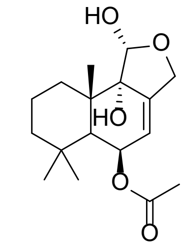

6-O-acetylpereniporin A (m/z=310)

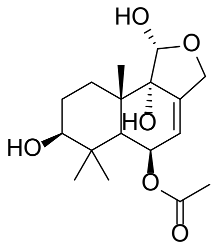

3β-Hydroxy-6-O-acetylpereniporin A (m/z=326)

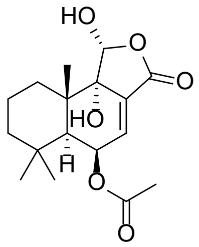

11α-Hydroxycinnamosmolide

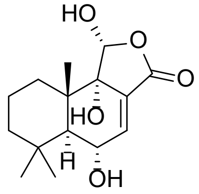

6α,9α,11α-trihydroxycinnamolide

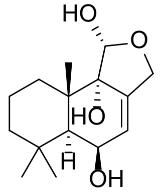

Pereniporin A

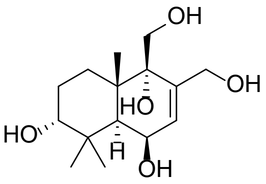

Sulphureuine H

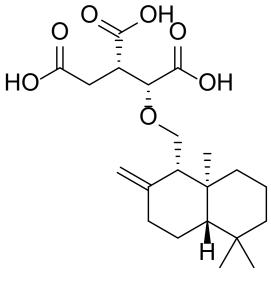

Cryptoporic acid H

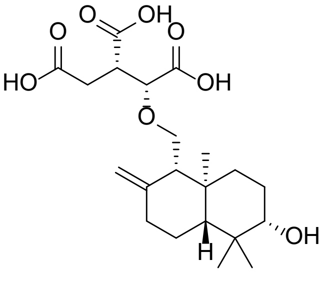

Cryptoporic acid I

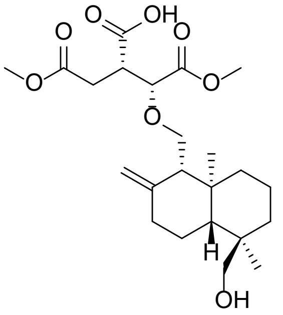

Cryptoporic acid B

Figure S2: The structure of known compounds isolated in this study

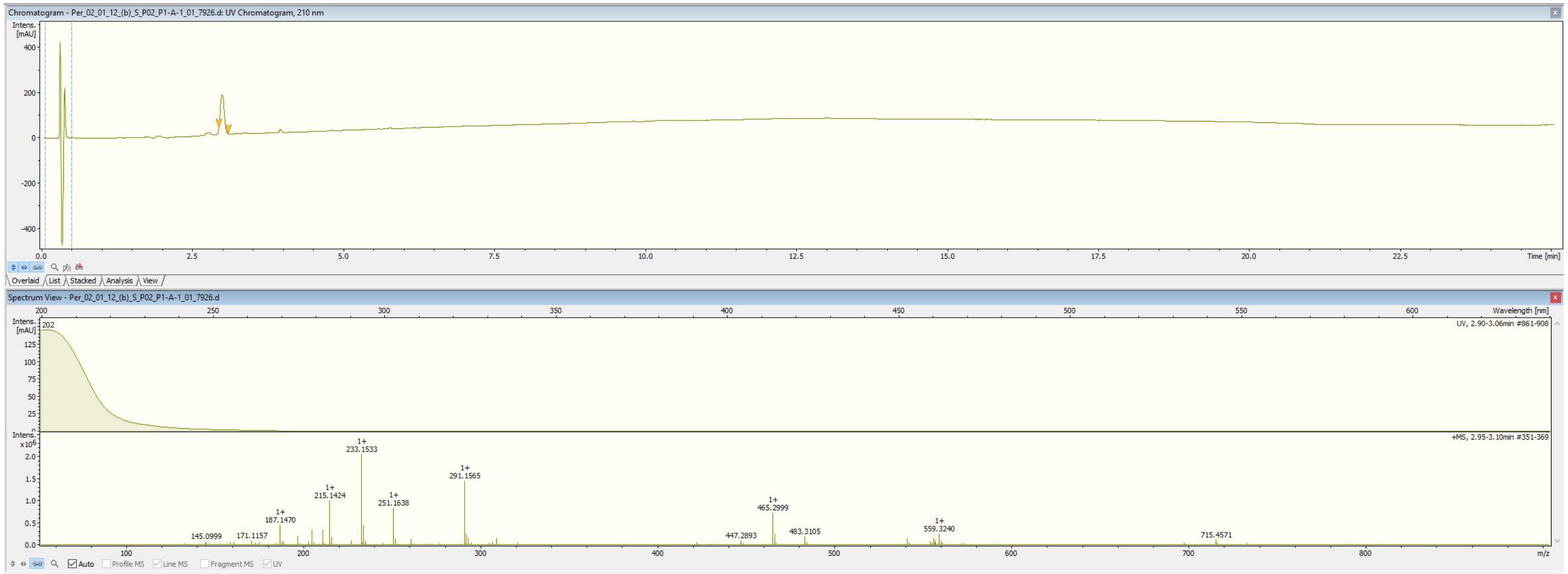

Figure S3: HR-ESIMS data of 3β-Hydroxy-9-dehydroxy-pereniporin A (**1**).

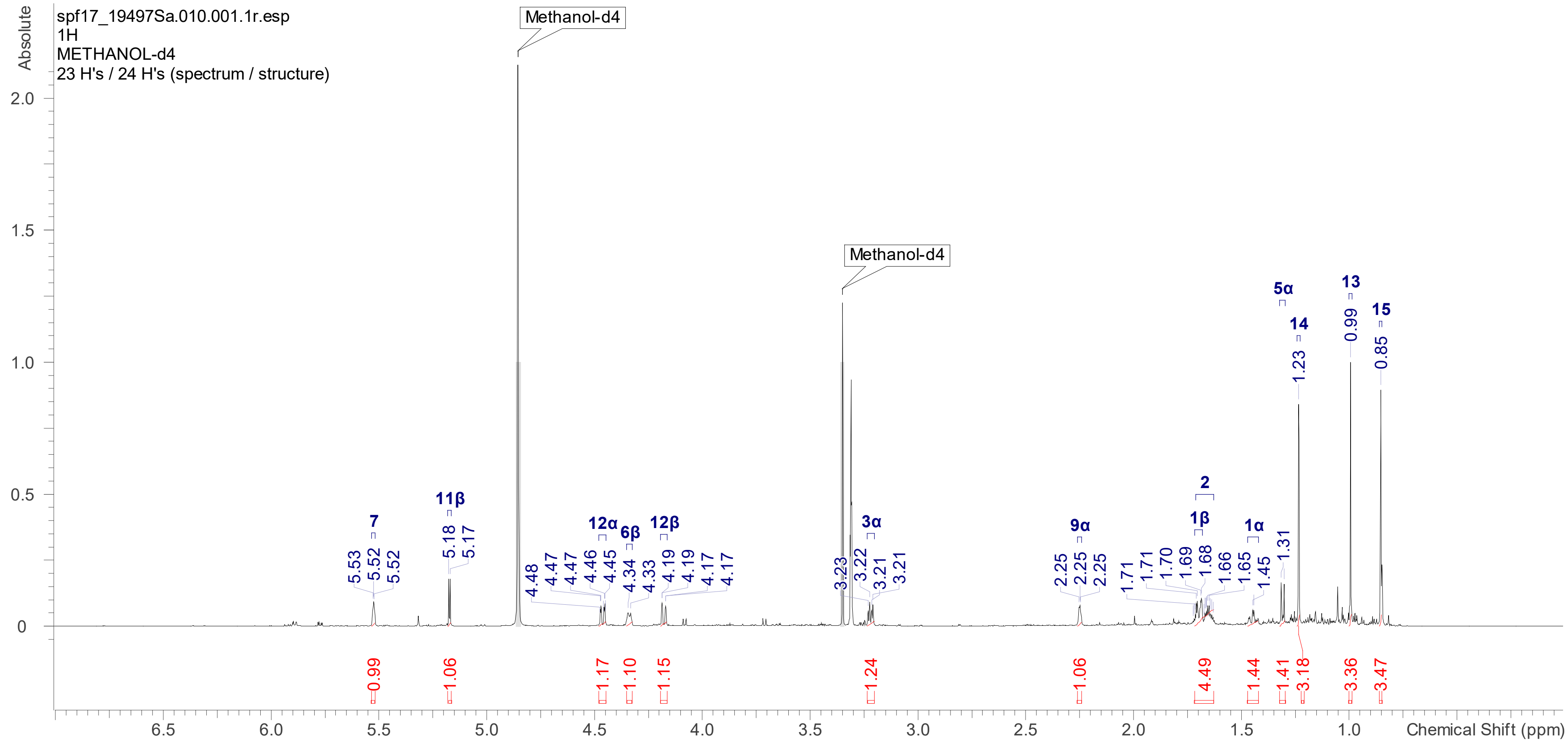

Figure S4: ^{1}H NMR spectrum (700 MHz, methanol-d4) of 3β-Hydroxy-9-dehydroxy-pereniporin A (**1**).

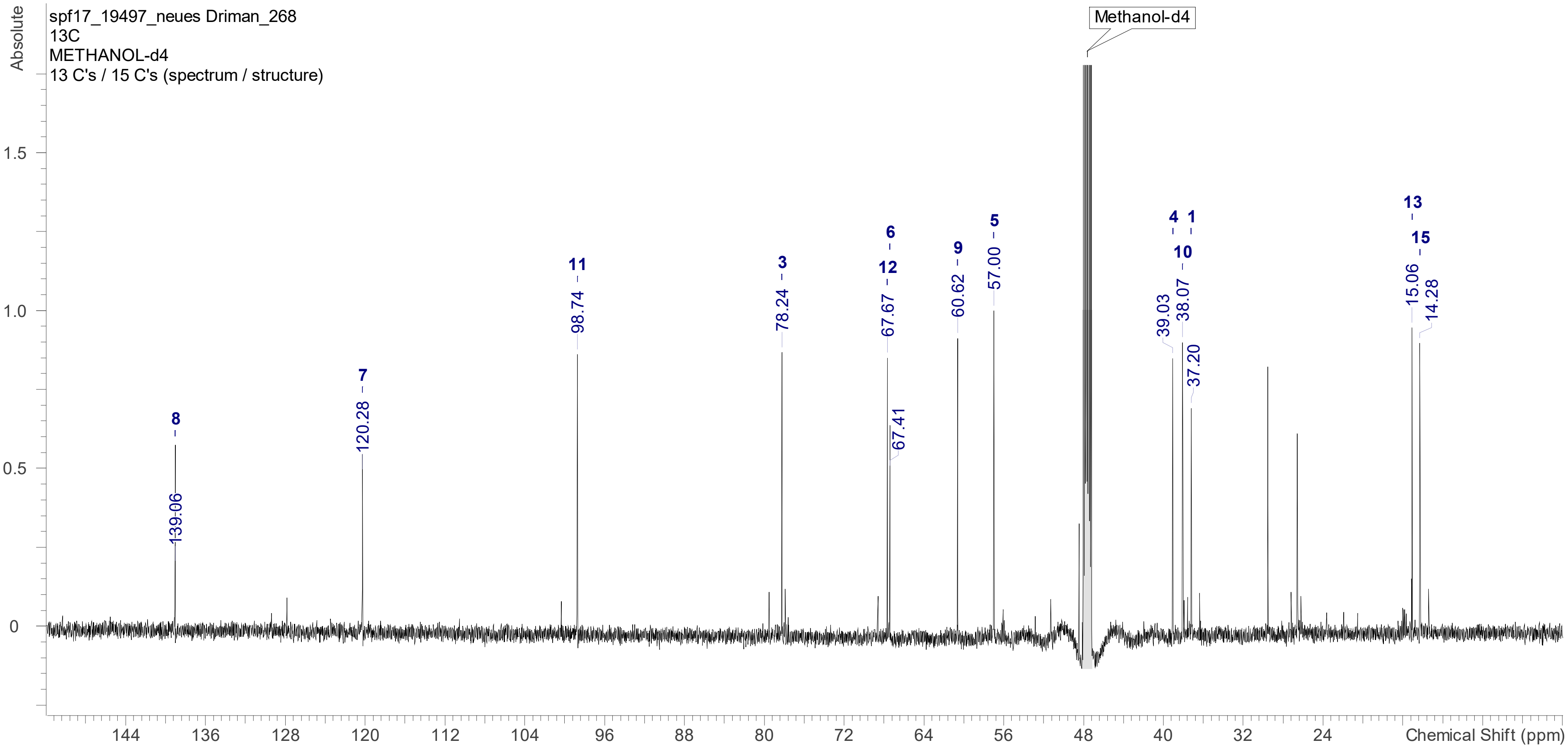

Figure S5: ^{13}C NMR spectrum (175 MHz, methanol-d_4) of 3β-Hydroxy-9-dehydroxy-pereniporin A (**1**).

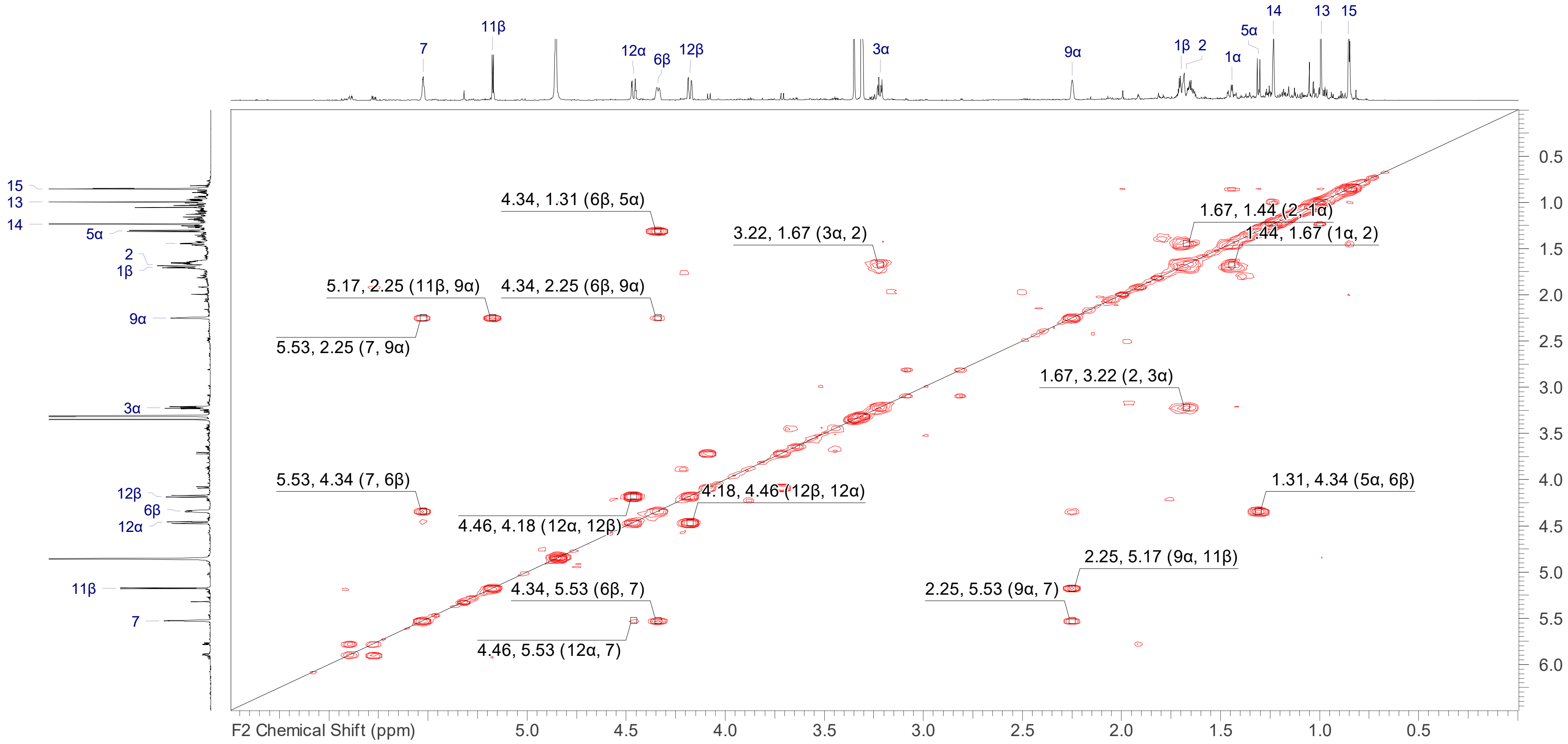

Figure S6: COSY NMR spectrum (500 MHz, methanol-d_4) of 3β-Hydroxy-9-dehydroxy-pereniporin A (**1**).

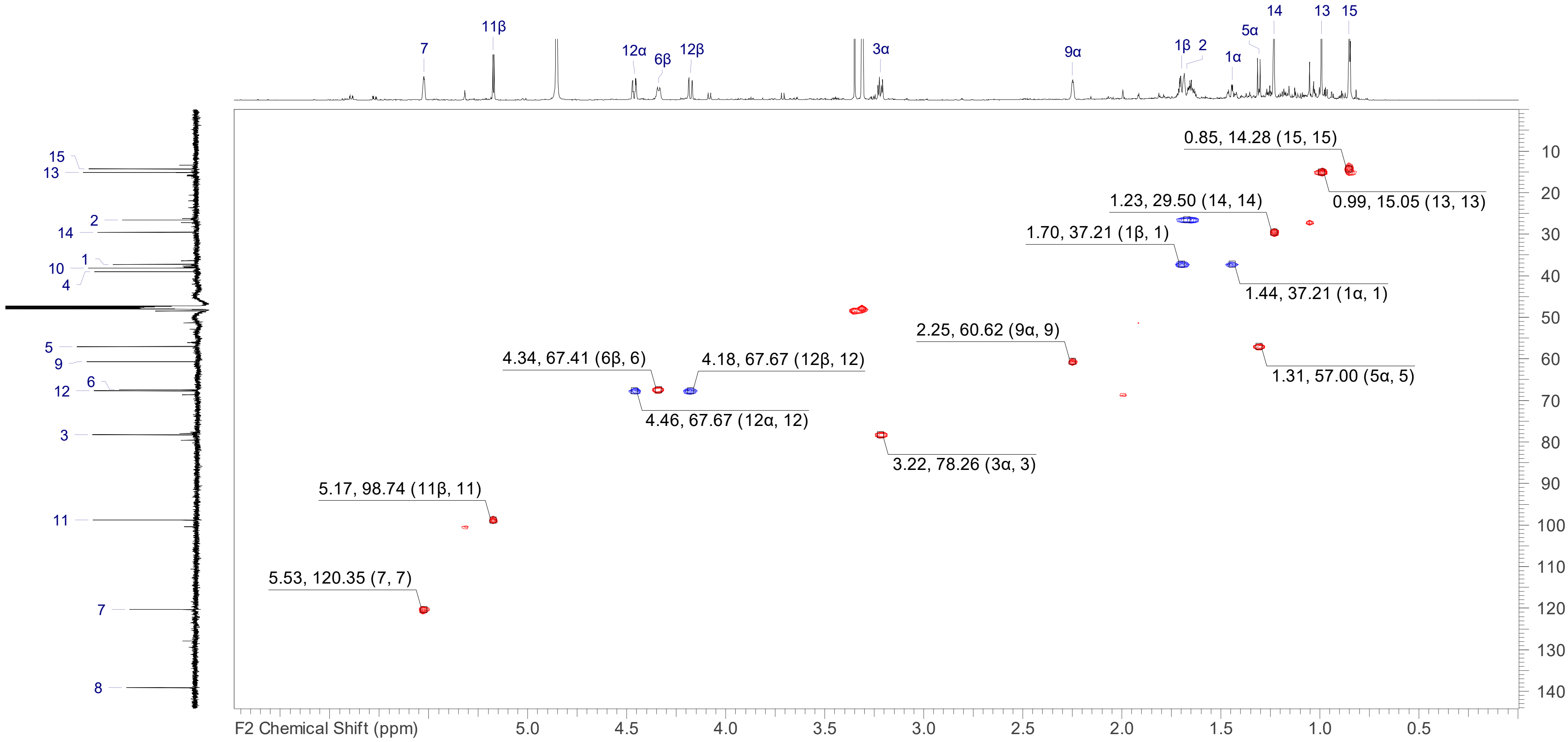

Figure S7: HSQC NMR spectrum (700 MHz, methanol-d_4) of 3β-Hydroxy-9-dehydroxy-pereniporin A (**1**).

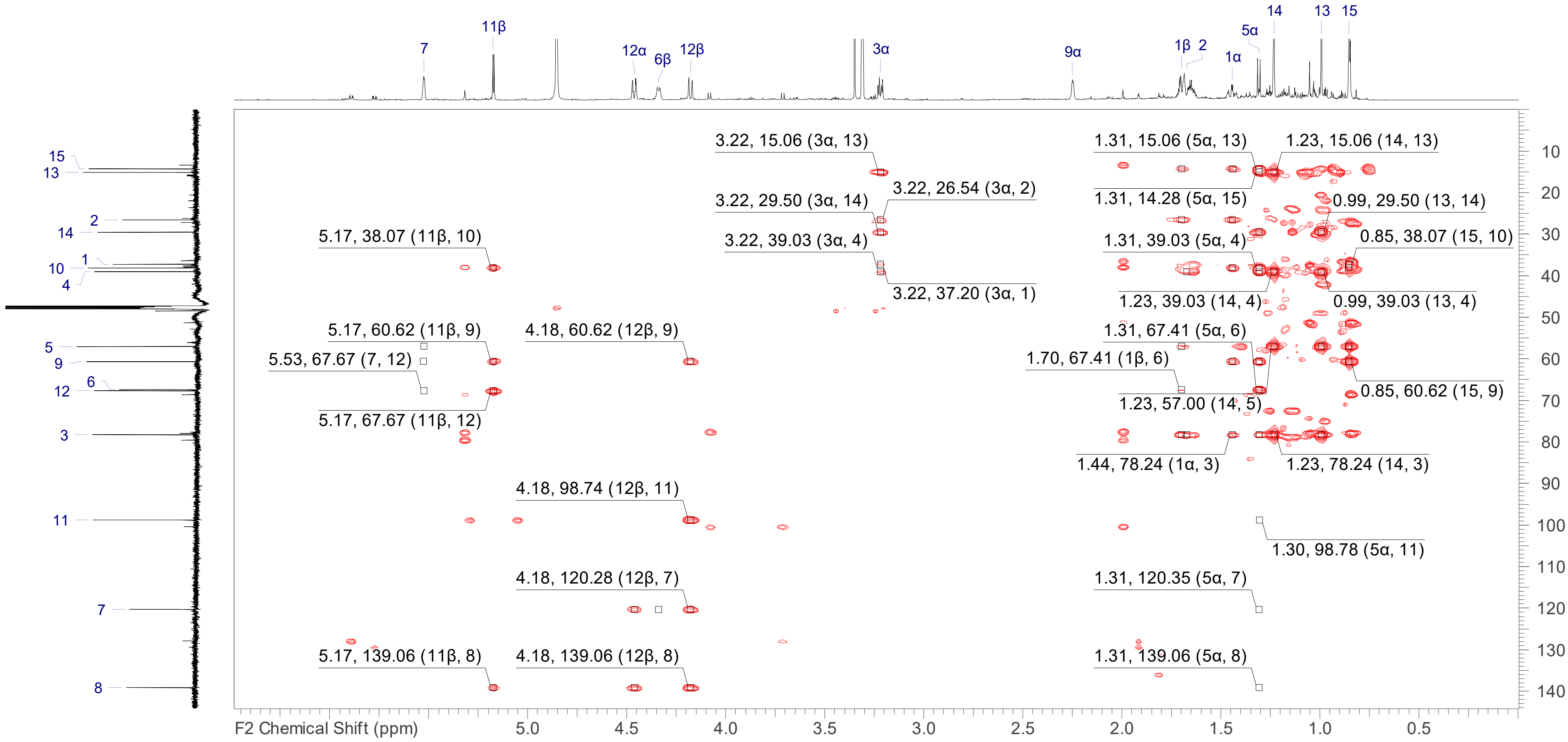

Figure S8: HMBC NMR spectrum (700 MHz, methanol-d_4) of 3β-Hydroxy-9-dehydroxy-pereniporin A (**1**).

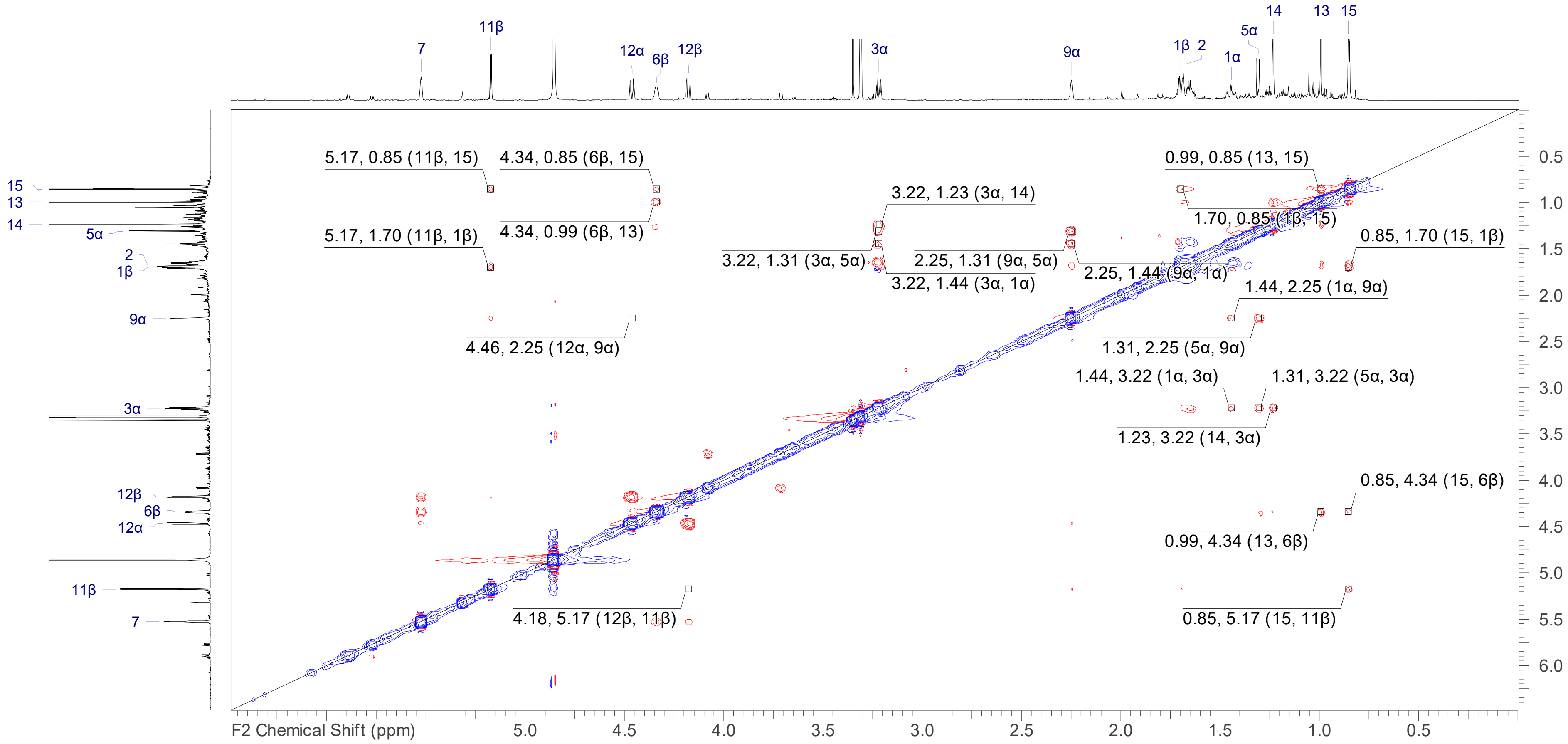

Figure S9: ROESY NMR spectrum (700 MHz, methanol-d_4) of 3β-Hydroxy-9-dehydroxy-pereniporin A (**1**).

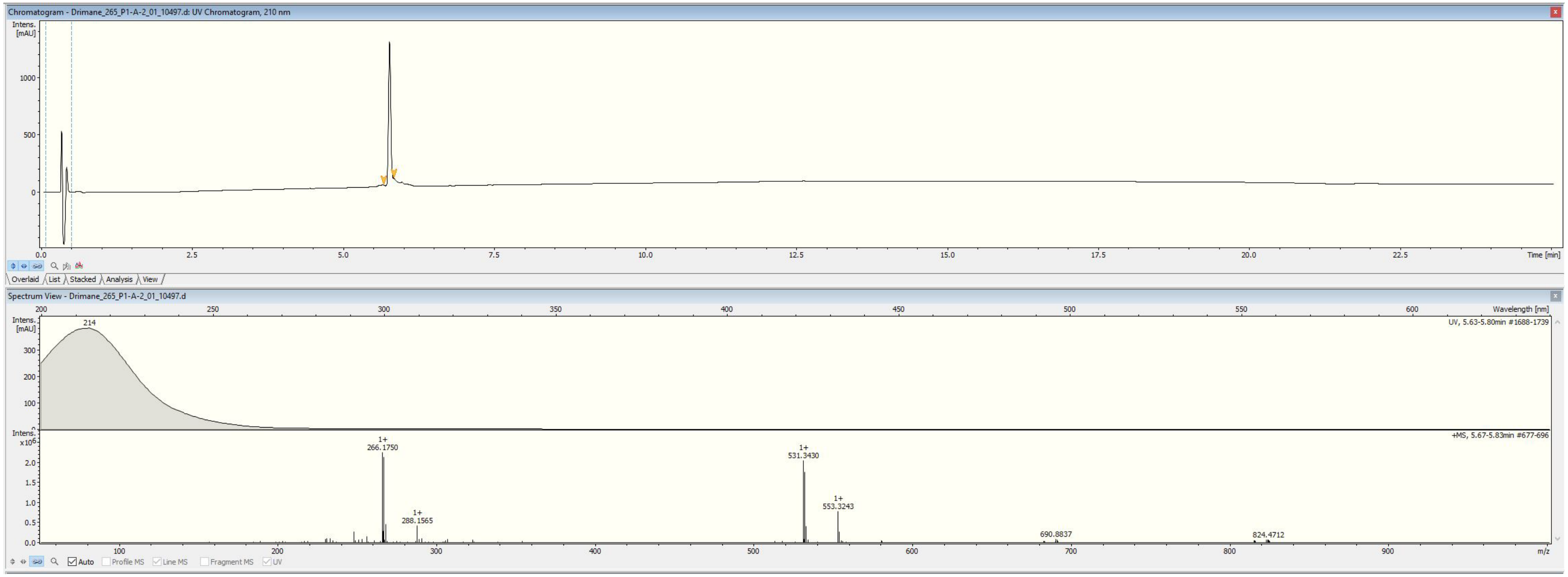

Figure S10: HR-ESIMS data of 6,7-Dihydroxy-12-deoxy-dysidealactam (**2**).

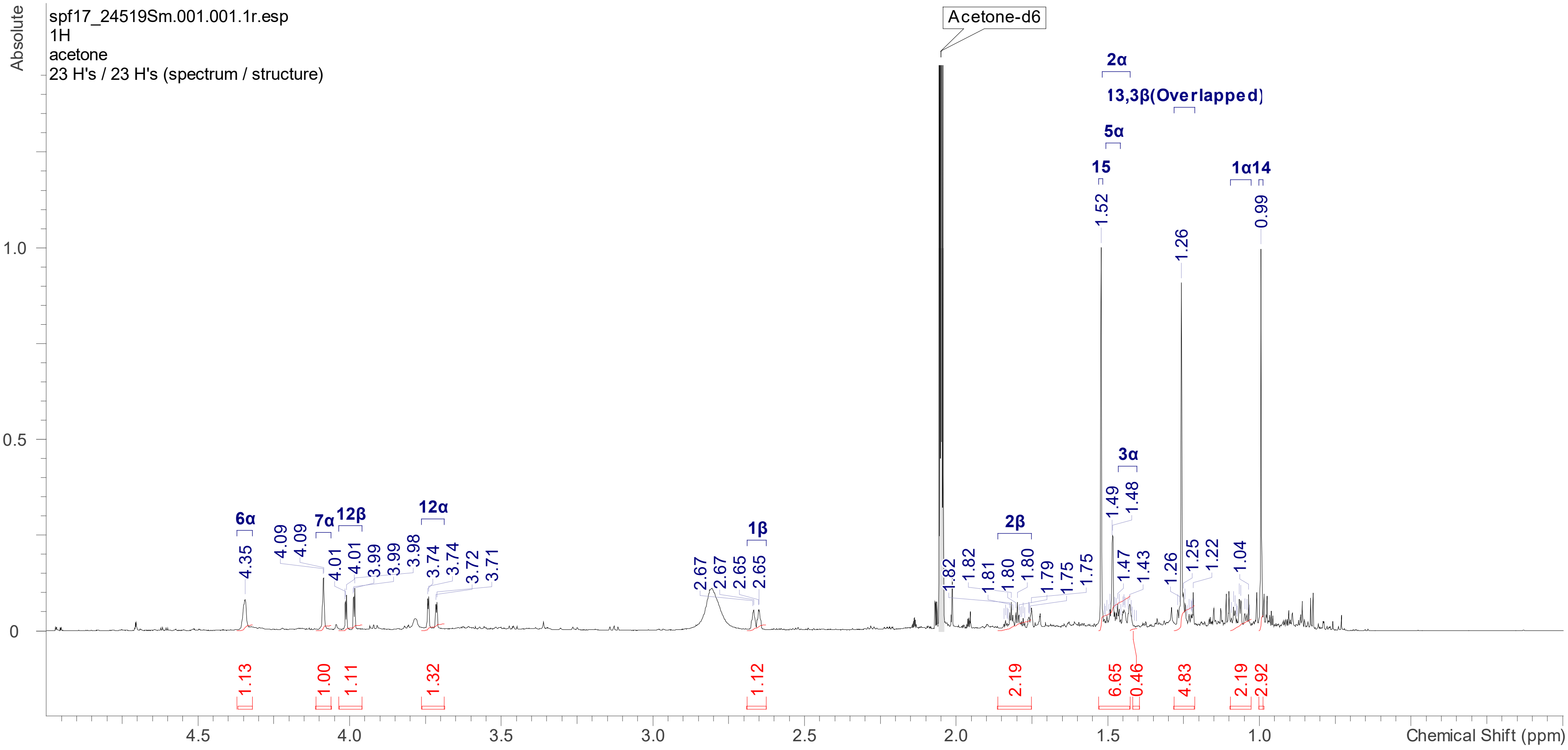

Figure S11: ^{1}H NMR spectrum (700 MHz, acetone-*d*6) of 6,7-Dihydroxy-12-deoxy-dysidealactam (**2**).

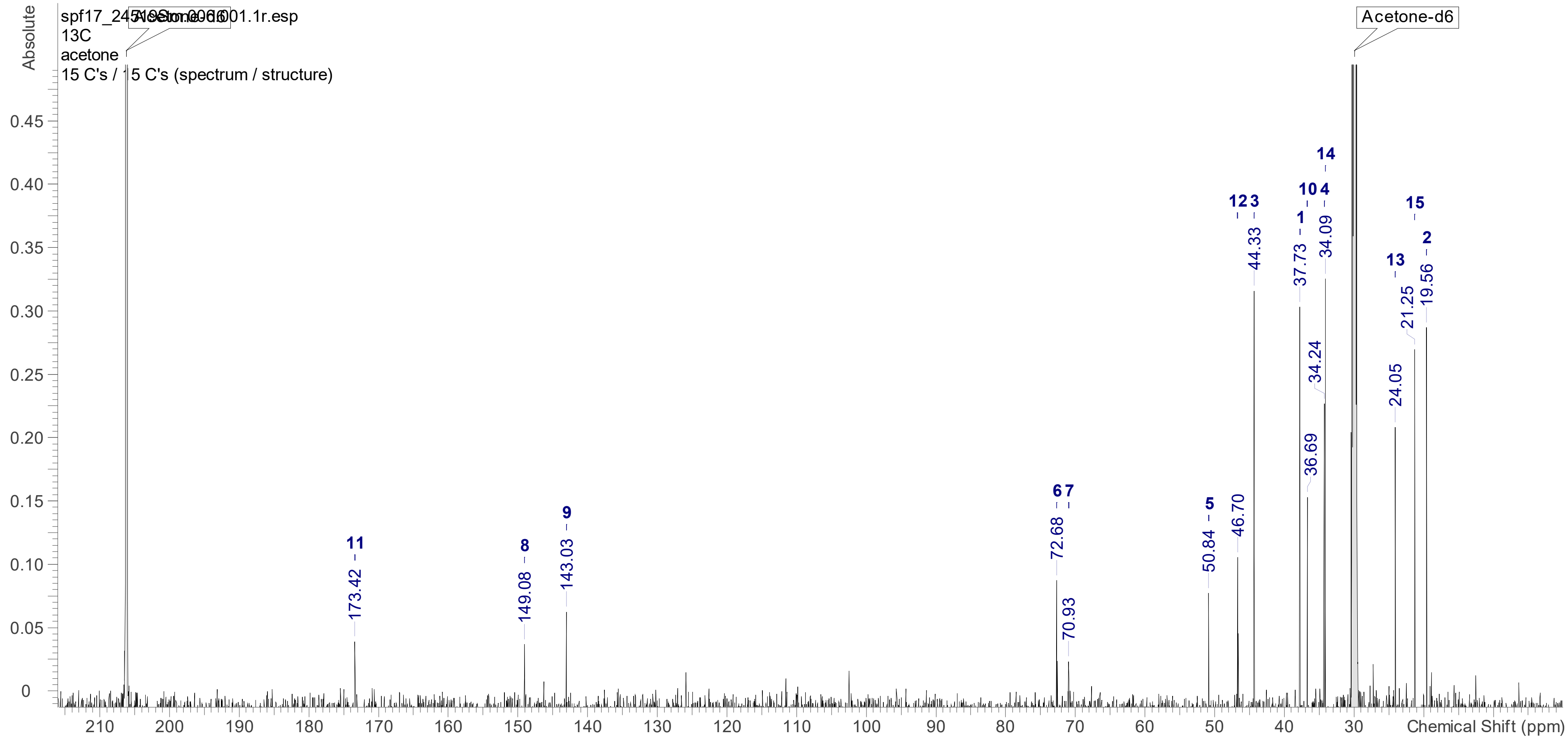

Figure S12: ^{13}C NMR spectrum (175 MHz, acetone-d_6) of 6,7-Dihydroxy-12-deoxy-dysidealactam (**2**).

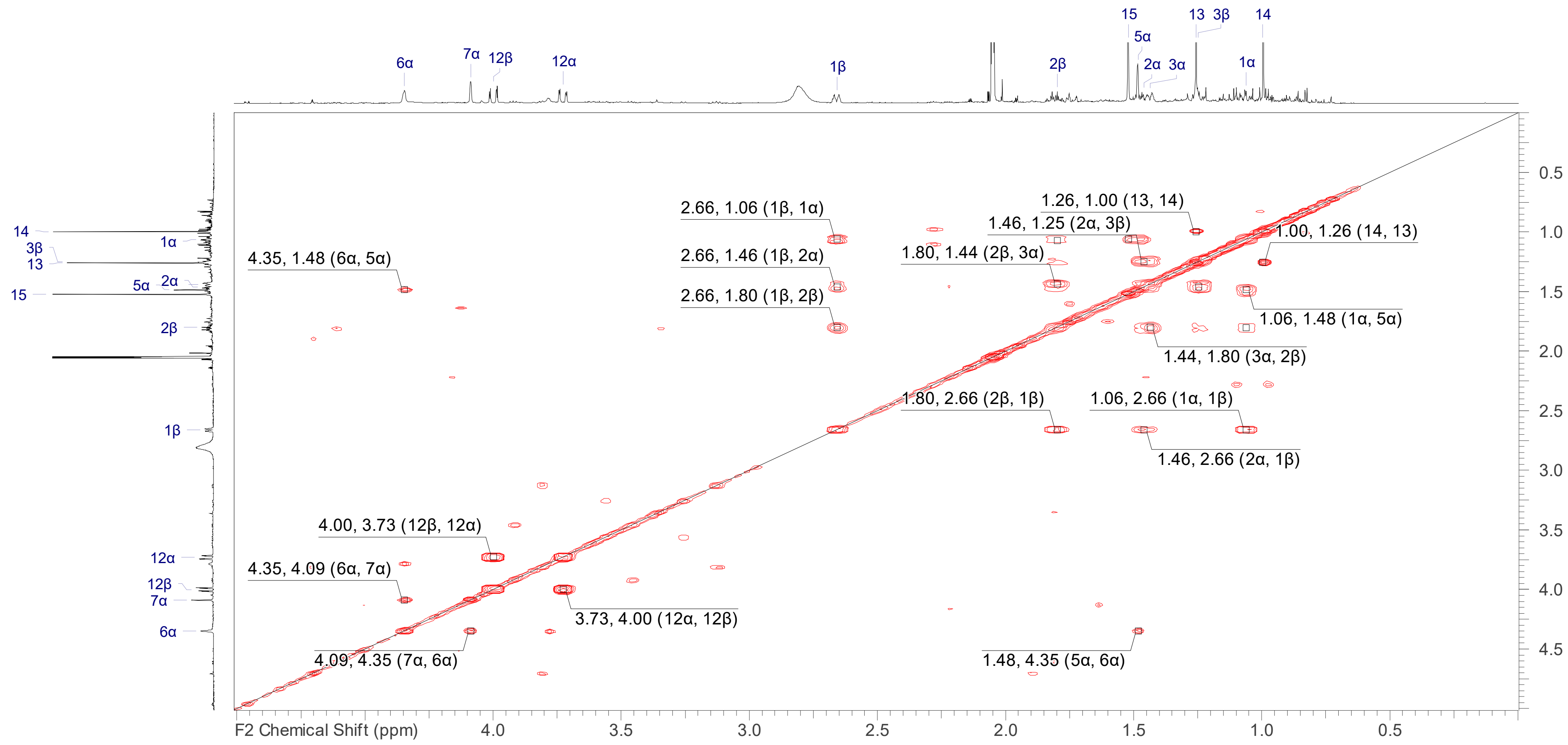

Figure S13: COSY NMR spectrum (700 MHz, acetone-d_6) of 6,7-Dihydroxy-12-deoxy-dysidealactam (**2**).

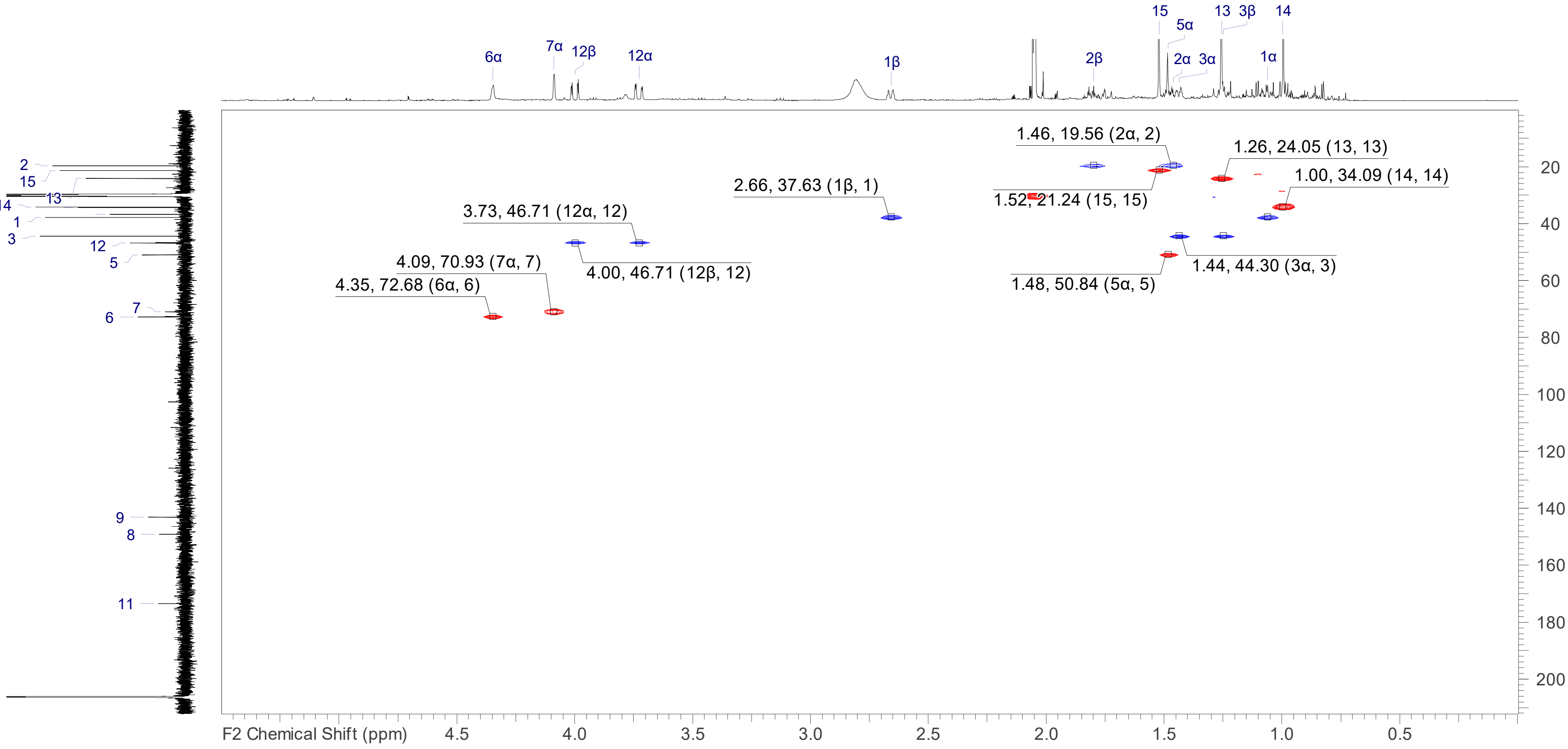

Figure S14: HSQC NMR spectrum (700 MHz, acetone-*d*6) of 6,7-Dihydroxy-12-deoxy-dysidealactam (**2**).

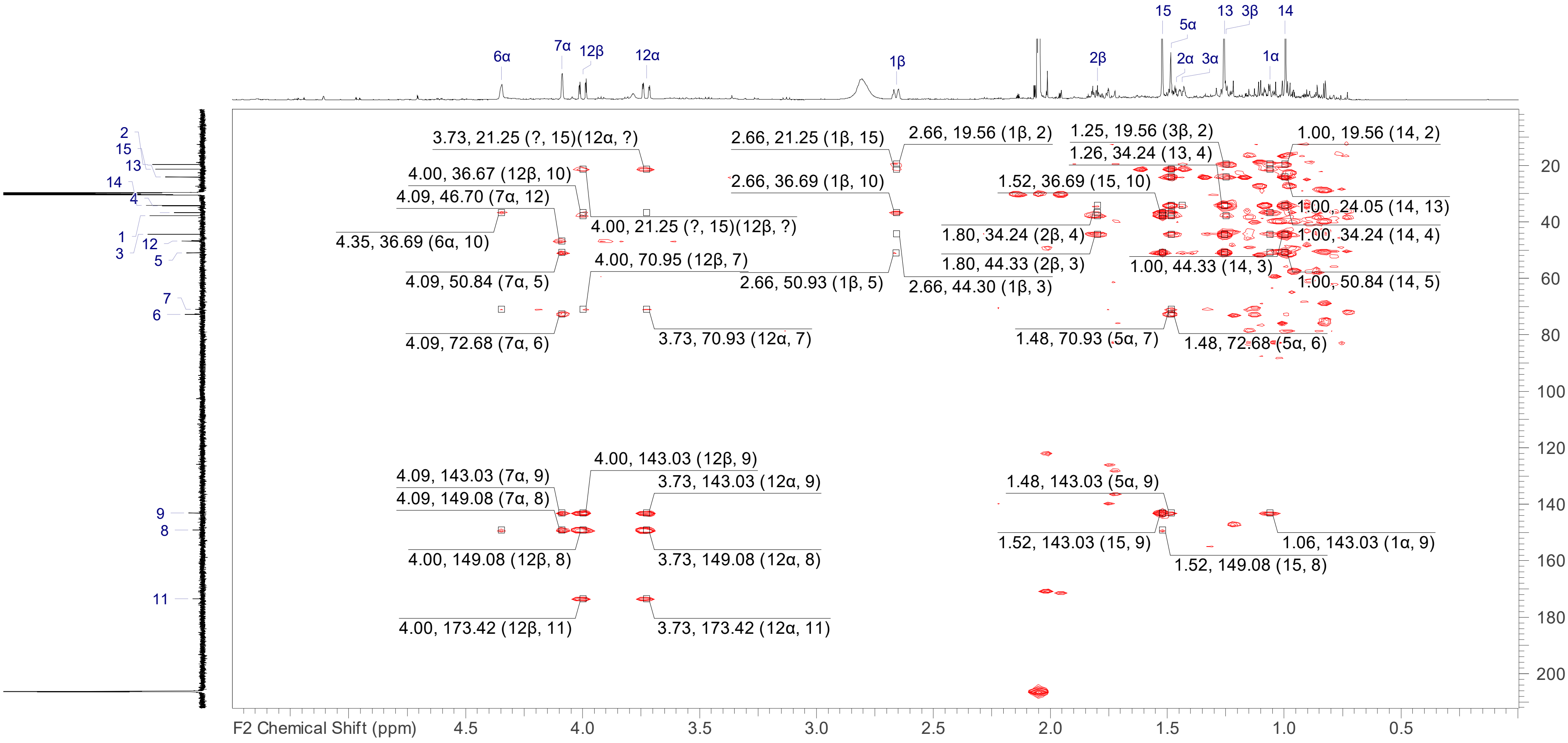

Figure S15: HMBC NMR spectrum (700 MHz, acetone-d_6) of 6,7-Dihydroxy-12-deoxy-dysidealactam (**2**).

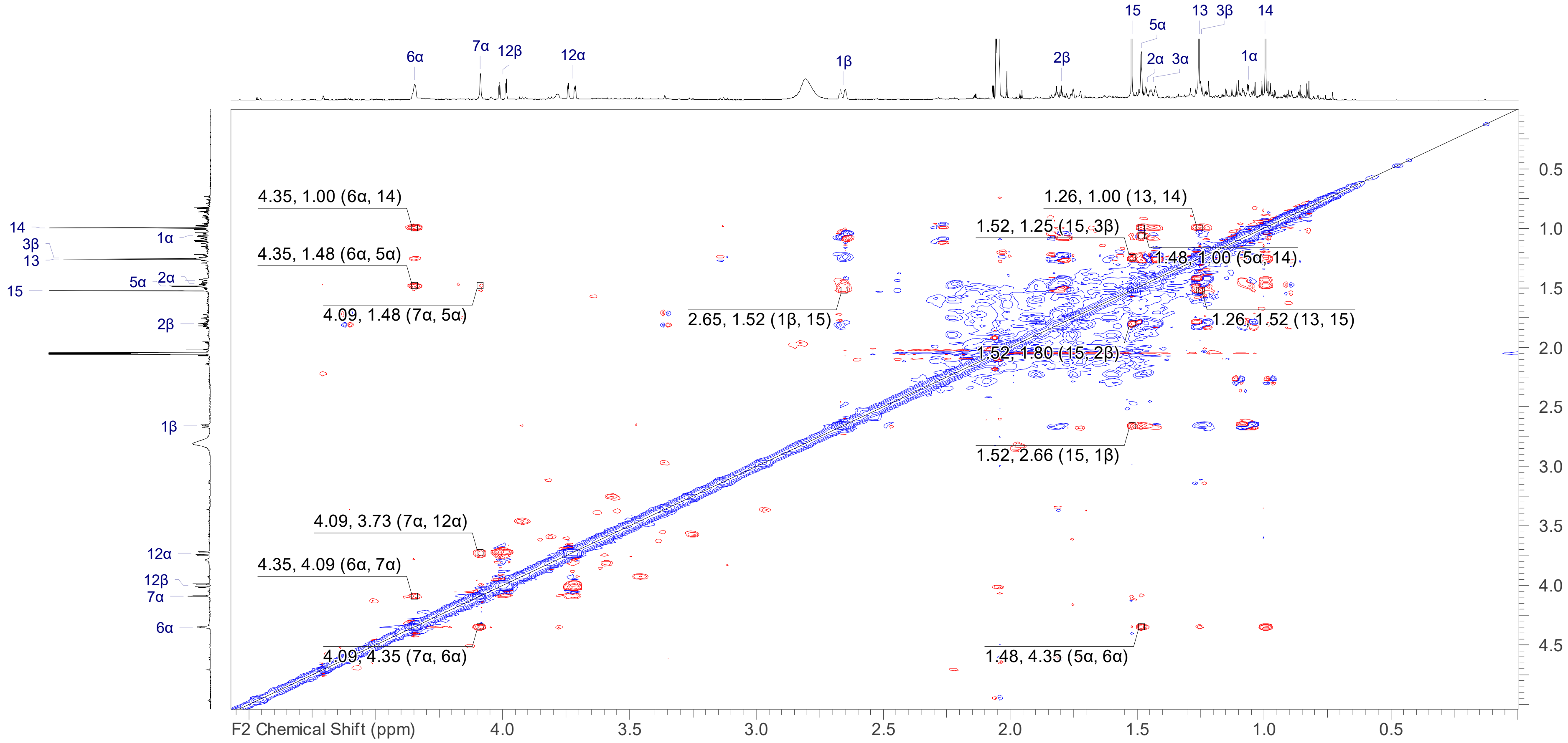

Figure S16: ROESY NMR spectrum (700 MHz, acetone-d_6) of 6,7-Dihydroxy-12-deoxy-dysidealactam (**2**).

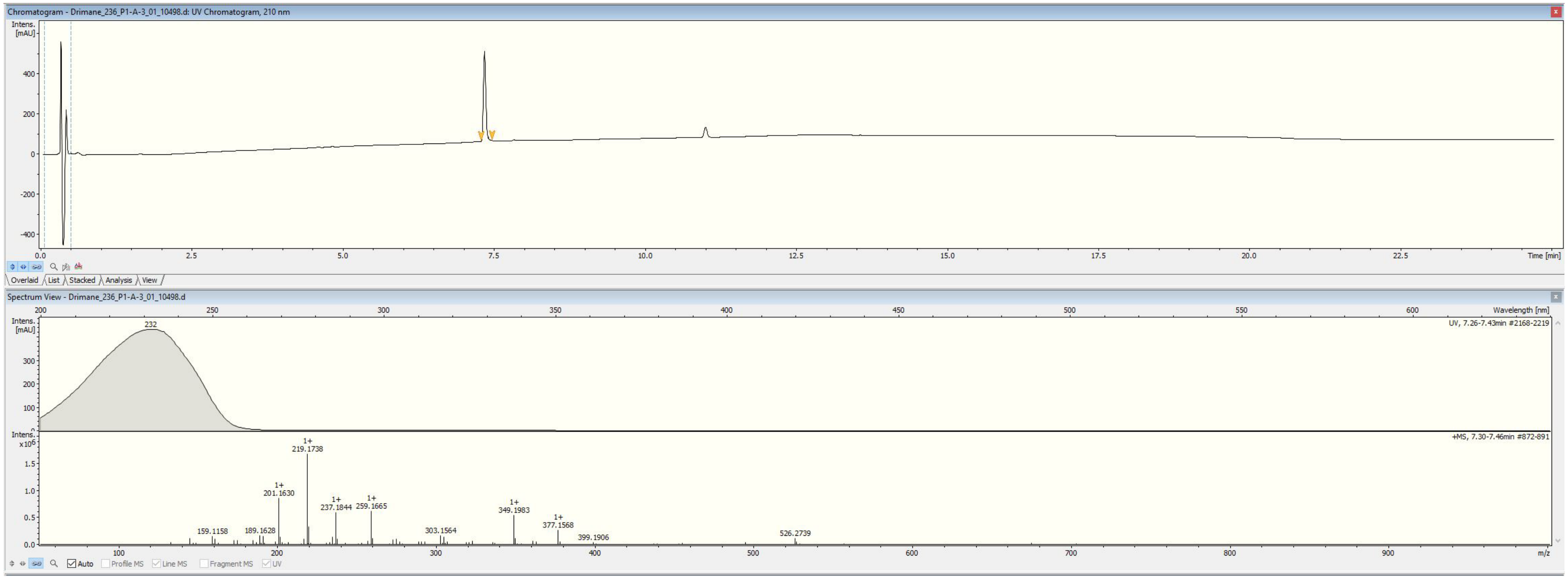

Figure S17: HR-ESIMS data of 6,7-Dehydro-isodrimenediol (**3**).

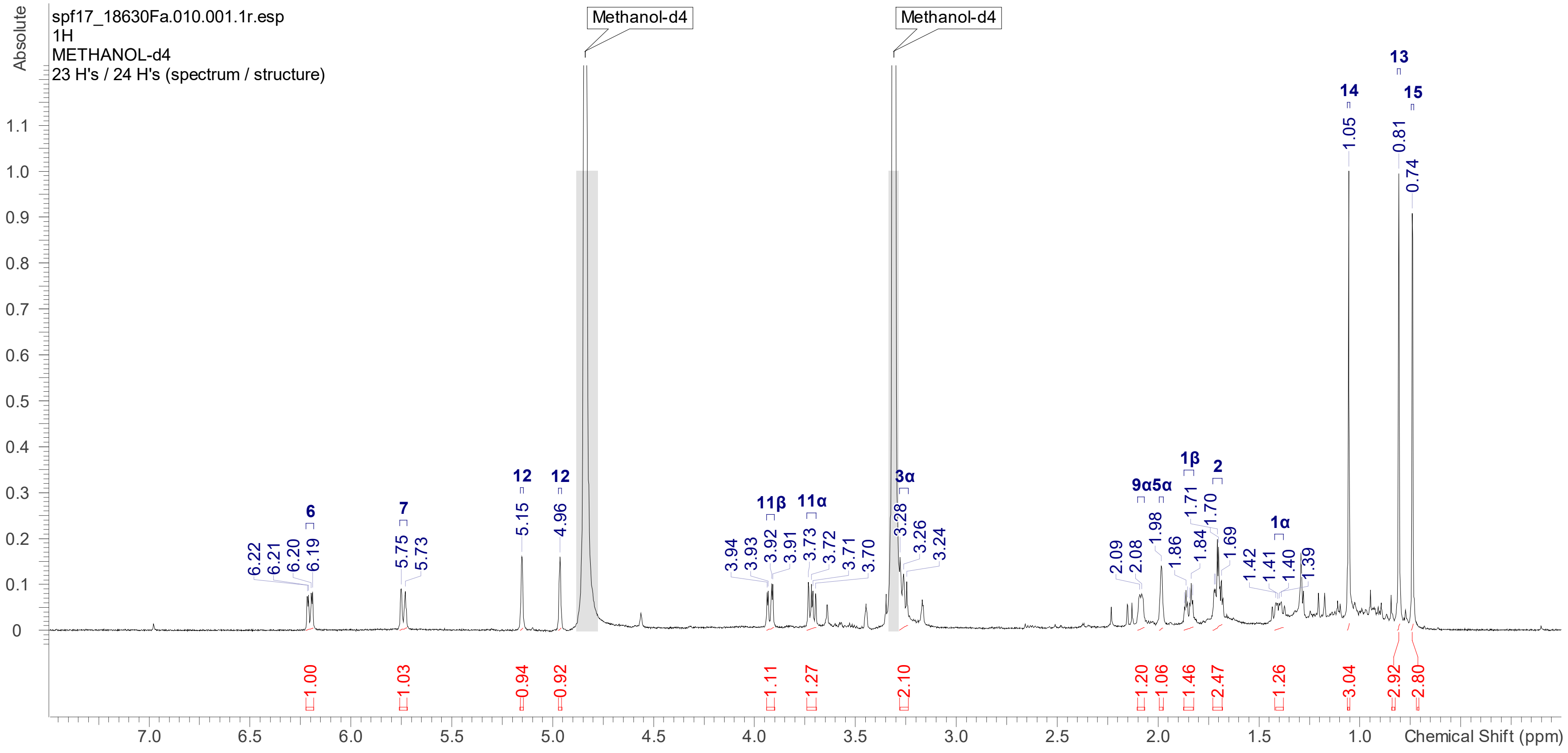

Figure S18: ^{1}H NMR spectrum (500 MHz, methanol-d_4) of 6,7-Dehydro-isodrimenediol (**3**).

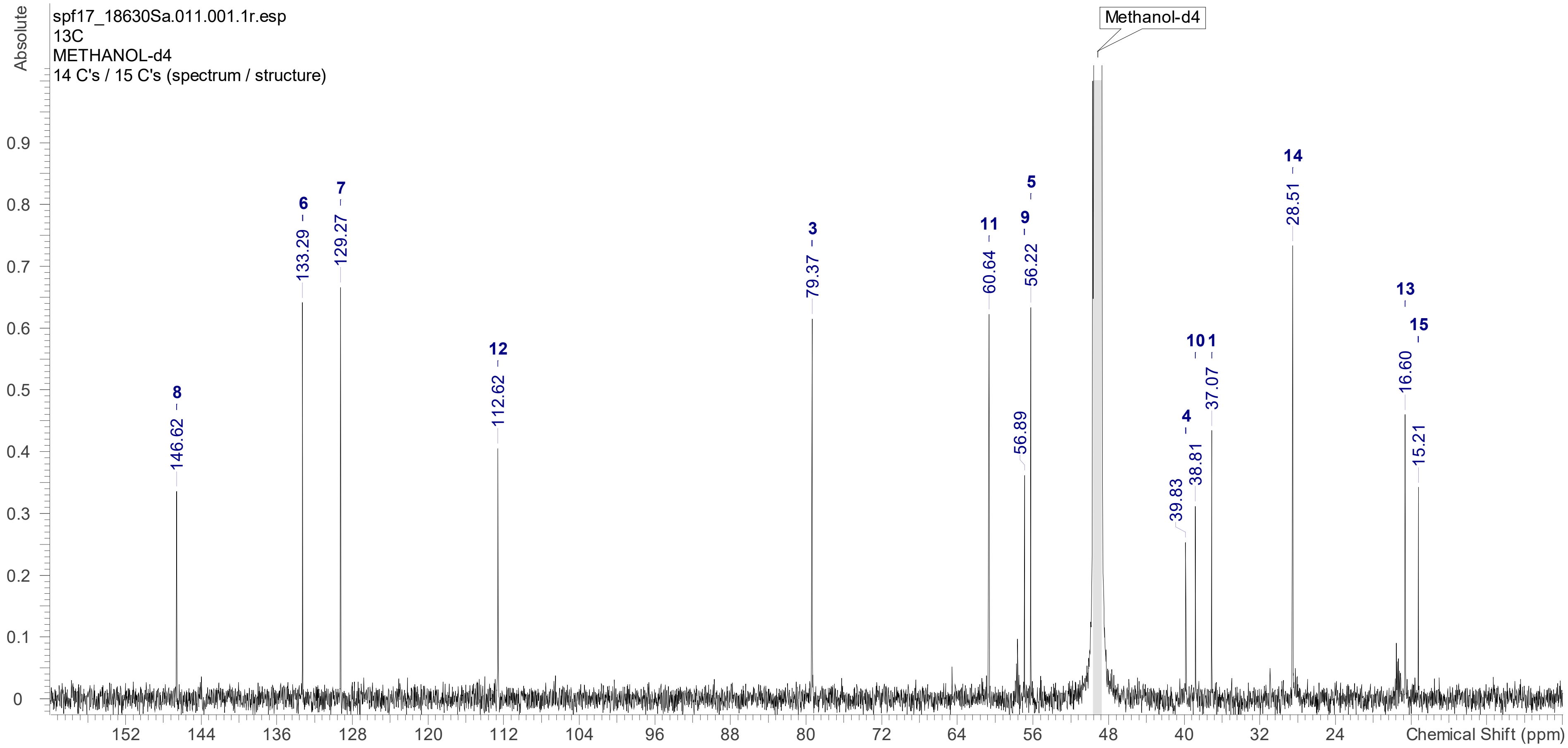

Figure S19: ^{13}C NMR spectrum (175 MHz, methanol-d_4) of 6,7-Dehydro-isodrimenediol (**3**).

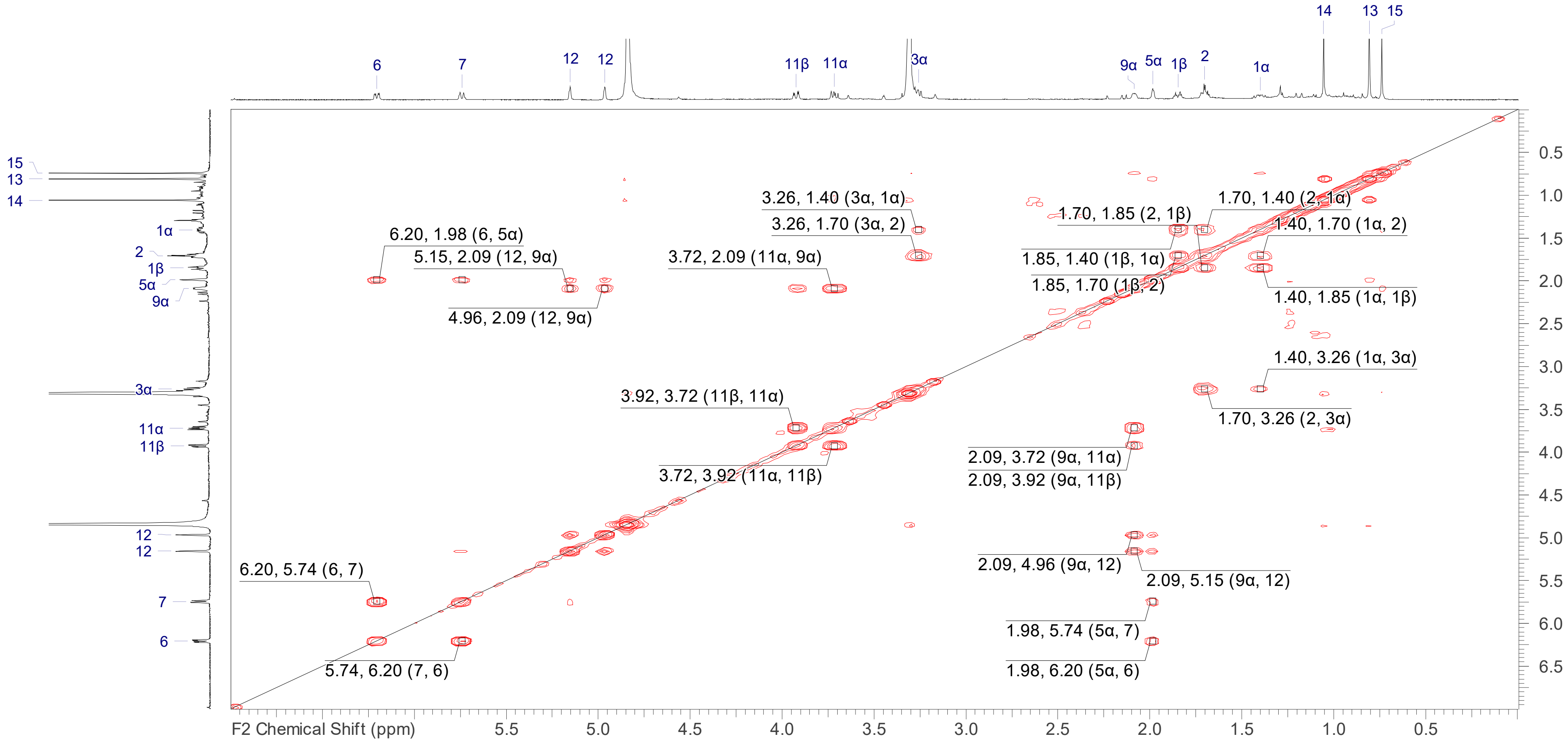

Figure S20: COSY NMR spectrum (500 MHz, methanol-d_4) of 6,7-Dehydro-isodrimenediol (**3**).

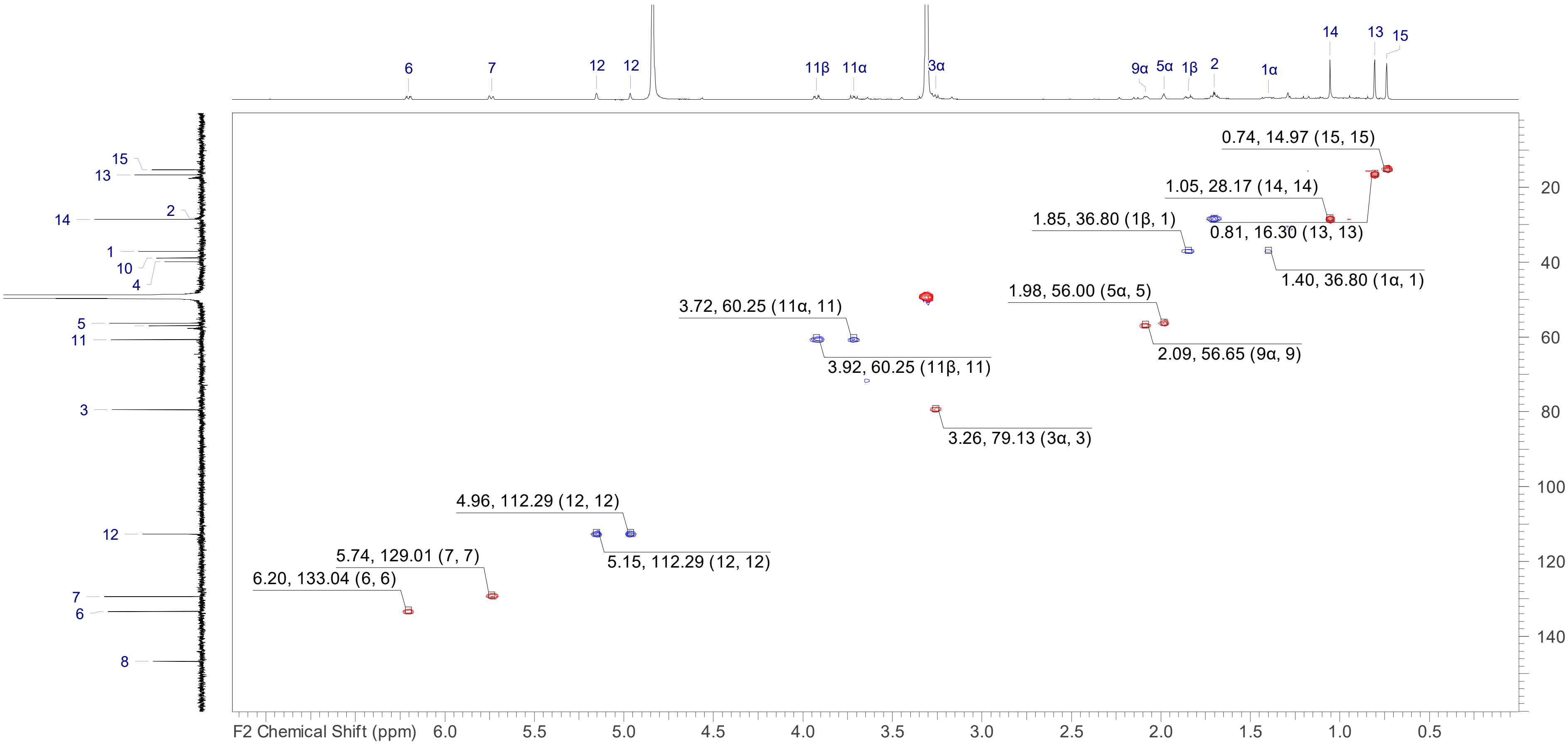

Figure S21: HSQC NMR spectrum (500 MHz, methanol-d_4) of 6,7-Dehydro-isodrimenediol (**3**).

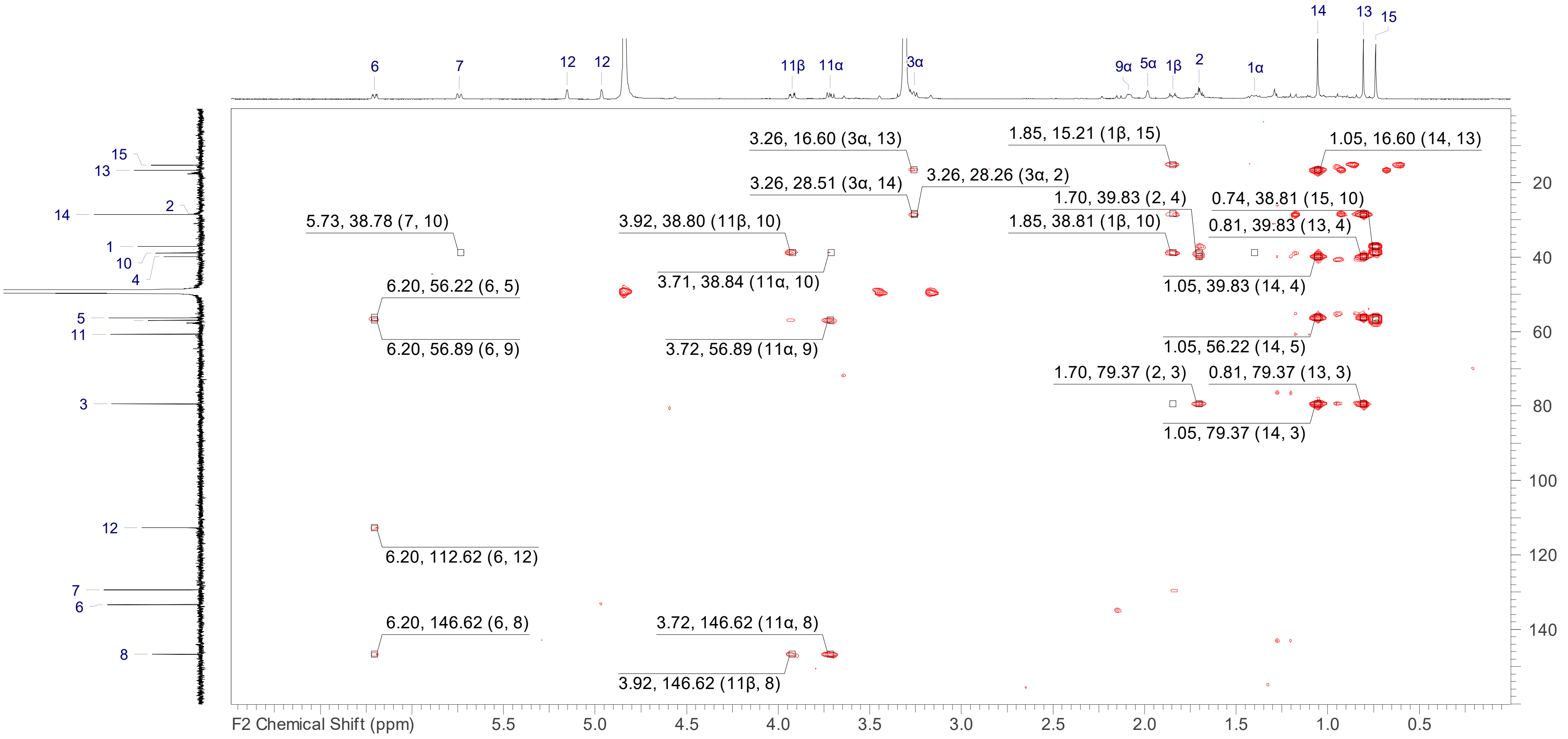

Figure S22: HMBC NMR spectrum (500 MHz, methanol-d_4) of 6,7-Dehydro-isodrimenediol (**3**).

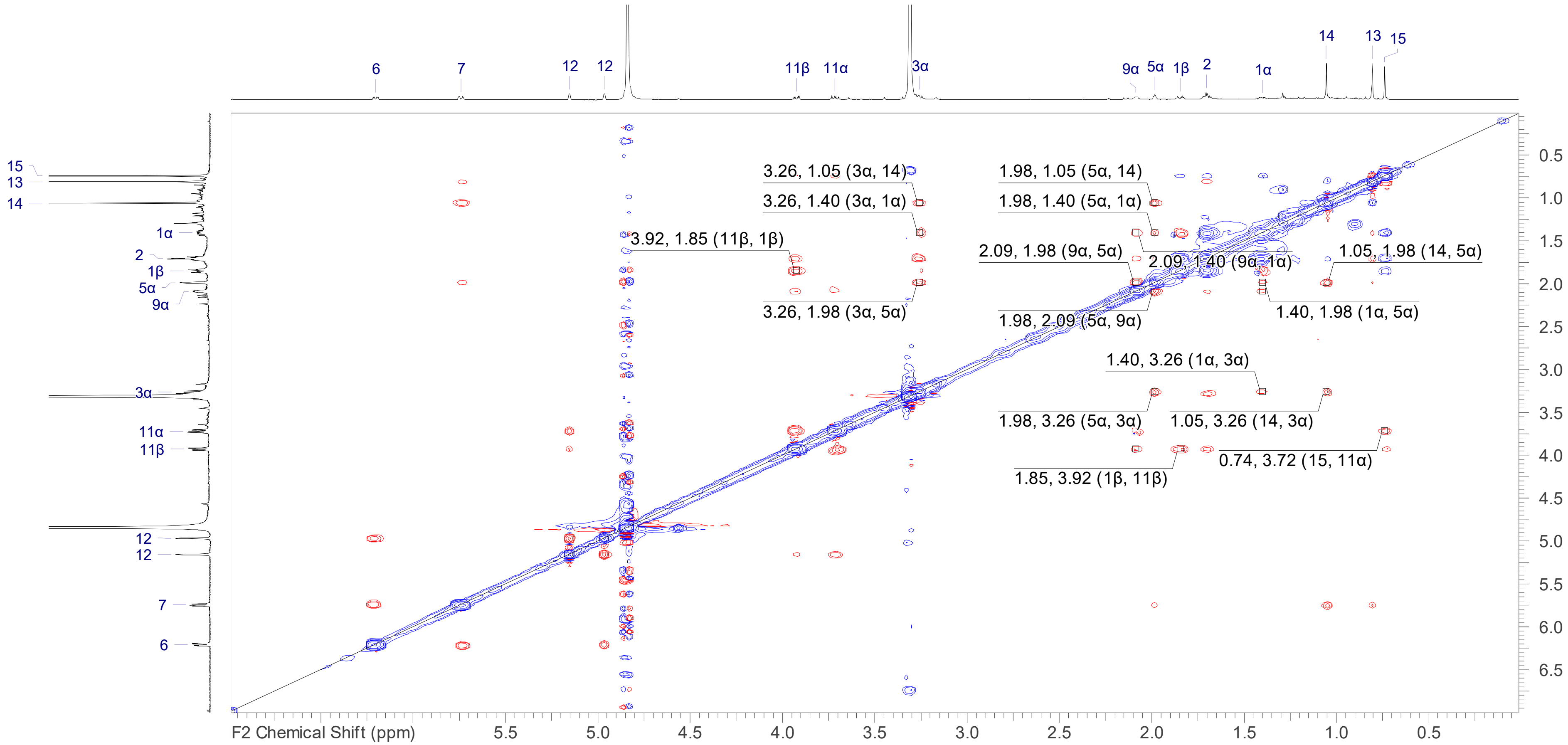

Figure S23: ROESY NMR spectrum (500 MHz, methanol-d_4) of 6,7-Dehydro-isodrimenediol (**3**).

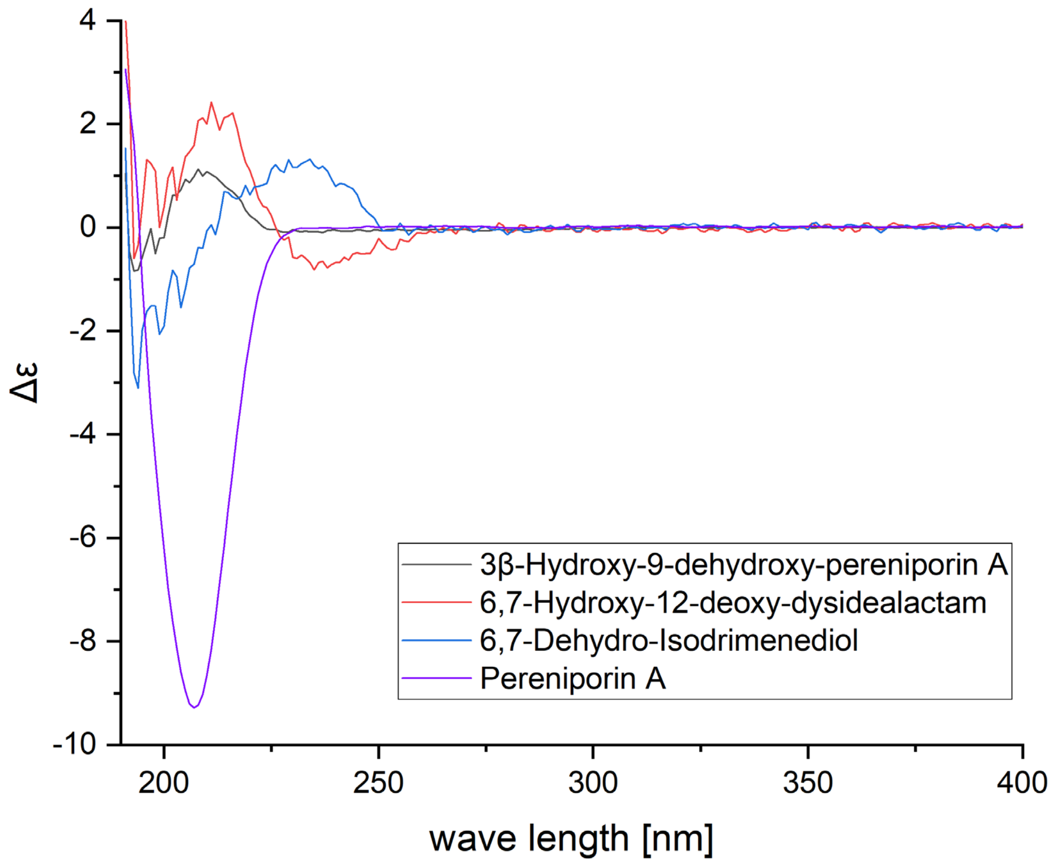

Figure S24: ECD spectra of **1**-**3** compared to Pereniporin A.

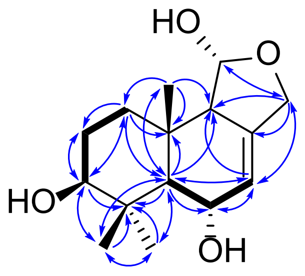

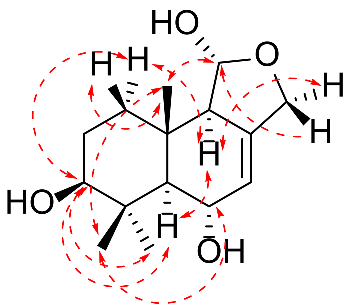

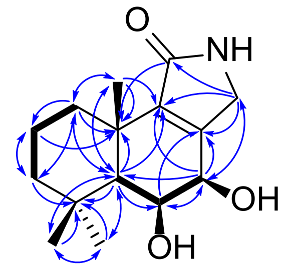

1 1 2

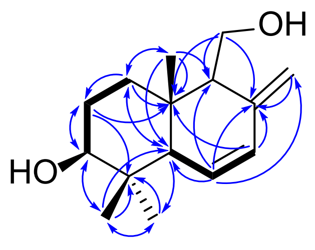

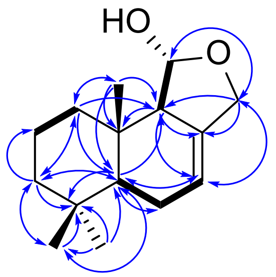

3 4

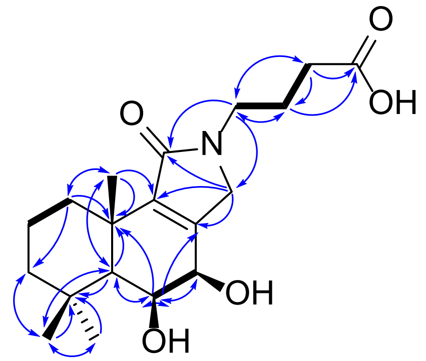

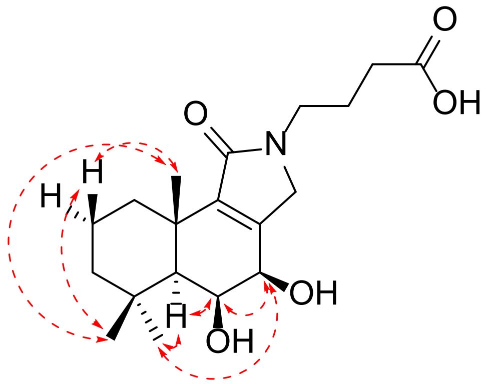

5 5

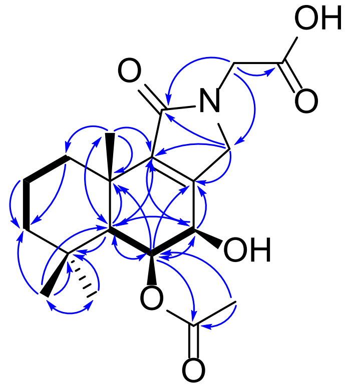

6

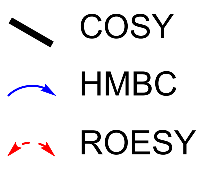

Figure S25: key COSY, HMBC and ROESY correlations indicating the structures of **1– 6.**

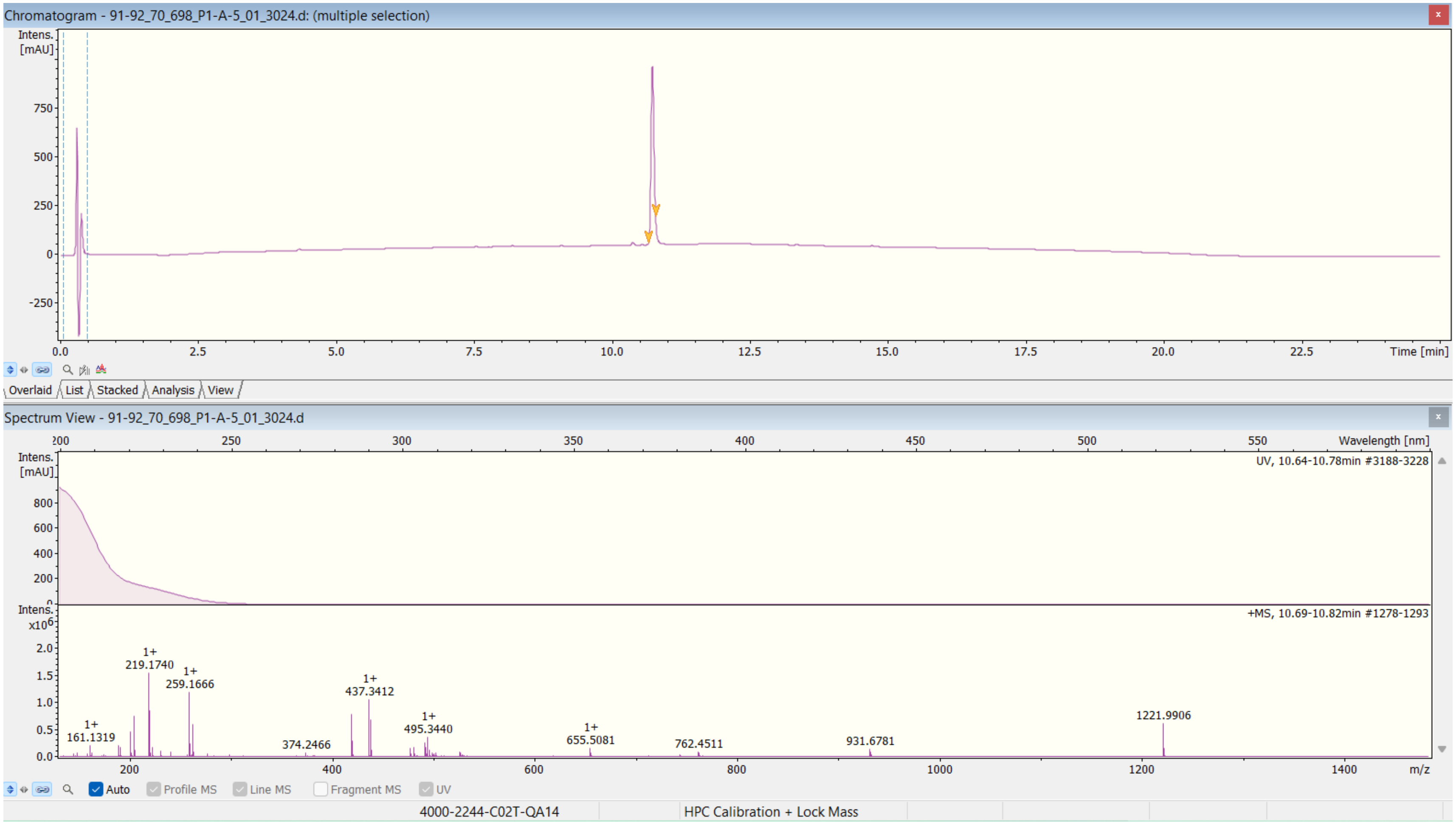

Figure S26: HR-ESIMS data of Isodrimeniol (**4**).

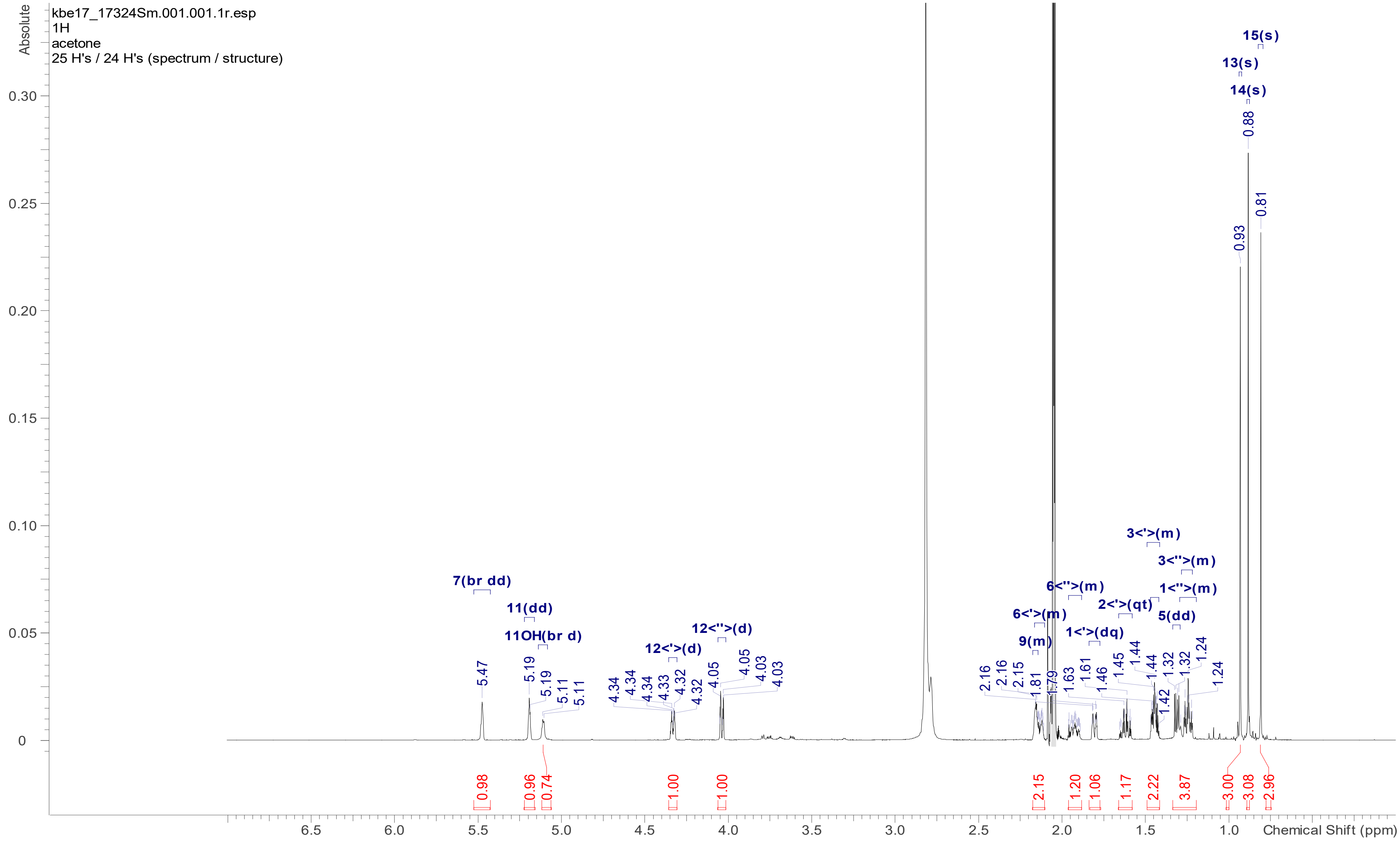

Figure S27: ^{1}H NMR spectrum (500 MHz) of Isodrimeniol (**4**) in acetone-d_6.

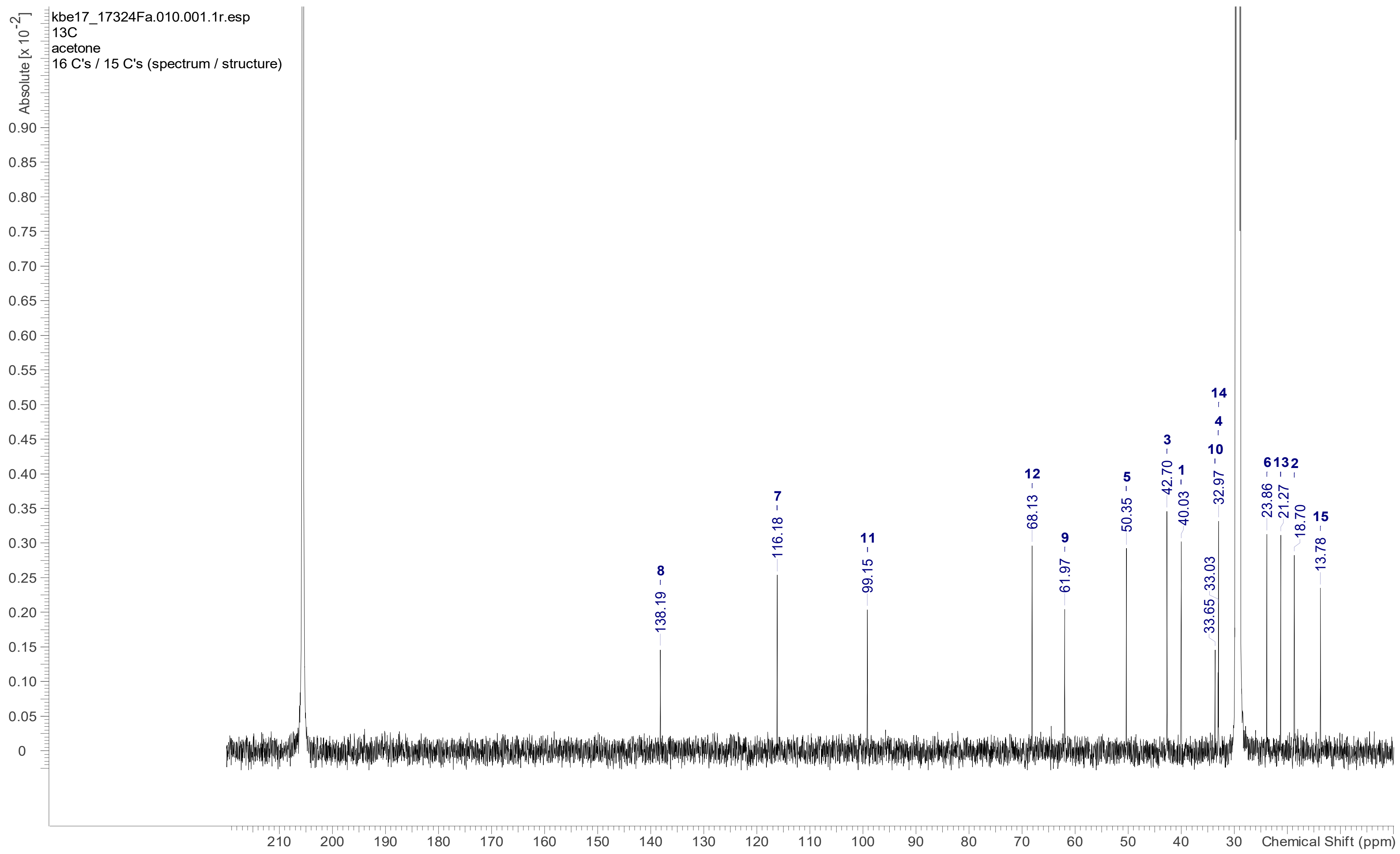

Figure S28: ^{13}C NMR spectrum (125 MHz) of Isodrimeniol (**4**) in acetone-d_6.

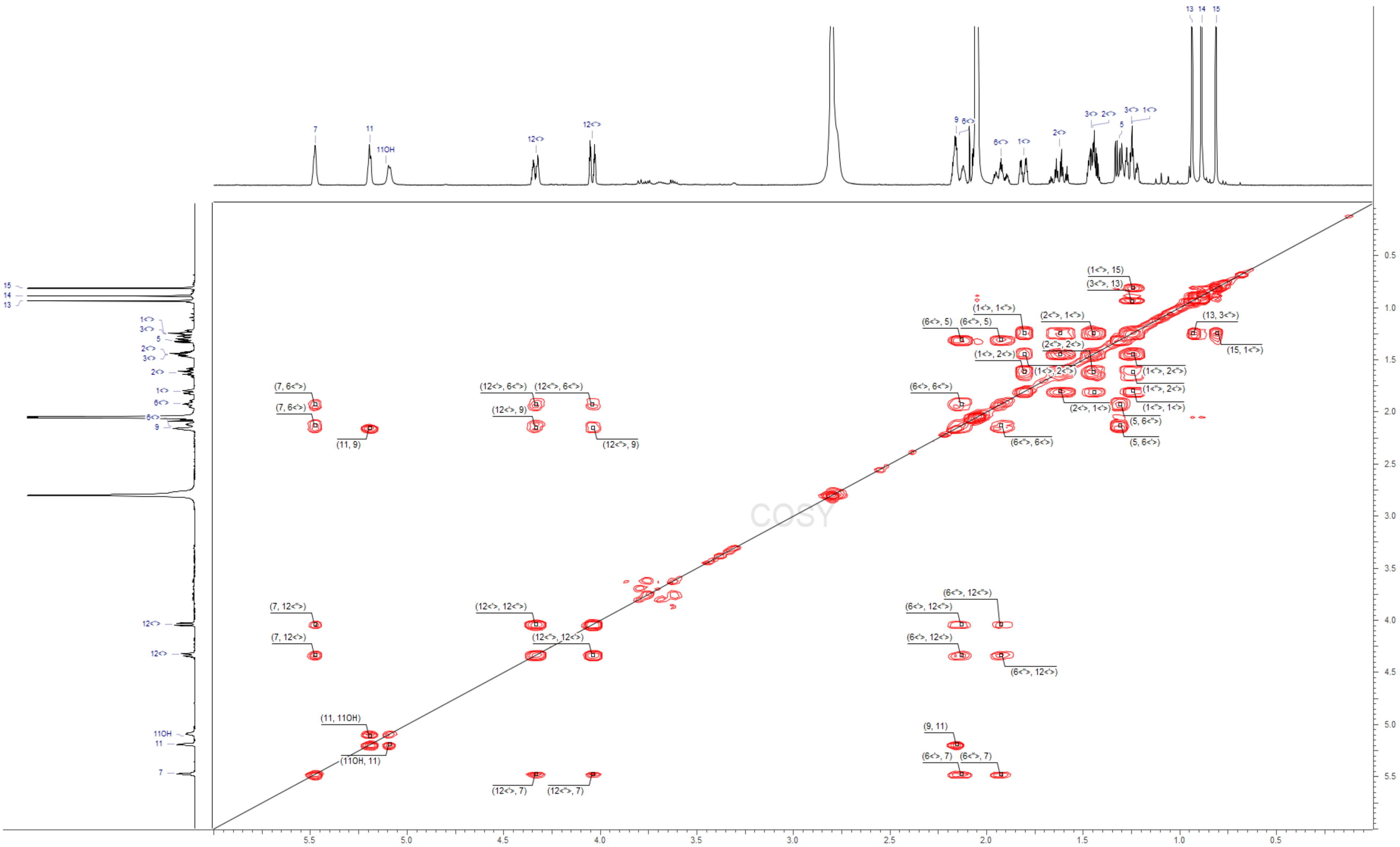

Figure S29: COSY NMR spectrum (500 MHz) of Isodrimeniol (**4**) in acetone-*d*₆.

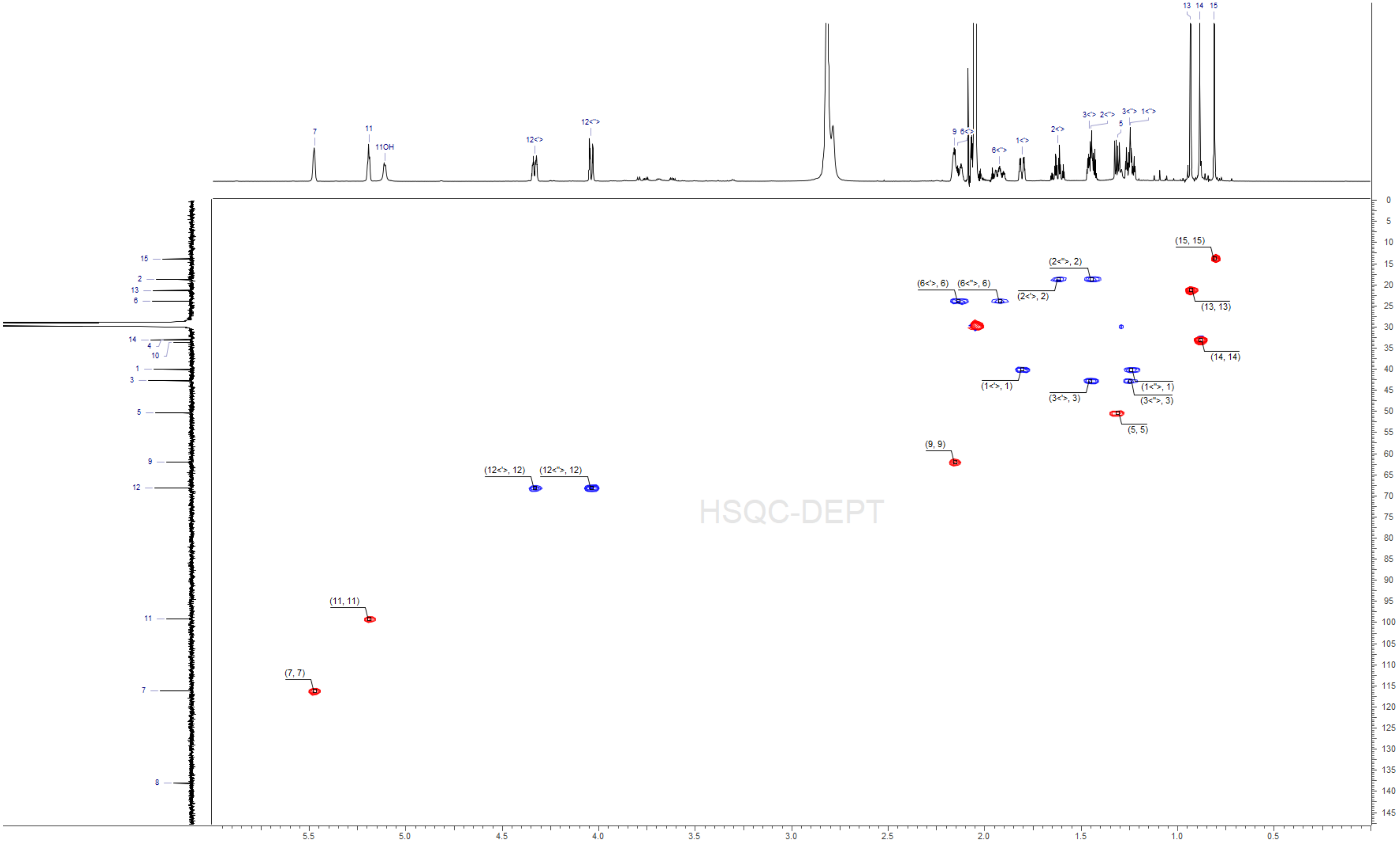

Figure S30: HSQC NMR spectrum (500 MHz) of Isodrimeniol (**4**) in acetone-d_6.

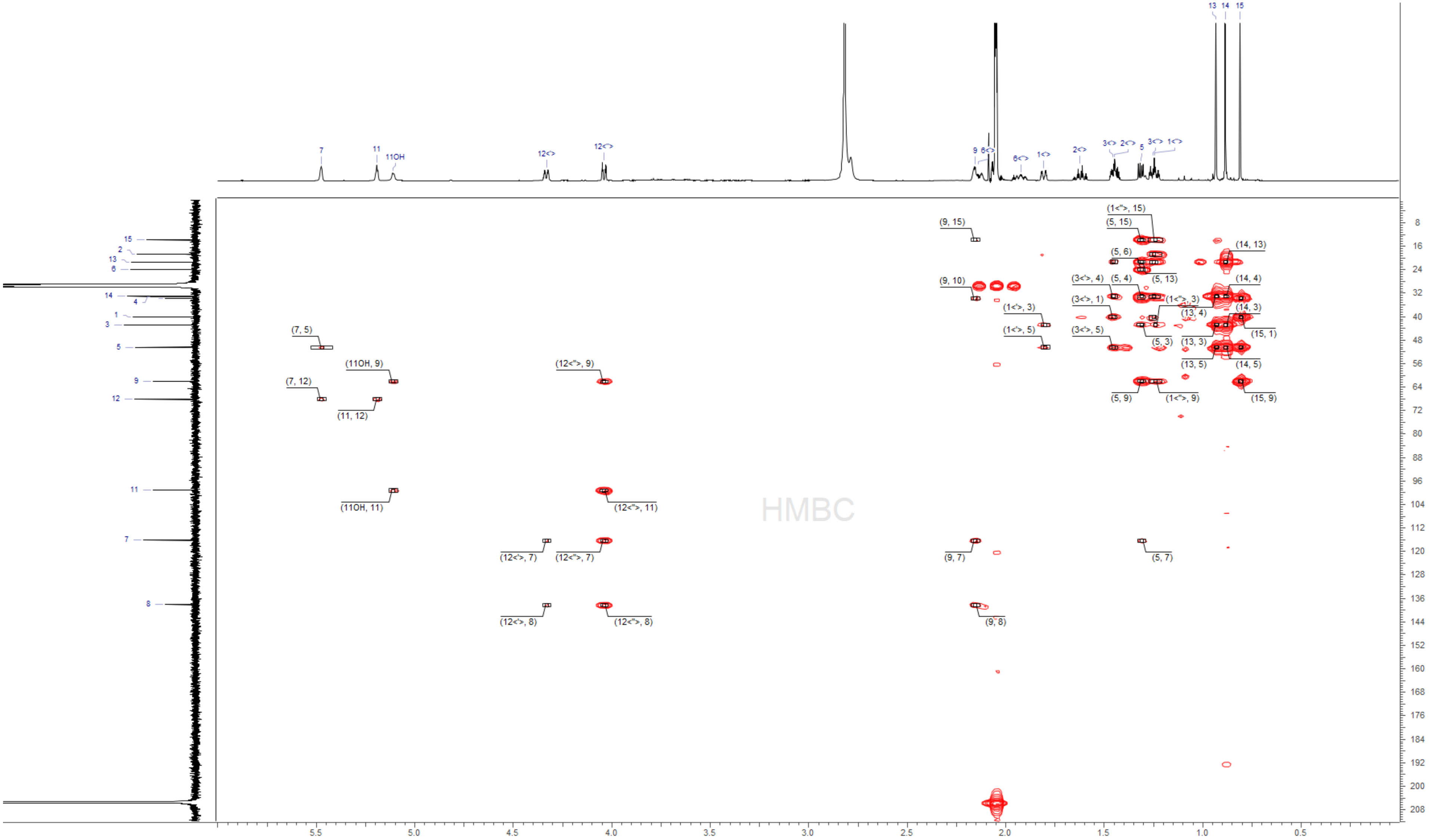

Figure S31: HMBC NMR spectrum (500 MHz) of Isodrimeniol (**4**) in acetone-d_6.

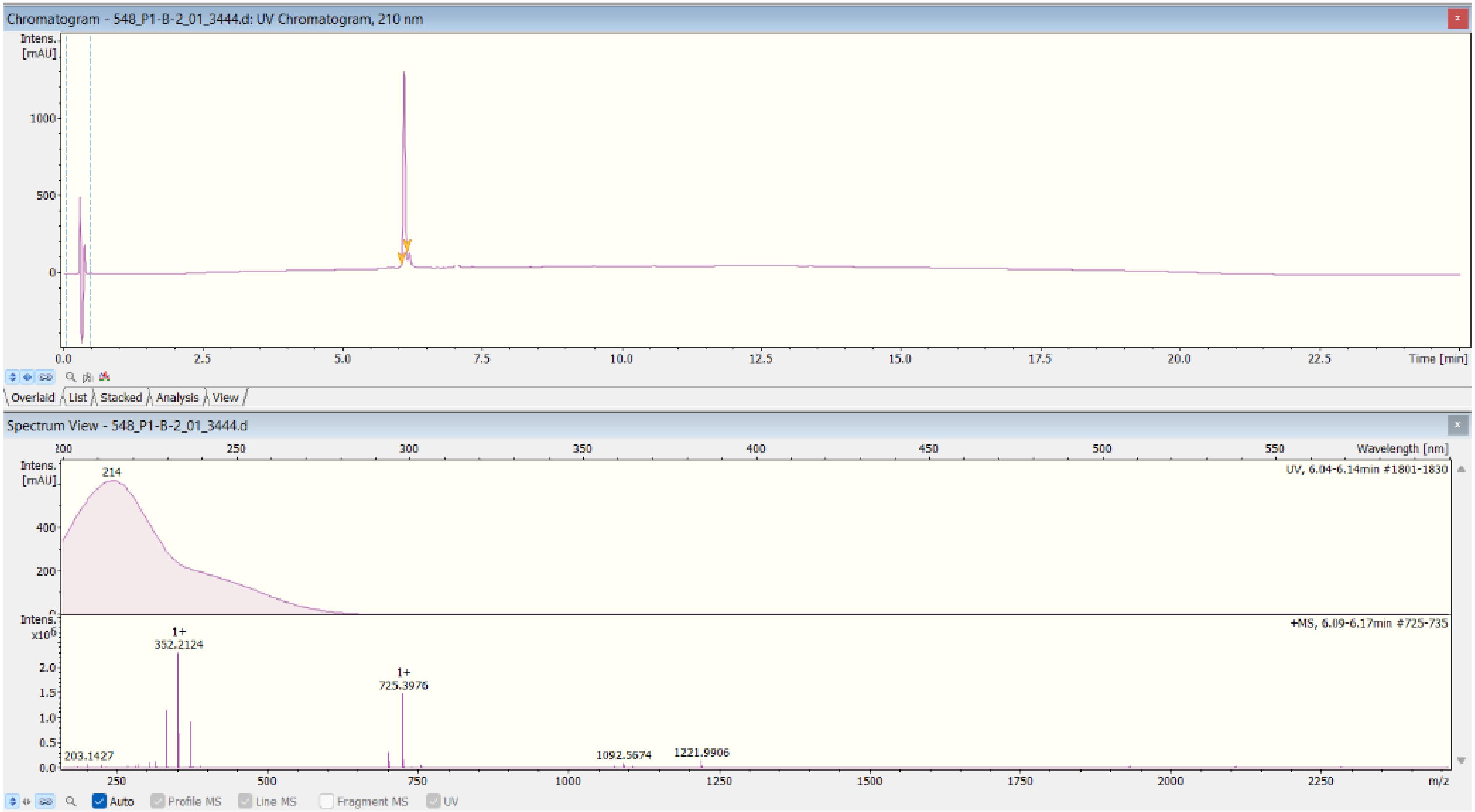

Figure S32: HR-ESIMS data of Glycinyl derivative of deacetylugandensolide (**5**).

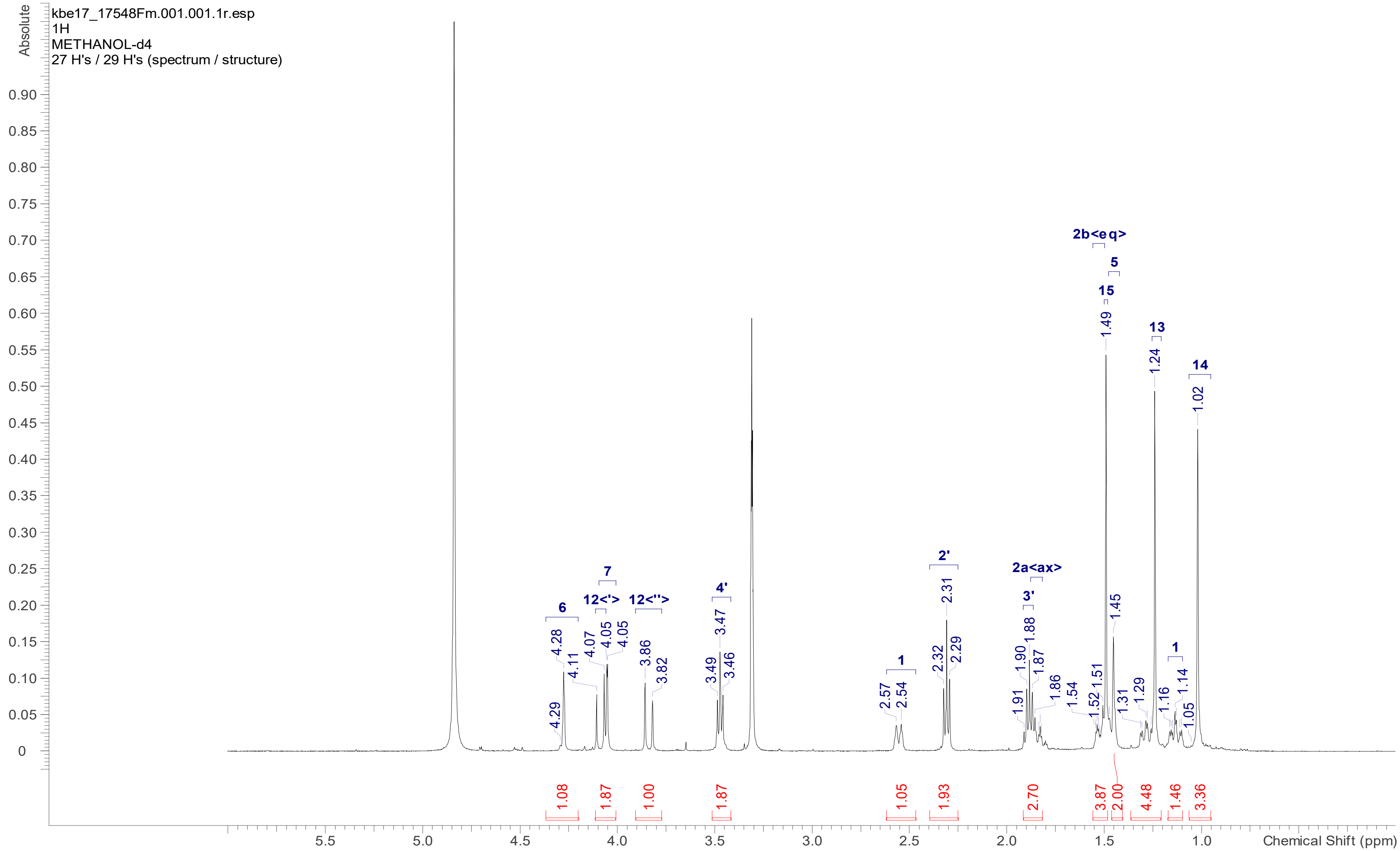

Figure S33: ^{1}H NMR spectrum (500 MHz) of Glycinyl derivative of deacetylugandensolide (**5**) in methanol-*d*4.

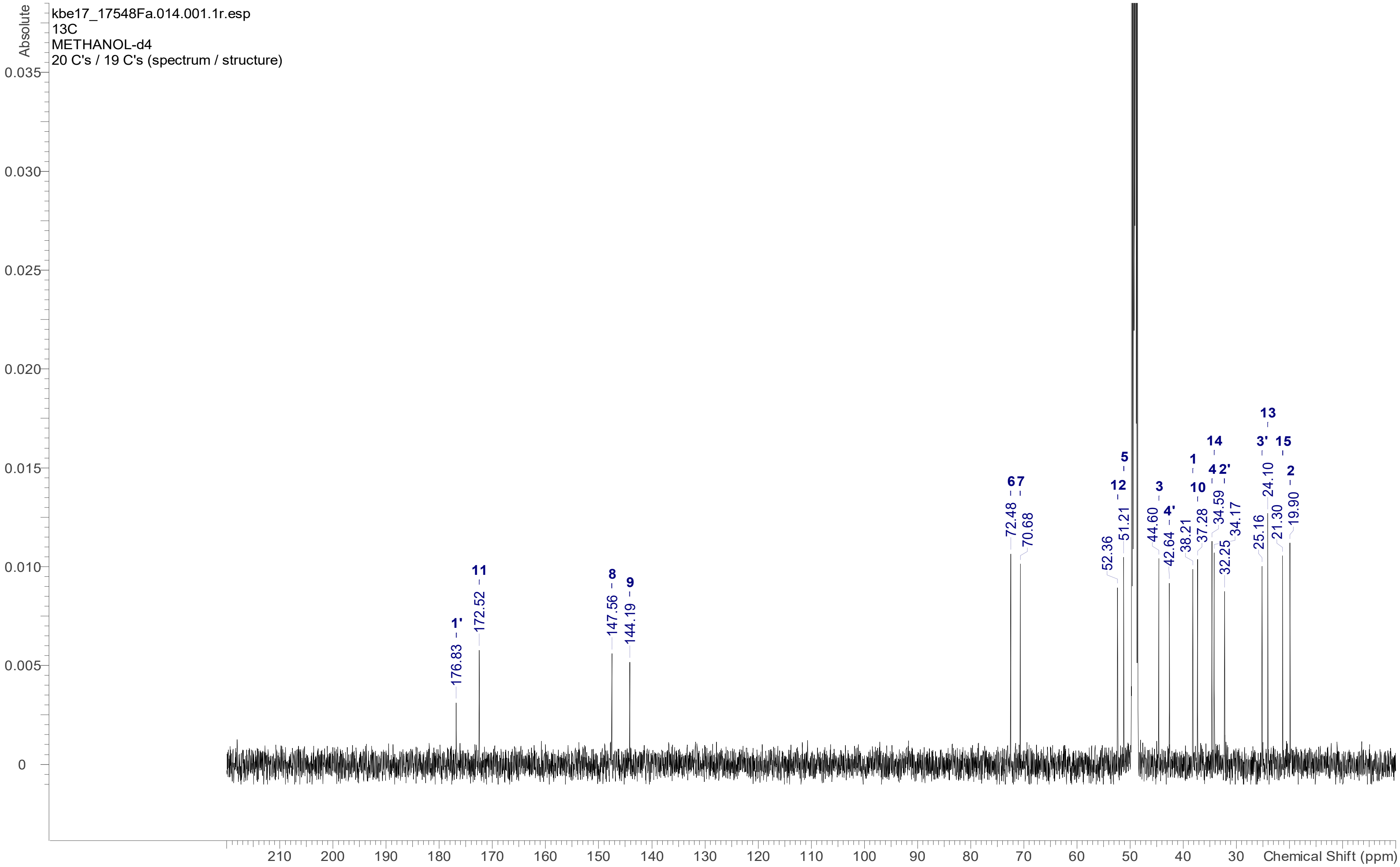

Figure S34: ^{13}C NMR spectrum (125 MHz) of Glycinyl derivative of deacetylugandensolide (**5**) in methanol-*d*$_4$.

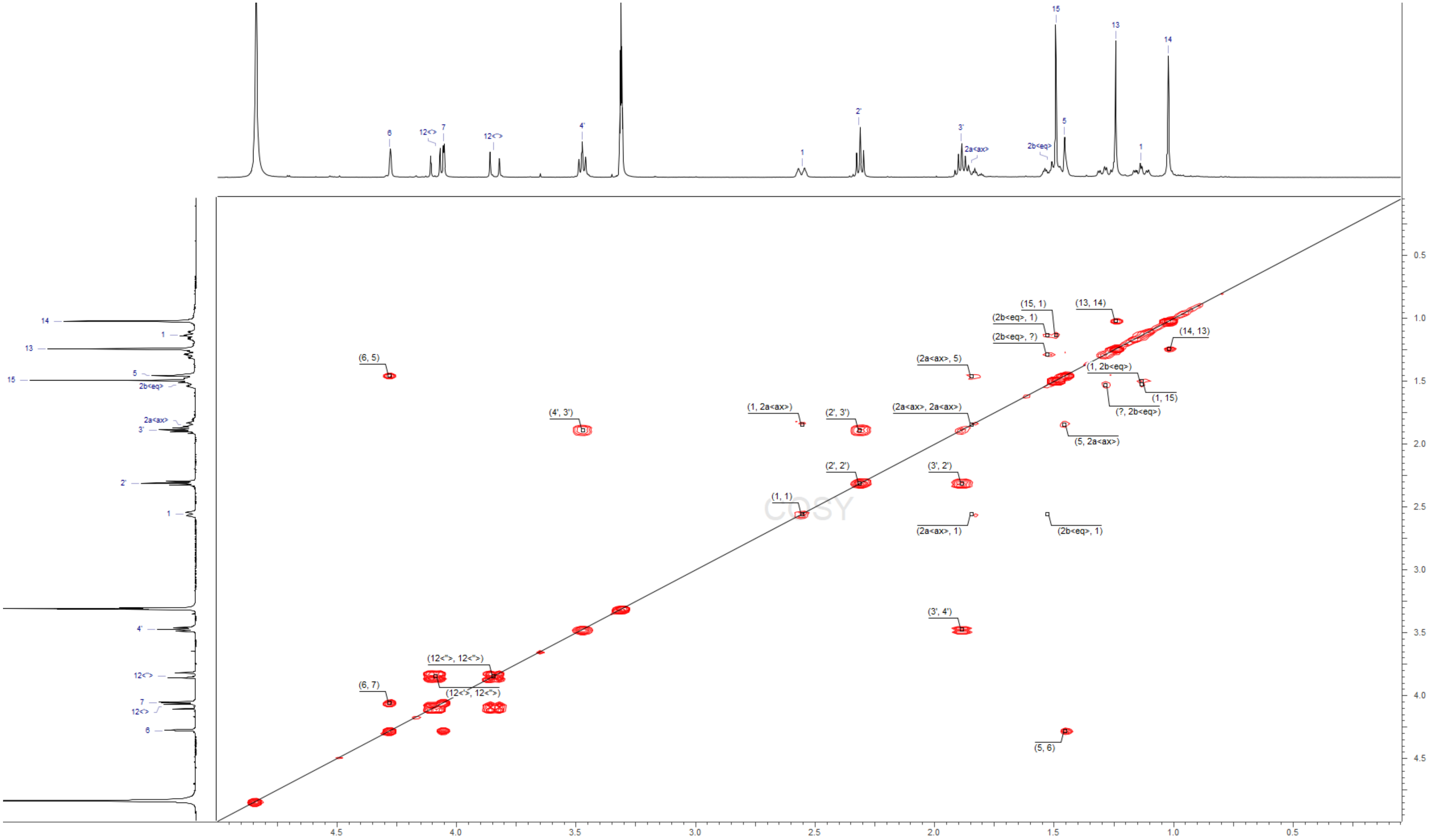

Figure S35: COSY NMR spectrum (500 MHz) of Glycinyl derivative of deacetylugandensolide (**5**) in methanol-*d*₄.

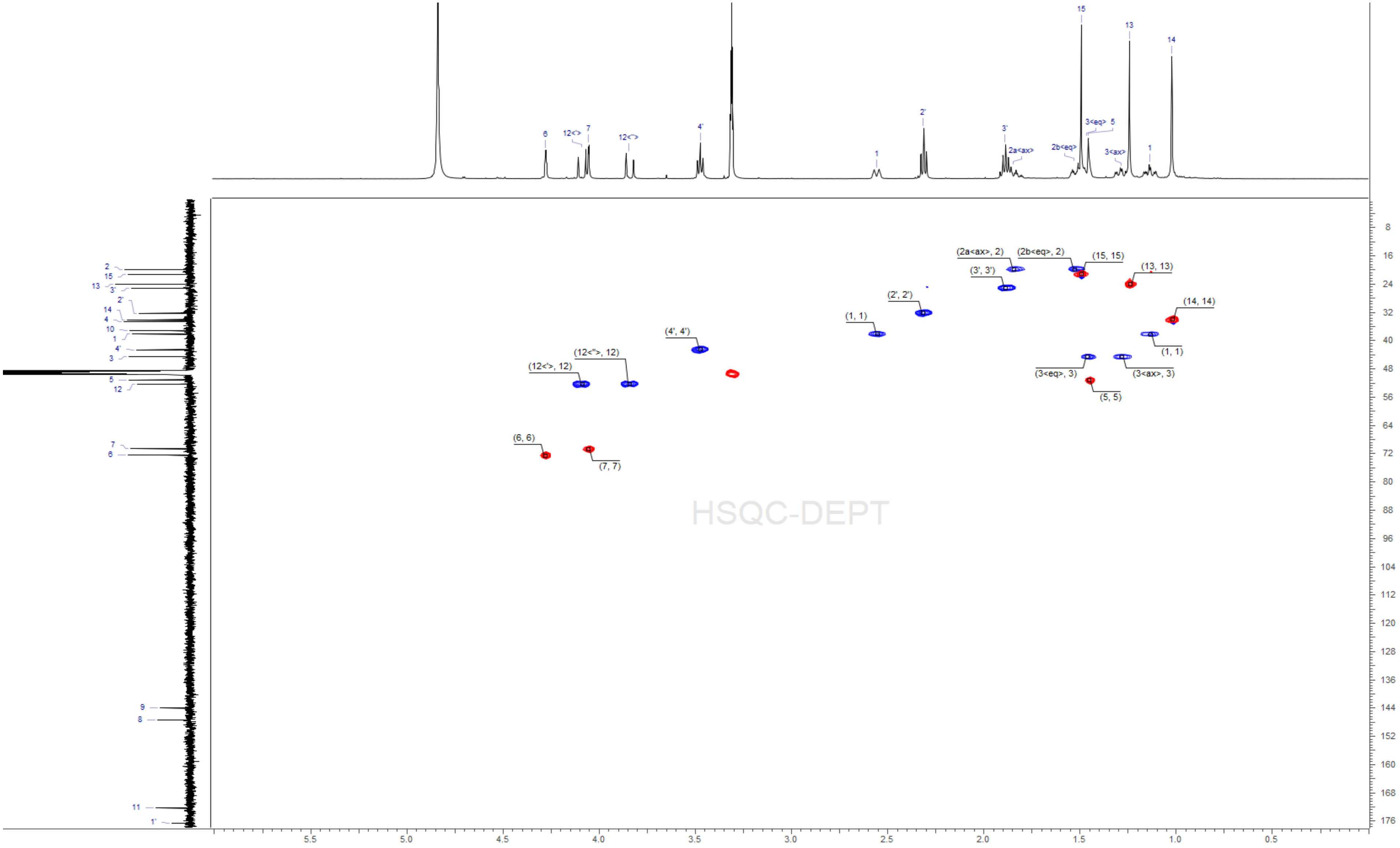

Figure S36: HSQC NMR spectrum (500 MHz) of Glycinyl derivative of deacetylugandensolide (**5**) in methanol-d_4.

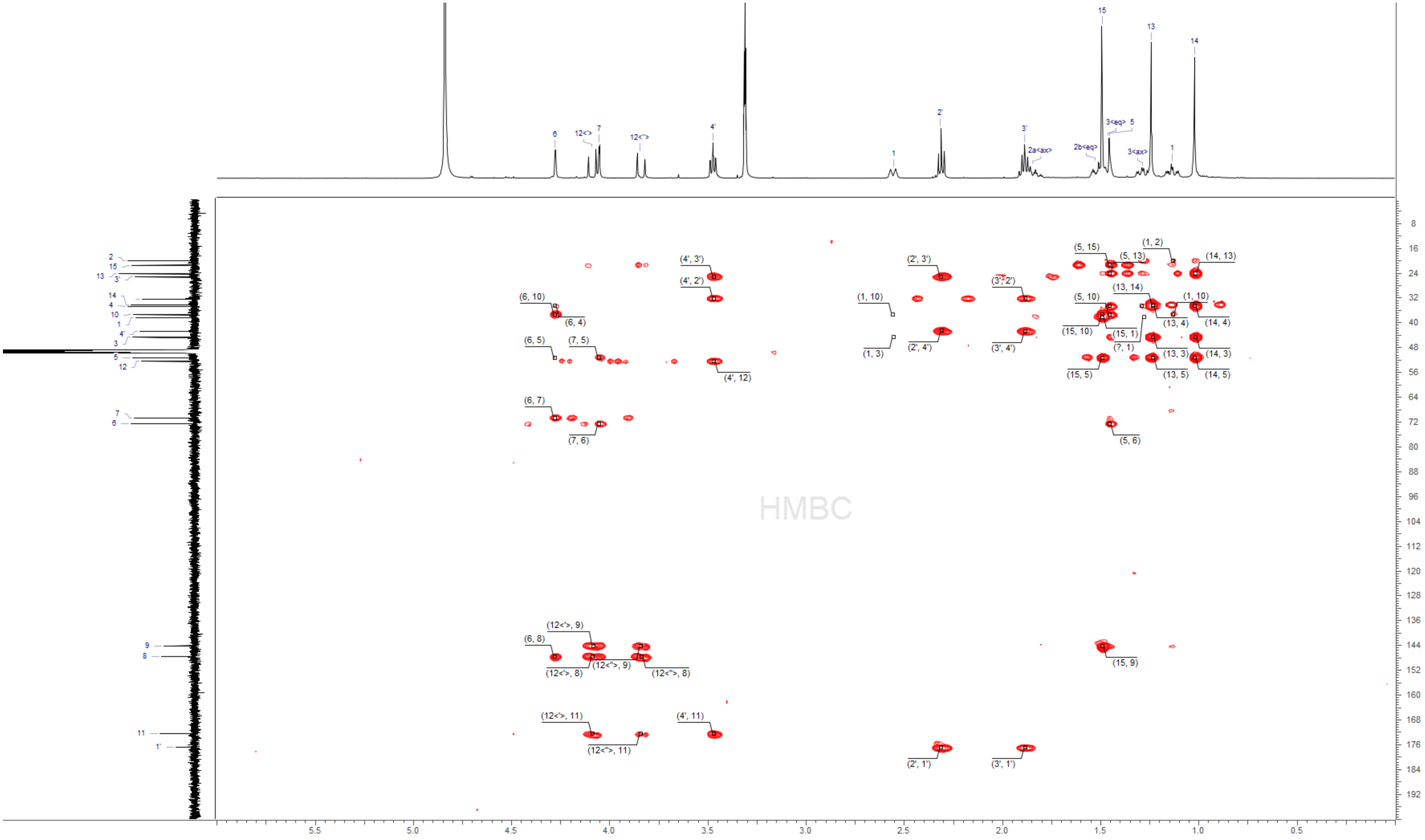

Figure S37: HMBC NMR spectrum (500 MHz) of Glycinyl derivative of deacetylugandensolide (**5**) in methanol-d_4.

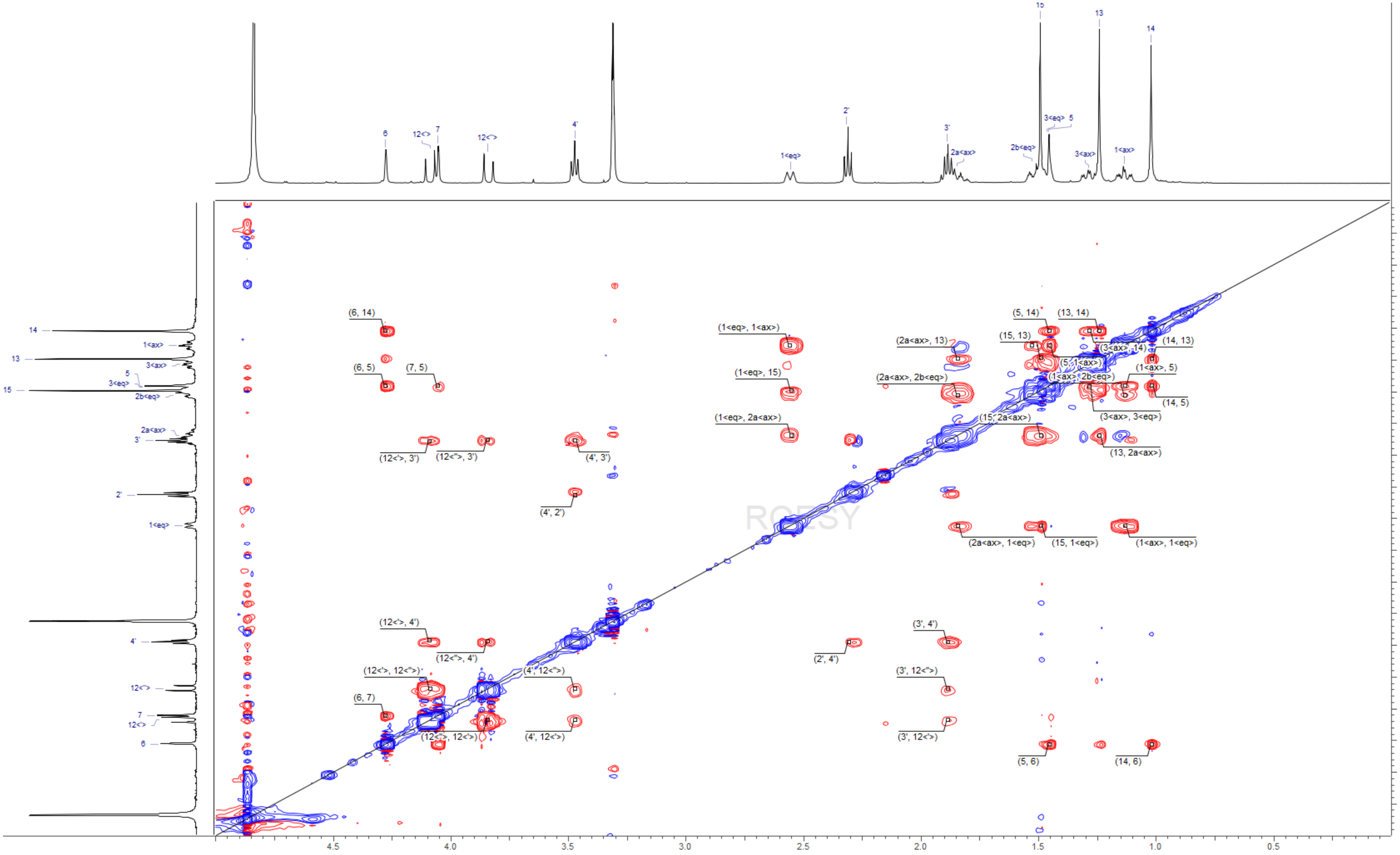

Figure S38: ROESY NMR spectrum (500 MHz) of Glycinyl derivative of deacetylugandensolide (**5**) in methanol-d_4.

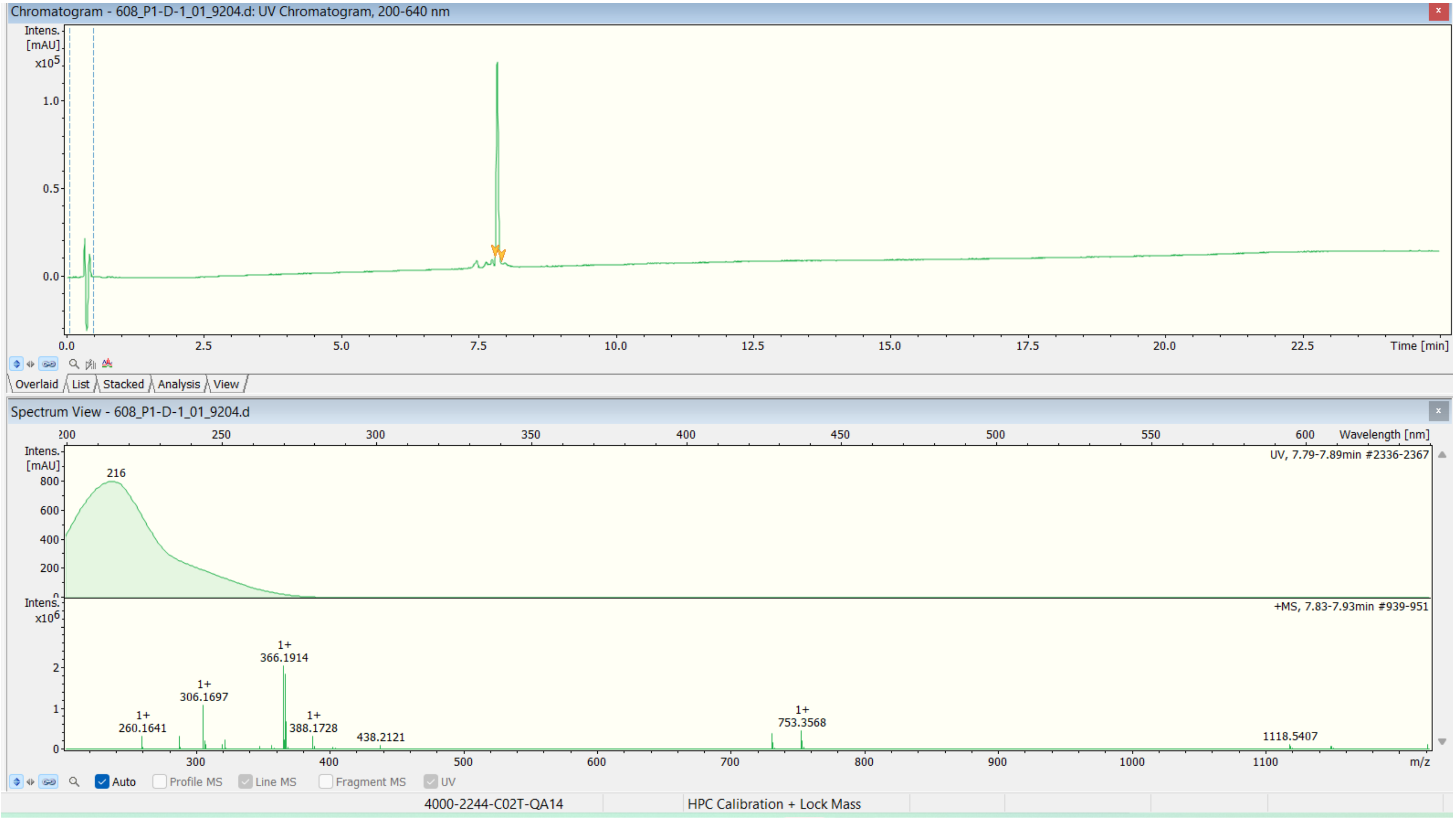

Figure S39: HR-ESIMS data of 4-Aminobutyl derivative of ugandensolide (**6**)

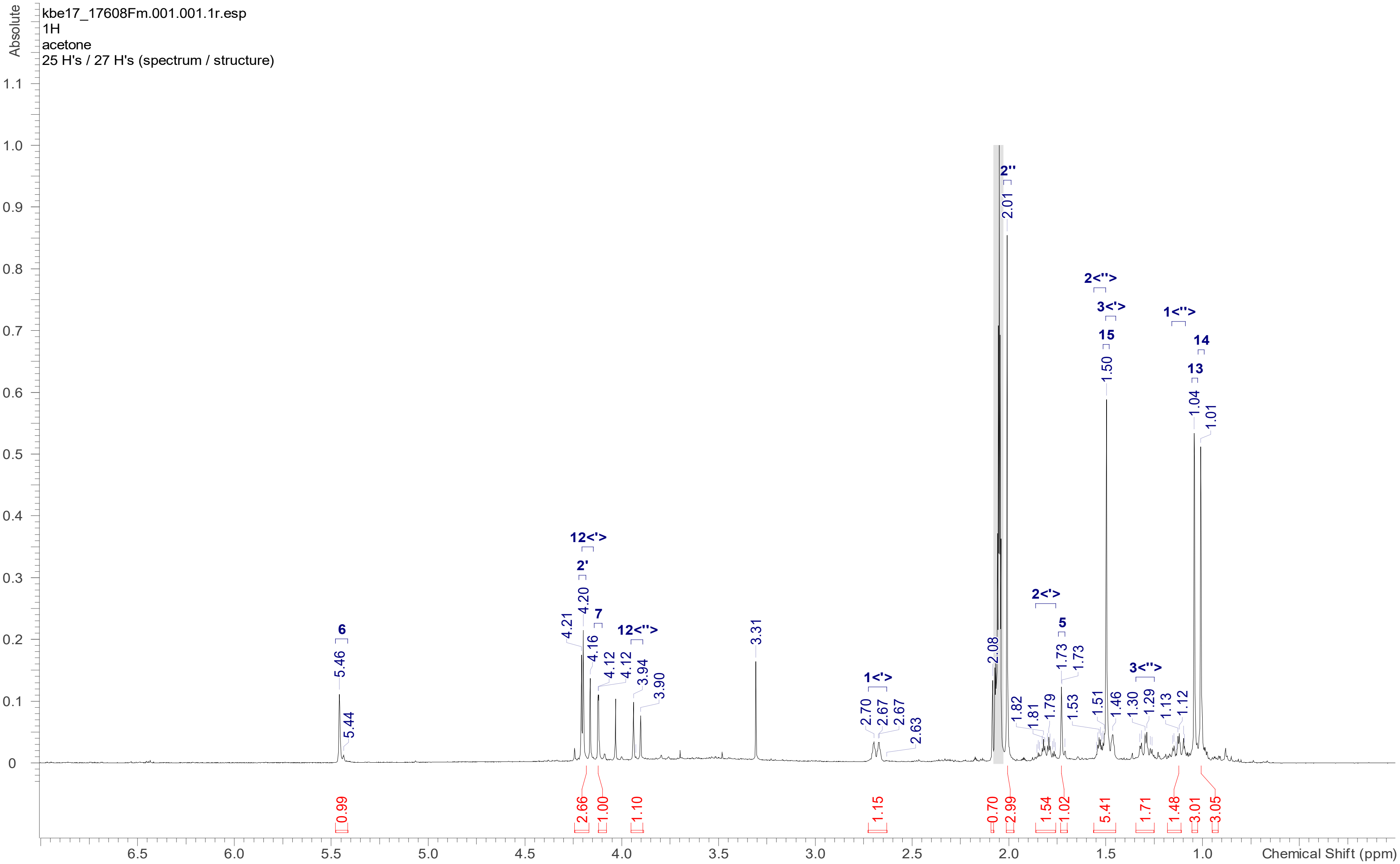

Figure S40: 1H NMR spectrum (500 MHz) of 4-Aminobutyl derivative of ugandensolide (**6**) in acetone-d_6.

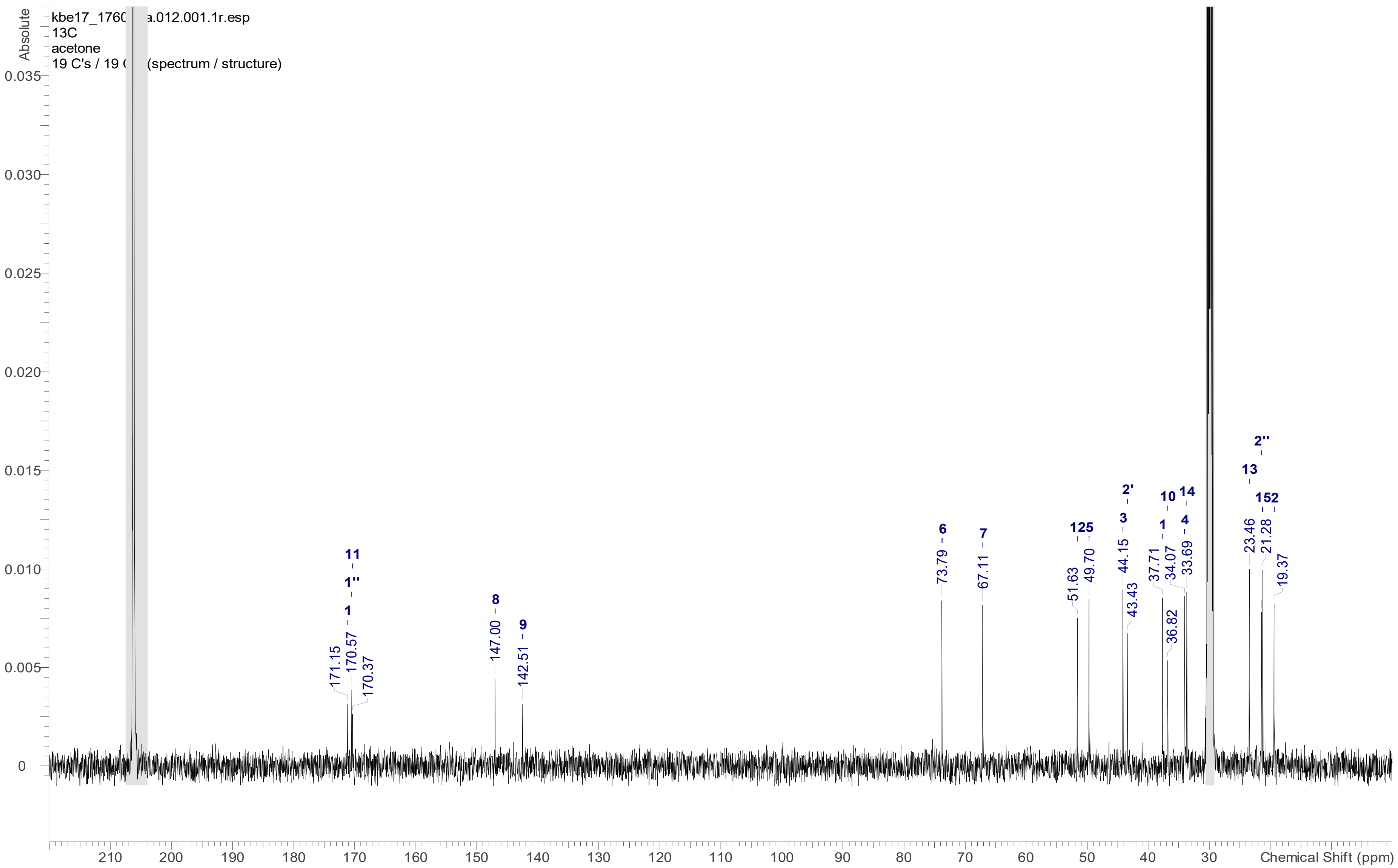

Figure S41: ^{13}C NMR spectrum (125 MHz) of 4-Aminobutyl derivative of ugandensolide (**6**) in acetone-d_6.

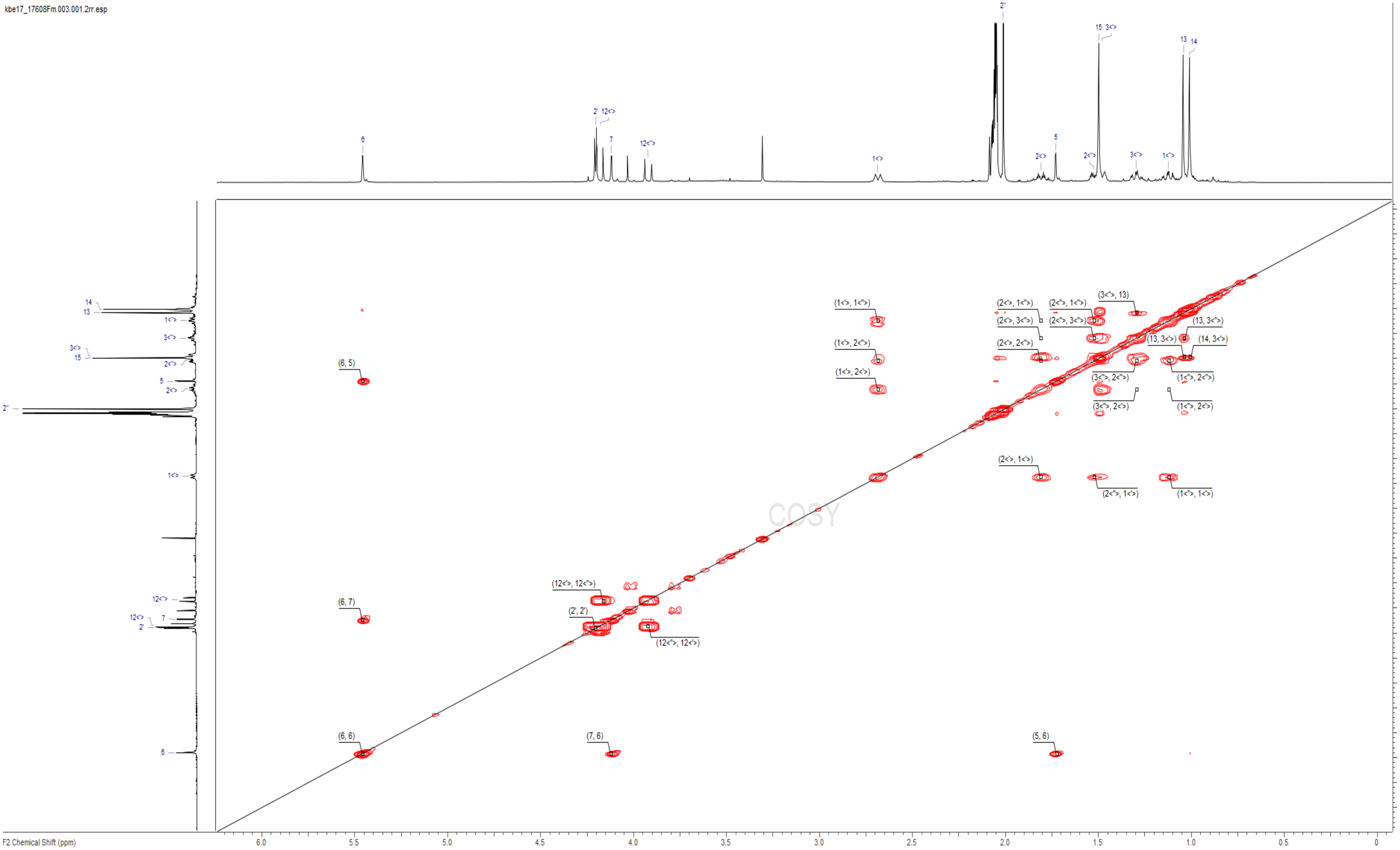

Figure S42: COSY NMR spectrum (500 MHz) of 4-Aminobutyl derivative of ugandensolide (**6**) in acetone-d_6.

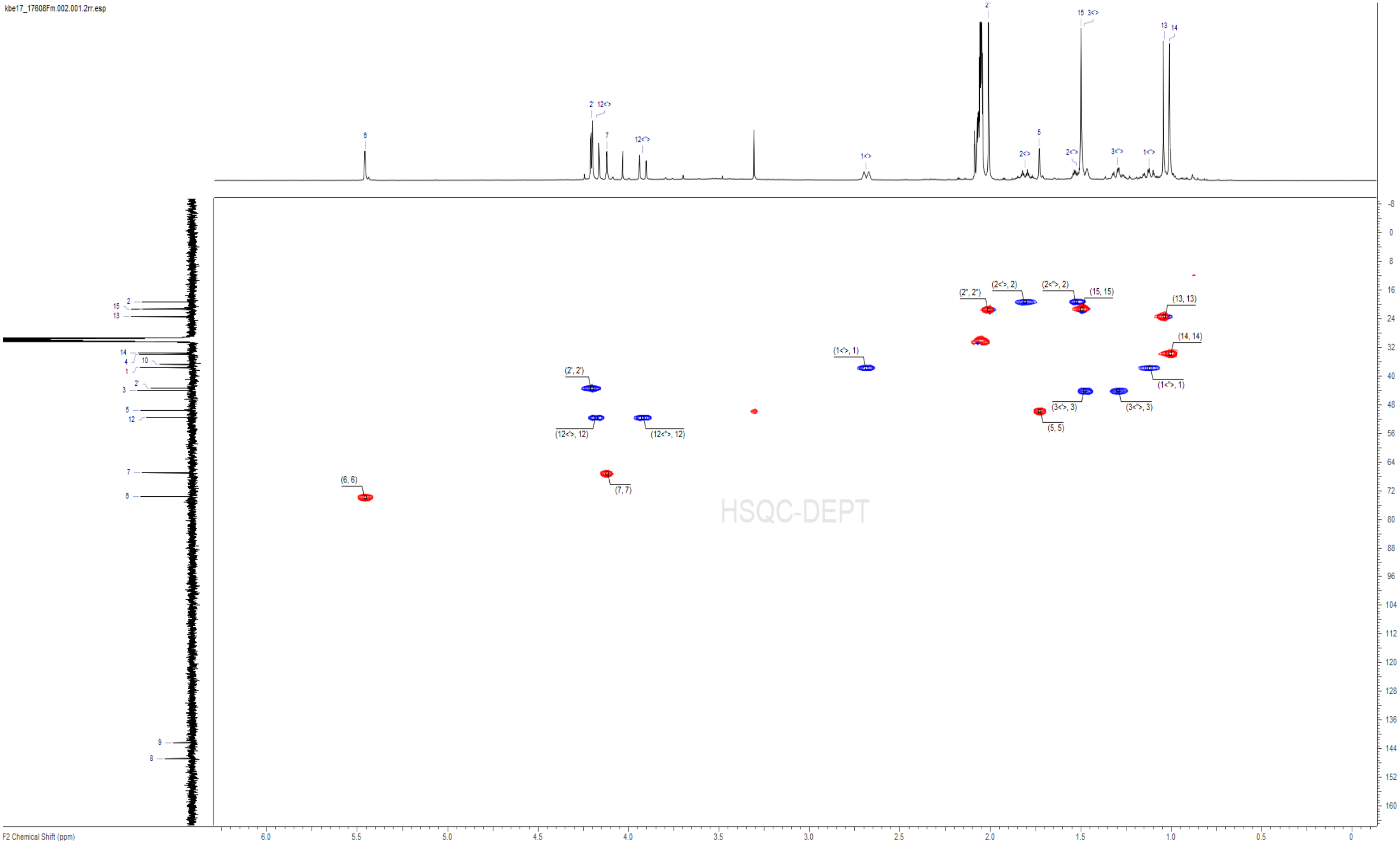

Figure S43: HSQC NMR spectrum (500 MHz) of 4-Aminobutyl derivative of ugandensolide (**6**) in acetone-*d*$_6$.

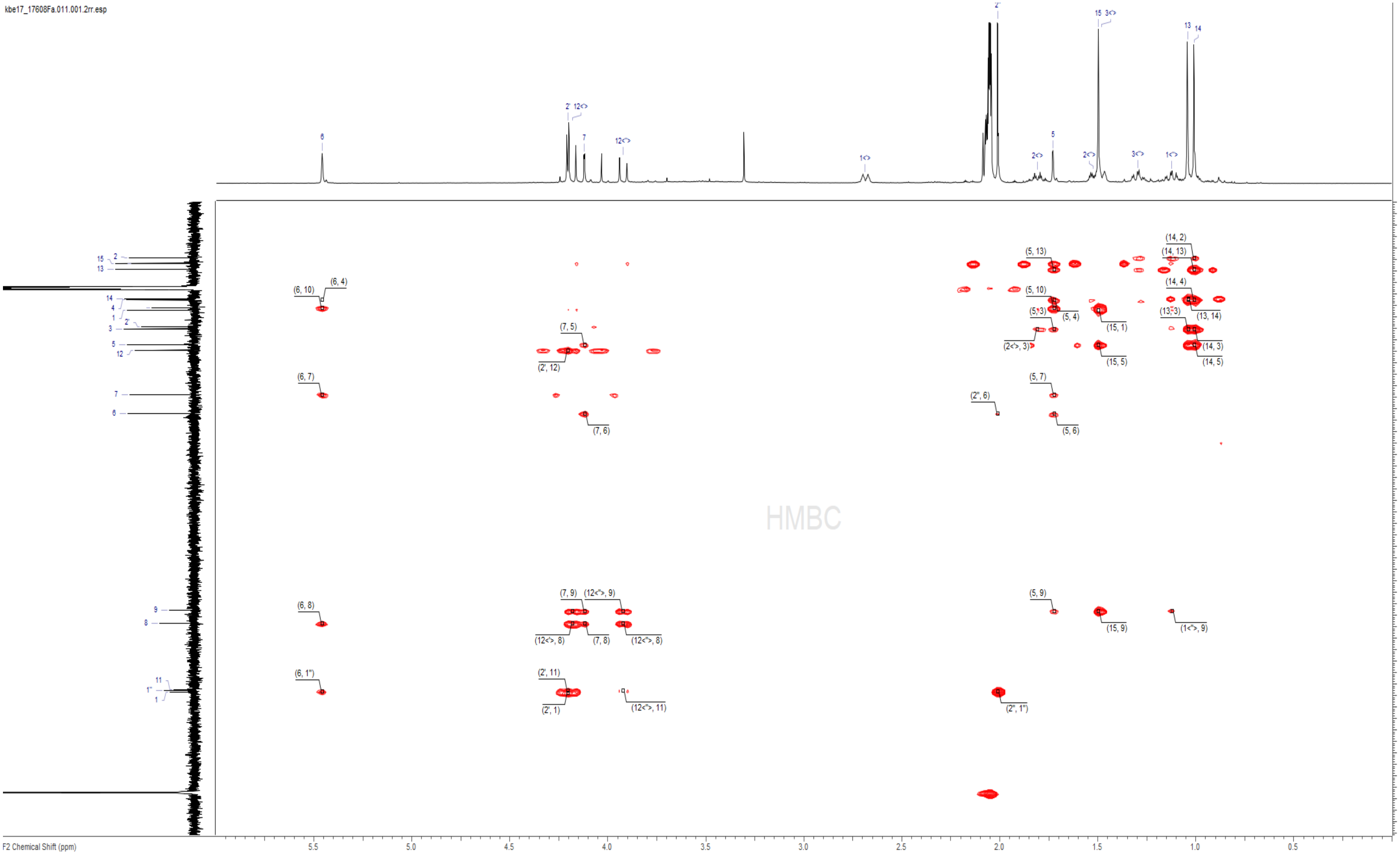

Figure S44: HMBC NMR spectrum (500 MHz) of 4-Aminobutyl derivative of ugandensolide (**6**) in acetone-*d*₆.

Table S2: Serial dilution assay parameters

**Test organisms**	**Strain-Nr.**	**Growth medium**	**Incubation temp. [°C]**	**Positive controls (references)**
Schizosaccharomyces pombe	DSM70572	MYC [1]	30	Nystatin 1.0 mg/mL
Pichia anomala	DSM6766	MYC [1]	30	Nystatin 1.0 mg/mL
Mucor hiemalis	DSM2656	MYC [1]	30	Nystatin 1.0 mg/mL
Candida albicans	DSM1665	MYC [1]	30	Nystatin 1.0 mg/mL
Rhodotorula glutinis	DSM10134	MYC [1]	30	Nystatin 1.0 mg/mL
Acinetobacter baumanii	DSM30008	MHB [2]	30	Ciprobay 2.54 mg/mL
Escherichia coli	DSM1116	MHB [2]	37	Oxytetracyclin 1.0 mg/mL
Bacillus subtilis	DSM10	MHB [2]	37	Oxytetracyclin 1.0 mg/mL
Mycobacterium smegmatis	ATCC 700084	7H9+ADC [3]	37	Kanamycin 1.0 mg/mL
Staphylococcus aureus	DSM346	MHB [2]	37	Oxytetracyclin 1.0 mg/mL
Pseudomonas aeruginosa	PA14	MHB [2]	37	Gentamycin 1.0 mg/mL

[1]MYC: 1 % w/v, bacto peptone, 1% w/v yeast extract, 2 % w/v glycerol, pH 6.3; [2]MHB: Müller-Hinton Broth (SN X927.1, Carl Roth GmbH, Karlsruhe, Germany); [3]7H9+ADC: Middlebrook 7H9 Broth Base + Middlebrook ADC Growth Supplement (SN M0678+M0553, Merck, Darmstadt, Germany);

Antimicrobial assay protocol

The assay was conducted as a minimum inhibitory concentration (MIC) assay in 96-well round bottom microtiter plates using the parameters summarized in Table S7 and as already described in [31].

Stocks of the test organisms were generated by growing the organisms overnight in 50 mL shaking flasks filled with 25 mL of the growth medium at 140 rpm (for media and temperatures see Table S38). If the organisms were well grown the next day, which was checked by occurrence of an optical density (OD)>30 of the suspension (OD600 nm for bacteria, OD548 nm for fungi and *M. smegmatis*), aliquots of these were stored in 1.5 mL reaction tubes in a freezer at −80 °C for up to 12 months. Upon use, aliquots were un-thawed, and the OD of the suspension measured and adjusted by diluting with the respective growth medium. OD600 nm was adjusted to 0.01 and OD548 nm to 0.1.

Subsequently, 150 µL of the adjusted suspensions were added to all wells of a 96-well microtiter plate (one test organism per plate). In row A, additional 130 µL of suspensions plus 20 µL of the test compounds (1 mg/mL) and the controls (one compound/column) were added. The test compounds were dissolved in MeOH, MeOH was used as negative control, while different positive controls (references) were used for the test organisms (see Table S38). Then, starting from row A, 150 µL of the suspension were transferred to the next row, the contents thoroughly mixed, and 150 µL transferred to the following row. The remaining 150 µL after row H were discarded. This resulted in a serial dilution of the test compounds, ranging from 66.7 µg/mL in row A to 0.52 µg/mL in row H. The microtiter plates were then incubated overnight on a microplate shaker at 800 rpm at 30 or 37 °C (see Table S38) and were visually evaluated the next day. The MIC is defined as the lowest concentration where no growth of the test organism was observed. A lower MIC thus corresponds to a higher antimicrobial activity of the test compound.

31. Kemkuignou, B. M.; Treiber, L.; Zeng, H.; Schrey, H.; Schobert, R.; Stadler, M.; Macrooxazoles a–d, new 2,5-disubstituted oxazole-4-carboxylic acid derivatives from the plant pathogenic fungus *Phoma macrostoma*. *Molecules* **2020**, *25* (23), 1–18. https://doi.org/10.3390/molecules25235497.

Table S3: Cytotoxicity assay parameters

cell line	type	No.	growth medium
L929	mouse fibroblasts	ACC 2	DMEM [1] + 10 % FBS [2]
KB 3.1	Human endocervical adenocarcinoma (AC)	ACC 158	DMEM [1] + 10 % FBS [2]
A431	Epidermoid carcinoma cells	ACC91	DMEM [1] + 10 % FBS [2]
A549	Adenocarcinomic human alveolar basal epithelial cells	ACC107	DMEM [1] + 10 % FBS [2]
PC-3	Postrate cancer cells	ACC465	F12K [1] + 10 % FBS [2]
MFC-7	Breast cancer cells	ACC115	RPMI [1] + 10 % FBS [2]
SKOV-3	Ovarian cancer cell line		McCOYs 5A

Cytotoxicity assay protocol

The assay was conducted in 96-well flat-bottom microtiter plates using the parameters summarized in Table S39.

Cell lines were incubated at 37 °C under 10 % CO_2 in Gibco™ DMEM medium (Thermo Fisher Scientific, Waltham, MA, USA) supplemented with 10 % FBS. A microtiter plate was filled with 120 µL of this suspension (50,000/mL) in each well.

Separately, another microtiter plate was filled with 100 µL of growth medium in each well. Then, 50 µL of the test compound solutions (1 mg/mL) were given to wells of the first column in two replicates (one compound per row). Cells without additives, MeOH were used as negative control. Starting from the first column, 50 µL of the solutions were gradually transferred to the next column, the contents thoroughly mixed, and 50 µL transferred to the following column. This created a serial dilution of the test compounds ranging from 333 µg/mL to 1.9×10−3 µg/mL. The remaining 50 µL after column twelve were discarded. From this microtiter plate, 60 µL of the solutions from 111 µg/mL to 1.9×10−3µg/mL were given to the first plate containing 120 µL of the cell suspensions (i.e. the highest concentration 333 µg/mL was not used). This resulted in final compound concentrations ranging from 37 µg/mL to 0.6×10−3 µg/mL.

After 5 days of incubation under the aforementioned incubation conditions, the half maximum inhibitory concentrations (IC50) were determined using a colorimetric tetrazolium dye MTT assay [S3]. For this, 20 µL of a 5 mg/mL solution of 3-(4,5-dimethyl-2-thiazolyl)-2,5-diphenyl-2H-tetrazolium bromide (MTT) were added to each well and incubated for two hours at 37 °C. Then, the microtiter plate was centrifuged (3,000 rpm, 5 min) and the supernatant removed by holding the plate upside-down and gentle shaking. Afterwards, the wells were washed using 100 µL of phosphate buffered saline (PBS). The plate was again centrifuged and the supernatant removed as described before. Then, 100 µL of an isopropanol: HCl solution (1L isopropanol+4 mL HCl 37 % w/v) were added to the wells. After incubating for 10 min at ambient temperature, the absorption of the wells at 595 nm was measured with an Infinite® 200 Pro microplate reader (TECAN, Männedorf, Schweiz).

Table S4: Antimicrobial activities of compounds **1-6**.

	Compounds (µg/mL)						Positive Control (µg/mL)	
**Strain**	**1**	**2**	**3**	**4**	**5**	**6**		
Bacillus subtilis DSM 10	-	-	-	-	-	-	4.2	Oxytetracyclin
Chromobacterium violaceum DSM 30191	-	-	-	-	-	-	0.83	Oxytetracyclin
Escherichia coli DSM 1116	-	-	-	-	-	-	1.7	Oxytetracyclin
Acinetobacte baumanii DSM 30008	-	-	-	-	-	-	0.26	Oxytetracyclin
Mycobacterium smegmatis ATCC 700084	-	-	-	-	-	-	1.7	Kanamycin
Pseudomonas aeruginosa PA14	-	-	-	-	-	-	0.21	Gentamycin
Staphylococcus aureus DSM 346	-	-	-	-	-	-	0.21	Oxytetracyclin
Candida albicans DSM 1665	-	-	-	-	-	-	4.2	Nystatin
Mucor hiemalis DSM 2656	-	-	-	66.7	-	-	8.3	Nystatin
Pichia anomala DSM 6766	-	-	-	-	-	-	4.2	Nystatin
Rhodoturula glutinis DSM 10134	-	-	-	66.7	-	-	1.0	Nystatin
Schizosaccharomyces pombe DSM 70572	-	-	-	-	-	-	4.2	Nystatin

(-) no activity; starting concentration for antimicrobial assay and cytotoxicity assay were 66.7 and 300 µg/mL, respectively.

Table S5: Cytotoxicity of compounds **1-6**.

**Cell line**	**Compounds (IC50, μg/mL)**						**Positive Control (μg/mL)**	
	**1**	**2**	**3**	**4**	**5**	**6**		
L929	-	/	/	33	-	-	2.4×10^{-4}	epothilone B
KB 3.1	-	/	/	-	-	-	1.7×10^{-5}	epothilone B
A431	/	/	/	-	/	/	2.4×10^{-5}	epothilone B
A549	/	/	/	16	/	/	3.4×10^{-5}	epothilone B
PC-3	/	/	/	-	/	/	4.8×10^{-5}	epothilone B
MFC-7	/	/	/	-	/	/	1.5×10^{-5}	epothilone B
SKOV-3	/	/	/	-	/	/	1.3×10^{-5}	epothilone B

(/) no tested

(-) no altered cells, no cytotoxic effect
